# Genetic Determinants of Antibody Levels in Cerebrospinal Fluid in Multiple Sclerosis: Possible Links to Endogenous Retroviruses

**DOI:** 10.3390/ijms19030786

**Published:** 2018-03-09

**Authors:** Alexander Emmer, Christine Brütting, Malte Kornhuber, Martin S. Staege

**Affiliations:** 1 Department of Neurology, Martin Luther University Halle-Wittenberg, Ernst-Grube-Str. 40, 06120 Halle (Saale), Germany; christine.bruetting@uk-halle.de (C.B.); malte.kornhuber@uk-halle.de (M.K.); 2 Department of Surgical and Conservative Pediatrics and Adolescent Medicine, Martin Luther University Halle-Wittenberg, Ernst-Grube-Str. 40, 06120 Halle (Saale), Germany; martin.staege@uk-halle.de

**Keywords:** multiple sclerosis, oligoclonal bands, immunoglobulin G index, genetic determinants, endogenous retrovirus (ERV)

## Abstract

The pathogenesis of multiple sclerosis (MS) has not been clarified. In addition to environmental factors; genetic determinants have been implicated in the pathogenesis of MS. Furthermore, endogenous retroviruses (ERV) might play a role in MS. The presence of oligoclonal immunoglobulin in cerebrospinal fluid (CSF) is a typical feature of MS. Recently, genetic polymorphisms in loci on human chromosomes 6, 14 and 18 have been identified as major determinants of CSF antibody levels in MS. The functional relevance of these single nucleotide polymorphisms (SNPs) remains unclear and none of them is located in an open reading frame. In previous studies, we identified ERV sequences in the vicinity of MS associated SNPs. Here, we describe the identification of ERV sequences in the neighborhood of SNPs associated with CSF antibody levels. All of the identified SNPs are located in the vicinity of ERV sequences. One of these sequences has very high homology to a sequence derived from the so-called MS-associated retrovirus (MSRV). Another cluster of three ERV sequences from the immunoglobulin heavy chain locus has retained the typical organization of retroviral genomes. These observations might shed new light on a possible association between ERVs and MS pathogenesis.

## 1. Introduction

Multiple sclerosis (MS) is a disease that is characterized by central nervous system demyelination with concomitant destruction of neurons. The causes of MS are unknown. However, during the course of MS, an activation of the immune system takes place and inflammation plays a major role in disease activity. The presence of oligoclonal immunoglobulin in the cerebrospinal fluid (CSF) is a typical feature of MS and so-called oligoclonal bands (OCBs), as detected by isoelectric focusing, are of diagnostic importance in MS [1]. The pathogenesis of MS OCB is still unresolved. OCBs have also been reported in the CSF of patients with different viral diseases like e.g., in human immunodeficiency virus (HIV) infected individuals [2,3,4]. Recently, genetic polymorphisms in loci on human chromosomes 6, 14 and 18 have been identified as major determinants of CSF antibody levels in MS [5]. The functional relevance of these single nucleotide polymorphisms (SNPs) remains unclear and none of them is located in an open reading frame. Previously, we have put forward the hypothesis that inflammation in MS, including humoral parts, could be driven by one or more endogenous retrovirus (ERV) elements [6]. If so, MS OCB should be somehow linked to ERV loci in the human genome. If ERV elements do have an impact on the manifestation of OCB in MS, their genomic representation might be related to identified SNP loci [7]. Therefore, we were interested in whether the identified genetic determinants with high and reproducible impact on the presence of autochthonous IgG synthesis in MS patients would fit this hypothesis.

## 2. Results

**Figure 1:**
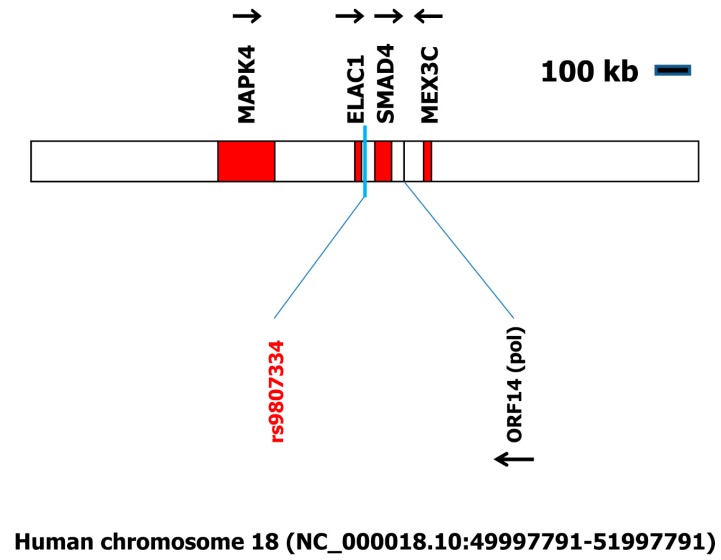
Schematic organization of the locus on human chromosome 18 including rs9807334. The two megabase pairs flanking rs9807334 were analyzed using getorf for the presence of open reading frames (ORFs) with a minimal length of one kilobase. Sixteen ORFs were identified (see Supplementary Table S1). These ORFs were analyzed using BLASTP against the database of retro-transcribing viruses (taxid:35268). One ORF with high sequence similarity to retroviruses was identified. This ORF is located between the SMAD family member 4 (SMAD4) locus and the Mex-3 homolog C (MEX3C) locus (for a complete list of all genes in this chromosomal region see Supplementary Table S2. ELAC1: elaC-like short form of RNase Z; MAPK4: mitogen-activated protein kinase 4; MEX3C: mex-3 RNA binding family member C; SMAD4: small body size and mothers against decapentaplegic homolog 4. Arrows indicate the direction of the ORFs.

**Figure 2:**
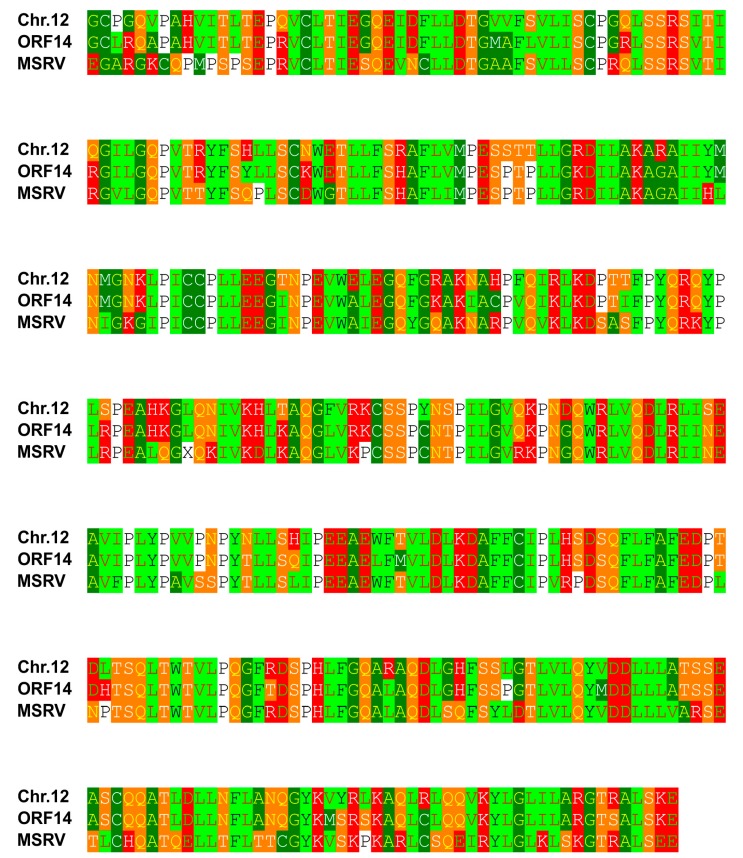
Multiple sequence alignment of MS associated retrovirus MSRV and predicted ORFs from chromosomes 12 and 18. The two megabase pairs flanking rs9807334 on chromosome 18 were analyzed using getorf for the presence of open reading frames (ORFs) with a minimal length of one kilobase. Sixteen ORFs were identified (see Supplementary Table S1). These ORFs were analyzed using BLASTP against the database of retro-transcribing viruses (taxid:35268). One ORF with high sequence similarity to retroviruses was identified. Presented is an alignment of this ORF (ORF14) and a polyprotein sequence from the multiple sclerosis associated retrovirus (MSRV; accession number AAB66528.1:6-379) and a similar sequence from chromosome 12 (Chr.12; accession number EAW96383.1).

On chromosome 18 (nucleotide accession number NC_000018.10:49997791-51997791), we found 16 large open reading frames (ORFs) in the two megabase pairs surrounding the SNP rs9807334. Only one of these ORFs showed high sequence similarity to proteins from retro-transcribing viruses (Figure 1, Supplementary Table S1). Interestingly, this ORF encodes a polymerase with very high homology (expect value 0.0) to a sequence identified in the so-called MS-associated retrovirus (MSRV) [8]. A similar sequence of identical length is present on chromosome 12 in the human genome (Figure 2).

**Figure 3:**
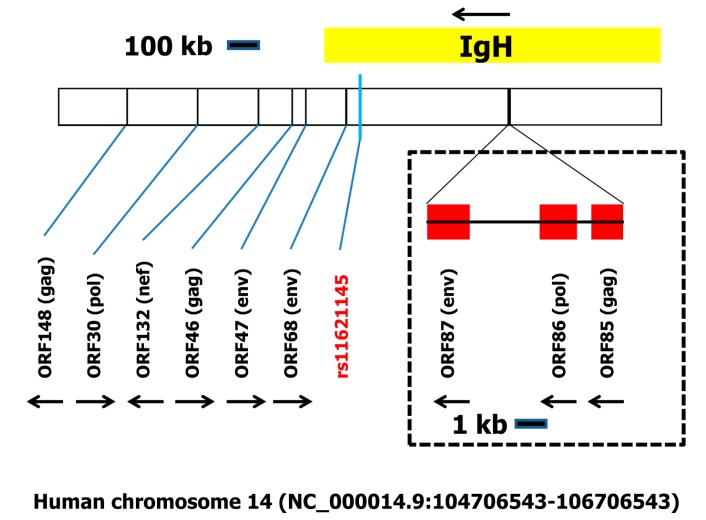
Schematic organization of the immunoglobulin locus including rs11621145 on human chromosome 14. The two megabase pairs flanking rs11621145 were analyzed using getorf for the presence of open reading frames (ORFs) with a minimal length of one kilobase. One hundred and sixty-nine ORFs were identified (see Supplementary Table S3). These ORFs were analyzed using BLASTP against the database of retro-transcribing viruses (taxid:35268). Nine ORFs with sequence similarity to retroviruses were identified. A cluster of three ORFs forming a retrovirus-like composite element is located between the HOMER homolog 2 pseudogene 2 and the solute carrier family 20 member 1 pseudogene 2 in the immunoglobulin heavy chain (IGH) locus. For a complete list of all genes in this chromosomal region, see Supplementary Table S4. Arrows indicate the direction of the ORFs.

On chromosome 14 (NC_000014.9:104706543-106706543), we found a large number of ORFs surrounding rs11621145 (Supplementary Table S3). From these ORFs, nine showed some similarity to retroviral proteins (2 × polymerase (pol), 3 × group specific antigen (gag), 3 × envelope protein (env), and 1 × HIV negative regulatory factor (nef)). Most of these ORFs showed only low sequence similarities to retro-transcribing viruses. However, a cluster of three large ORFs (ORF85-87 in Supplementary Table S3) encodes putative polypeptides with high homologies to gag, pol, and env proteins from retroviruses, respectively. These three ORFs showed the typical topology of the gag-pol-env organization of retroviruses and are located between the immunoglobulin heavy chain variable region genes IGHV3-19 and IGHV3-20 (Figure 3).

In the major histocompatibility complex region on chromosome 6, a combination of SNPs has been associated with CSF immunoglobulin levels [5]. Therefore, we included the complete region one megabase pairs upstream and one megabase pairs downstream of the rs6457617 and rs9271640 in our analysis (accession number NC_000006.12:31624423-33696074). We found 27 open reading frames surrounding the two polymorphisms (Supplementary Table S5). Only one of these ORFs showed homology to retroviral sequences. This ORF (ORF15 in Supplementary Table S5) showed high homology to envelope proteins from endogenous retroviruses. 

## 3. Discussion

Genome wide association studies currently enlarge our knowledge and understanding of disease pathogenesis [9]. However, the exact functions and especially the interactions between disease-associated polymorphisms have not been completely elucidated. Genetic determinants of antibody levels in the CSF of patients with MS have been identified in a couple of loci that were associated with the presence of OCB or elevated IgG indices [5].

Polymorphisms on chromosomes 6, 14 and 18 showed the strongest association with OCB and/or immunoglobulin indices [5]. The signal from chromosome 14 was located in the immunoglobulin heavy chain locus near the immunoglobulin alpha 1 element (see Supplementary Table S4). Although the most significant signal from chromosome 6 was highly correlated with HLA-DRB1*1501, this signal finds itself clearly distant from HLA genes. Similarly, the signal from chromosome 18 maps to the intergenic region between the genes ELAC1 (encoding the short form of RNase Z) and SMAD4 (*C. elegans* small body size (SMA) and *Drosophila* mothers against decapentaplegic (MAD) homolog 4). It seems likely that the identified markers represent tagging polymorphisms that are linked to yet unknown functional elements. 

The analyses of the chromosomal regions surrounding the identified polymorphisms yielded the presence of a variety of putative ERV sequences. In the surroundings of the SNP rs9807334 on chromosome 18, the ORF (see Figure 1) that encodes a protease and reverse transcriptase has a very high homology to a sequence identified in MSRV [8]. The corresponding original nucleotide sequence (GenBank accession number AF009668) is not present in the human reference genome. It has been suggested that MSRV sequences might arise by (probably post-transcriptional) recombination between different MSRV-like loci in the human genome [10]. The location of rs9807334 in the vicinity of a protease/polymerase open reading frame with such high homology to the isolated MSRV could support the hypothesis that ERV activity is involved in the pathogenesis of MS.

**Figure 4:**
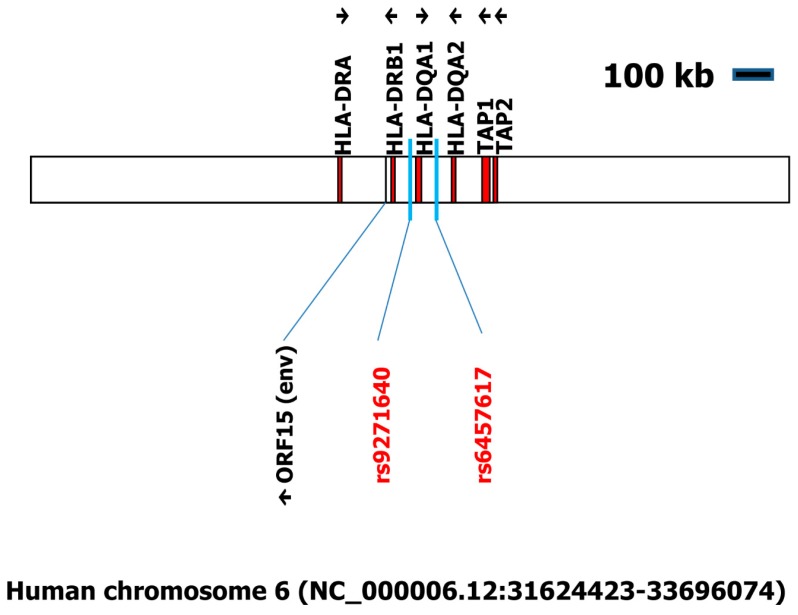
Schematic organization of the rs9271640/rs6457617 region at human chromosome 6. The 2,071,651 base pair region on human chromosome 6 flanking the rs9271640/rs6457617 region was analyzed for the presence of open reading frames (ORFs) with a minimal length of one kilobase by using getorf. 27 ORFs were identified (see Supplementary Table S5). These ORFs were analyzed using BLASTP against the database of retro-transcribing viruses (taxid 35268). One of the ORFs (ORF15) showed high homology to known sequences from retro-transcribing viruses. For a complete list of all genes in this chromosomal region, see Supplementary Table S6. TAP: transporter associated with antigen processing. HLA: human leukocyte antigen.

The SNP on chromosome 14 (located in the immunoglobulin heavy chain locus) and chromosome 6 (located in the MHC) might be more directly involved in the regulation of the immune response and antibody production. Nevertheless, these loci also contain retrovirus-like open reading frames (Figure 3 and Figure 4). The investigated region on chromosome 14 contains multiple ERV-like sequences. Of special interest is the trio of ERV-sequences that are located in the immunoglobulin heavy chain locus. These ORFs include the complete set of sequences (gag, pol, env) typically found in retroviruses. The location of this trio in the chromosomal region containing the immunoglobulin heavy chain variable (V) elements (see Supplementary Table S4) indicates that, during B cell development, immunoglobulin rearrangements can substantially reduce the distance of these elements from the SNP rs11621145. Immunoglobulin class switching can further reduce this distance or can lead to excision of the rs11621145 region.

The SNPs on chromosome 6 are located in the human major histocompatibility complex (MHC) class II region. The adjacent ORF that encodes a putative envelope protein has high similarity to other human ERVs. Nearly identical sequences have been detected on other human chromosomes (EAW95690 [11], AAB65470, AAB65469 [12]). In addition, the sequence has some similarity with other members of the K family including HERV-K113 and HERV-K18. The HERV-K113 sequence is not present in all individuals [13]. HERV-K113 positivity was found in some studies to be associated with MS and other autoimmune diseases [14,15]. However, other studies were unable to found this association [16].

The interaction of different MS susceptibility loci is already known and may lead to considerable MS-risk amplification [17,18]. The question of how ERV elements interfere with OCB associated loci and result in production of intrathecal OCB IgG remains to be established. It is known that retroviral envelope proteins like gp120 of HIV activate B cells to produce antibodies [19]. In fact, at least 95% of the oligoclonal IgG synthesis within the CSF of HIV positive patients is not directed against HIV antigens [20]. Therefore, it is not far to speculate that a major part of these antibodies may be due to B cell stimulation, e.g., by the HIV envelope protein gp120. From the putative ERV sequences detected in our study, only two (ORF87 from Supplementary Table S3 and ORF15 from Supplementary Table S5) have high sequence similarities to retroviral envelope proteins. In both cases, the detected ORFs encoded only the C terminal part of an envelope including the typical heptad repeat 1/2 ectodomain region. The ORFs encode approximately 69% (ORF87) and 49% (ORF15) of a typical envelope protein. Whether these sequences are translated into proteins have to be investigated in further studies. If such sequences are translated into functional envelope proteins, it might be possible that these factors stimulate B cells and therefore influence antibody production in these cells.

## 4. Materials and Methods

SNPs to analyze were selected on the basis of a published study concerning associations between SNPs and CSF immunoglobulin levels in MS patients [5]. From this study, only SNPs were considered if they showed significant associations with CSF immunoglobulin levels in the screening phase as well as in the replication phase. Only 4 SNPs fulfilled this criterion: rs9807334 from chromosome 18, rs11621145 from chromosome 14, and the two SNPs rs9271640 and rs6457617 from chromosome 6. Analysis of the chromosomal regions surrounding these SNPs for the presence of putative ERV sequences was essentially performed as previously described [7]. Genomic SNP sequences were retrieved from the NCBI SNP data base (https://www.ncbi.nlm.nih.gov/projects/SNP/) and blasted against the current human reference genome (NC_000014.9) using NCBI nucleotide BLAST [21]. The two megabase pairs surrounding the SNPs were analyzed with getorf (http://bioinfo.nhri.org.tw/cgi-bin/emboss/getorf) [22] for the presence of large (>999 nucleotides) open reading frames. The identified open reading frames were tested for sequence similarity to retro-transcribing viruses (taxid 35268) using BLASTP [21]. Gene annotations were retrieved from the reference genome sequence (chromosome 14: NC_000014.9).

## 5. Conclusions

Taken together, retroviral elements exist in the relatively close vicinity of major genetic determinants for OCB and for IgG indices in MS. The precise meaning of how these ORFs with homology to retroviruses with proposed involvement in MS pathogenesis may come into play remains to be established.

## Supplementary Materials

Supplementary materials can be found at http://www.mdpi.com/1422-0067/19/3/786/s1.
