# Supplementary material for: Genetic Determinants of Antibody Levels in Cerebrospinal Fluid in Multiple Sclerosis: Possible Links to Endogenous Retroviruses
Source: Int J Mol Sci. 2018 Mar 9;19(3):786. doi: 10.3390/ijms19030786 (PMC5877647; doi:10.3390/ijms19030786)
Supplement: Supplementary file 1 [file ijms-19-00786-s001.zip › Supplementary Table S1.docx]

**Supplementary Table S1. Large ORFs surrounding rs9807334 on human chromosome 18**. The two megabase pairs flanking rs9807334 were analyzed using getorf for the presence of open reading frames with a minimal length of one kilobase. 169 open reading frames were identified. These ORFs were analyzed using BLASTP against the database of retro-transcribing viruses (taxid 35268).

| **ORF number [position in sequence]**  **sequence** | **Representative**  **BLASTP hits** |
| --- | --- |
| **ORF1** [150832 - 152835] QNSTTFMLKTLNKLGIDGTYLKIIRAIYDKPTANIILNGQKLEAFPLKTGTRQGCPLSPLLFNIVLEVLARAIRQEKEIKGIQLGKEEVKLSLFADEMIVYLENPTVSAQNLLKLISNFSKVSGYKINVQKSQAFLYTNNRQTESQIMSELPFTIASKRIKYLGIYLTRDMKDLFKENYKPLLNVIKEDTNKWKNIPCSWIGRINIVKMAILPKVIYRFNAIPIKLPMTFFTELEKTTLKFIWNQKRAHIAKSILSQKNKAGGIMLPDFKLYYKATVTKTAWYWYQKRDIDQWNRTEPSEIMPHIYNHLIFDKPEKNKQWGKDSLFNKWCWENWLAICRKLKLDPFLTPYTKINSRWIKDLHVRPKTIKTLEENLGITIQDTGTGKDFMSKTPKAMARAKIDKWDLIQLKCFCTAKETTIRVNRQPTKWEKIFTTYSSDKGLISRIYNELKQIYKKKTNNPIKKWAKDMNRHFSKEDICAAKKHMKKCSPSLAIREMQIKTTMRYHLTPVRMAIIKKSGNNRCWRGCGEIGTLLHCWWDCKLVQPLWKSVWRFLRDLELEIPFDPAIPLLGIYPKDYKSCCYKDTCTRMFIAALFTIAKTWNQPKCPTMIDWIKKMWHIYTMEYYAAIKNDEFMSFVGTWMKLEIIILSKLSQEQKTKHRIFSFIGGN | **none** |
| **ORF2** [561574 - 562974] GPVASSVPFLWIPKCWFSFVSFIPVMPMSTDSQPRRPARNPRPAGDRCTWAPRPLDPRPSAPSRARGSPRFQSPPVPAGSFSPPTLPPGPRRLQPRGAGGAATGLRQIPASRLLGAPSLPDGARGSPTHGGREEGLLSDWSSTACAPGRPGYPGRGSAPQVSSSPRDWVREGPRTARLSGLEAAGGQSRARGRLPPPGAQGPAPQRHRRSRWGPSGRGRGPALGGAEAAAAQAGAGAGAGAGARAGAASRAVGATAGARRAPWLGPAWPSAPAPRPQTKGGSRPGRHALGLCLGKWLPSAGFLLPPPAPLGVRRAPEKRGRDETSPPALPPHLTLTISPPGQATAKLASFRAAGVSRGTRPAALGELLACRPRAGAVLDPFGMGGCRWAPRVVFRGGGRRGRAPLGDPTPGDSPERLGSPRGSSHCEFRFGSQSRLSSPPASPFLVEGLIQRPLPSPPDRGAYTGGR | **none** |
| **ORF3** [628444 - 629547] CLTKYLGTVVNQVDIKLTFTPGKSISEREVCGGRKCCGPDGQSRLPGRGGLEPTLGMAWASWRLRSLHTEPQRGRGVCPIALLEAGRILGWPKGLVTFGPRVHQGVTCGPLAPTLSLSGNLFFLFLVPSPLPFLITLSSRLLPQVKPGTRPGPSGSQEVTRCLQQMCFIQILSMNTACPTPKSSRCSGGSVVCSPTLCPSDSLRLHVVSFIMQVYFVKGAPLLTAQGNQPPGYLLSLVNLQDKWVLSLGLVTVKGRESITIPAWHFCPPYHPAPRPLILATWPPFPLLLCFLSWLLPRLPVDHMVRGGFLGEADLDQPWGMAWVSWHLLSLHIEPHGGLGSLSHGLIGGGKDSEGRIPLRGKVLKARS | **none** |
| **ORF4** [1132326 - 1133696] TVPISWFPTTTLFFPFFFDTESRSVAQAGVQWRDLGSLQAPPPGFKQFSCLSLPSSWDYRRLPPHVANFCIFCRDGVSPCWPGWSQTPDFRRSTHFSLPKCWDYRHEPPCLAILPHFLDANISNSELYIYIHTHIHIHIYTHTYIHTYIHTYIHTYIHIYTYIYTHIYIHIYTYIHTCIYTHTYMYTHIHTCIRTHIHTCIRTHIHTCIRTHIHIHVYAHTYTYMYTHTYTHTCIRTHIYIHVYAHIYTYMYTHTYTYMYTHTHTYMYTHTHTYMYTHTHTYMYTHTHTYMYTHTYTYMYTHTYTYMYIHTYIHVYTHIHTYMYIHTYIHVYTHIHTYMYIHTYIHVYTYMYIHTYIYIHTCIYIHIYTYMYIYIHVYIHTYMCIHTYMCIHTYIHVYTYIHVYIYIYTYMCIYTYIHTCIYVYIYIFFETRSHFVTQSGVQWCYHGSMHPQSPRLK | **none** |
| **ORF5** [1132693 - 1133775] ATLNYIYIYTHTYTYIYIHIHIYTHIYIHIYIHIYTYIHTYIHIYTYIYTHTYIHVYTHIHTCIHTYIHVYAHIYIHVYAHIYIHVYAHTYTYMYTHTHTHTCIRTHIHIHVYAHIYTYMYTHTYIHTCIRTHIHTCIRTHIHTCIRTHIHTCIRTHIHTCIRTHIHTCIRTHIHTCIRTHIHTCIYTHTYMYIHTYIHTCIYTHTYMYIHTYIHTCIYTHTYMYIHTCIYIHIYTYIHVYTYIYIHTCIYTYMCIYIHTCVYIHTCVYIHTYMCIHTYMCIYTYIHTCVYIHIYIHVYMYIYIYFLRQGLTLLPSLEYSGATMAPCILNLPGSSDRFASASRVAGTTGMCNHTWLSFFLK | **none** |
| **ORF6** [1746734 - 1748251] QTHSQYHTEWAKTGSIPFENGHKTGMPSLTTPVQHSVGSSGQGNQAGEGNKGYSIRKKEEVKLSLFADDMIVYLENPIVSAQNLLKLISNFSKVSGYKINVQKSQAFLYTNNRQTESQIMSELPFTIASKRIKYLGIQLTRDVKDLFKENYKPLLKEIKEDTNKWKNIPCSWVGRINIVKMAILPKVIYRFNAIPIKLPMTFFTELEKTTLKFIWNQKRAHIAKSILSQKNKDGGITLPDFKLYYKATVTKTAWYWYQNRDIDQWNRTEPSEITPHTYNYLIFDKPEKNKQWGKDSLFNKWCWENWLAICRKLKLDPFLTPYTKINSRWIKDLNVRPKTIKTLEENLGITIQDIGVGKDFMSKTPKAMATKAKIDKWDLIKLKSFCTAKETTIRVNRQPTTWEKIFATYSSDKGLISRIYNELKQIYKKKTNNPIKKWAKDMNRHFPKEDIYAAKKHMKKCSSSLVIREMQIKTTMRYHLTPVRMAIIQKSGKNRCWRGCGEIGTL | **none** |
| **ORF7** [1832113 - 1833291] QTERQIMSELPFTIASKRIKYLGIQLARDVKDPFKENYKPLLNEIKEDTNKWKNIPCSWVGRINIVKMAILPKVIYRFNAIPIKLPMTFFTEFEKTTLKFIWNQKRAHIAKSILSQKNKAGGITLPDFKLYYKATVTKTAWHWYQNRDIDQWNRTEPSEIMPHIYNYLIFDKPDKNKQWGKDSLFNKWCWENWLAICRKLKLDPFLTPYTKINSRWIKDLHVRPKTIKTLEENLGNTIQDIGMGKDFMSKTPKAIATKAKINKWDLIKLKSFCTAQETTIRVNRQPTEWEKIFATYSSDKGLISRIYNEVKQIYKKKTNNPIKKWAMDMNRHFSKEDIYAAKKHRKECSSSLAIREMQIKTTMRYHLTPVRMAIIKKSGNNRCWRGCGEIGTL | **none** |
| **ORF8** [1970381 - 1969338] (REVERSE SENSE) KDSLFNKWGWENWLAICRKLKLDPFLTPYTKINSRWIKDLNVRTKTIETLEENLGNTIQDTGMGKDFMSKTPKAMATKGKIDKWDLIKLKSFCTAKETTIRVNRQPTEWEKIFAIYSSDKRLISSIYNELKQIYKKKTNNPIKKWAKDMNRHFSKEDIYAANRHMKKCSSSLVIGEMQIKTTMRYHLTPVRMAIIKKSGNNRCWRGCGEIGTLLHCWWDCKLVQPLWKSVWQSLRDLELEIPFDPAIPLMGIYPKDYNSCCYKDTCTHMFIVALFTIAKTWNQPKCATMIDWIKKTWHIYTMEYYAAIKNDEFMSFVGTWMTLETIILSKLSQGQKTKRCLFSLIGGN | **none** |
| **ORF9** [1849677 - 1848616] (REVERSE SENSE) ELNHFYSSCTHTYTHTHTYTHTQILRNVPNQGGERPREGELQKAAERNHREHKKWKYISCSWMGIINIVKMTILPKAIYKFNAIPIKIPPSSFTELEKTIPKFIWNQKKAHIAKANLSKKNKSGGITLSGCKLYHKAILTKTVWYWHKNRCIDQWNRIENPEINPKTYSQLIFDKTNKNLKWKKHTLFNKWCWNYWLATCKRMKLDPHLSPYTKINSGWIKDLNLRTKTIKILEDNIGKPLLDIGIGKDFMTKNPKASAIKTKINSWDLIKLKSFCMAKGMLSRANIQSKEWEKIFTIYTSDKGLISRIYNEHEQISKKKTNNPIKQWAKDMDRQFSKEDIQINNKHEKNAQHH | **none** |
| **ORF10** [1704183 - 1703074] (REVERSE SENSE) NRDIDQWNRTEPSEIIPHIYNHLIFDKPDKNKKWGKDFLFNTWCWENWLAMCRKLKLDPFLMPYTKINSRWIKDLNVRPKTIKTLEENLGNTIQDIGMGKDFISKTPKAMATKARIDKWDLIKLKSFCTAKETTIRVKRQPTEWEKIFATYSSDKGLISRIYKELKQIYKKKKNIKKWAKHMNRHFSKDDIYAANRHMKKCSSSLAIREMQIKTTMRYHLTSVKMSIIKKSGNNRCWRGCGKIGTLLQCWWDCKLVQTLWKSVWRFFKGLELEIPFDPAIPLLGIYPKDYKSCYCKDTCTRMLIVALFTIANTWNQPKCSSVIDWIKKMWHSWAQWLTPVIPAFWEAKAGGSEGQEIETILANMVKPRLY | **none** |
| **ORF11** [1179805 - 1178564] (REVERSE SENSE) NVFFLTGCKIKALRAKTNTYIKTPVRGEEPIFVVTGRKEDVAMAKREILSAAEHFSMIRASRNKNGPALGGLSCSPNLPGQTTVQVRVPYRVVGLVVGPKGATIKRIQQQTHTYIVTPSRDKEPVFEVTGMPENVDRAREEIEMHIAMRTGNYIELNEENDFHYNGTDVSFEGGTLGSAWLSSNPVPPSRARMISNYRNDSSSSLGSGSTDSYFGSNRLADFSPTSPFSTGNFWFGDTLPSVGSEDLAVDSPAFDSLPTSAQTIWTPFEPVNPLSGFGSDPSGNMKTQRRGSQPSTPRLSPTFPESIEHPLARRVRSDPPSTGNHVGLPIYIPAFSNGTNSYSSSNGGSTSSSPPESRRKHDCVICFENEVIAALVPCGHNLFCMECANKICEKRTPSCPVCQTAVTQAIQIHS | **none** |
| **ORF12** [1133813 - 1132707] (REVERSE SENSE) DQPGKYSETPRIYFKKKLSQVWLHMPVVPATLEAEAKRSLEPGRLRMHGAMVAPLYSRLGNKVRPCLKKYIYIYIYTCMYICIYTHVCIYVYIHMYVCIHMYVCMYTHVCMYTHVCMYIHMYVYIHVCIYMYVYTCMYVYICMYIHVCIYMYVCVYIHVCMYVCIYMYVCVYIHVCMYVCIYMYVCVYIHVCICVRIHVCICVRIHVCMCVRIHVCMCVRIHVCMCVRIHVCMCVRIHVCICVRIHVCIYVCVYMYVYMCAYTCMCICVRIHVCVCVCVYMYVYVCAYTCMYMCAYTCMYMCAYTCMYVCIHVCMCVYTCMYVCVYMYVYMCIYVCIYVYICMYICMYICVYICMCIYMYVYVCVYIYI | **none** |
| **ORF13** [1133631 - 1132615] (REVERSE SENSE) QSETLSQKIYIYIHIYMYVYMYIYTCMYICIYTHVCMYTHVCMYVYTCMYVYTCMYVYTHVCIYTCMYIYVCIYMYVCIYMYVYTCMYIHVCMCVYTCMYVCVYIHVCMCVYTCMYVCVYIHVCMCVYTCMYMCAYTCMYMCAYTCMYVCAYTCMYVCAYTCMYVCAYTCMYVCAYTCMYMCAYTCMYICVRIHVCIYVCVYMYVYMCAYTCMCMCVRIHVCVCVCVYMYVYVCVYMYVYVCVYMYVCVYTCMYVCIYMYVCMCIYVCIYVYICMYICVYMYVYMYVYMCVYMYVYIYVCVCVCVYIYIVQSCLYLHQENAEGWPGMVAHACNPSTLGG | **none** |
| **ORF14** [1117905 - 1116784] (REVERSE SENSE) GCLRQAPAHVITLTEPRVCLTIEGQEIDFLLDTGMAFLVLISCPGRLSSRSVTIRGILGQPVTRYFSYLLSCKWETLLFSHAFLVMPESPTPLLGKDILAKAGAIIYMNMGNKLPICCPLLEEGINPEVWALEGQFGKAKIACPVQIKLKDPTIFPYQRQYPLRPEAHKGLQNIVKHLKAQGLVRKCSSPCNTPILGVQKPNGQWRLVQDLRIINEAVIPLYPVVPNPYTLLSQIPEEAELFMVLDLKDAFFCIPLHSDSQFLFAFEDPTDHTSQLTWTVLPQGFTDSPHLFGQALAQDLGHFSSPGTLVLQYMDDLLLATSSEASCQQATLDLLNFLANQGYKMSRSKAQLCLQQVKYLGLILARGTSALSKE | **polyprotein, partial [MSRV]**  **Sequence ID: AAB66528.1**  **E value: 0.0** |
| **ORF15** [351401 - 350280] (REVERSE SENSE) GSCLLEGKLTTRKDIYTENPSVHHHHQRPKVDKTTKMGKKQNRKTGNSKTQSASPPPKERSSSPATEQSCMENDFDELREEGFRRSNYSELREDIQTKGKEVENFEKNLEECITRITNTEKCLKELMELKTKARELREECRSLRSRCDQLEERVSAMEDEMNEMKREGKFREKRIKRNEQSLQEIWDYVKRPNLRLIGVPESDGENGTKLENTLQDIIQENFPNLARQANVQIQEIQRTSQRYSSRRATPRHIIVRFTKVEMKEKMLRAAREKGRVTLKGKPIRLTADLSAETLQARREWGPIFNILKEKNFQPRISYPAKLSFISEGEIKYFIDKQMLRDFVTTRPALKELLKEALNMERNNRYQPLHNHAKM | **none** |
| **ORF16** [350237 - 346389] (REVERSE SENSE) AKSPANIIMTGSNSHITILTLNINGLNSAIKRHRLASWIKSQDPSVCCIQETHLMCRDTHRLKIKGWRKIYQANGKQKKAGVAILVSDKTDFKPTKIKRDKEGHYIMVKGSIQQEELTILNIYAPNTGAPRFIKQVLSDLQRDLDSHTLIMGDFNTPLSTLDRSTRQKVNKDTQELNSALHQADLIDIYRTLHPKSKEYTFFSAPHHTYSKIDHIVGSKALLSKCKRTEIITNYLSDHSAIKLELRIKNLTQSRSTTWKLNNLLLNDYWVHNEMKAEIKMFFETNENKDTTYQNLWDAFKAVCRGKFIALNAYKRKQERSKIDTLTSQLKELEKQEQTHSKASRRQEITKIREELKEIETQKTLQKINESRSWFFERINKIDRPLARLIKKKREKNQIDTIKNDKGDITTDPTEIQTTIREYYKHLYANKLENLEEMDTFLDTYTLPRLNQEEVESLNRPITGSEIVAIINSLPTKKSPGPDGLTAEFYQRYKEELVPFLLKLFQSIEKEGILPNSFYEASIILIPKPGRDTTKKENFRPISLMNIDTKILNKILANRIQQHIKKLIHHDQVGFIPGMQGWFNIRKSINVIQHINRAKDKNHMIISIDAEKAFDNIQQPFMLKTLNKLGIDGTYFKIIRAIYDKPTANIILNGQKLEAFPLKTGTRQGCPLSPLLFNIVLEVLARAIRQEKEIKGIQLGKEEVKLSLFADDMIVYLENPIVSAQNLLKLISNFSKVSGYKINVQKSQAFLYTNNRQTESQIMSELPFTIASKRIKYLGIQLTRDVKDLFKENYKPLLKEIKEDTNKWKNIPCSWVGRINIVKMAILPKVIYRFNAIPIKLPMTFFTELEKTTLKFIWNQKRARIAKSILSQKNKAGGITLPDFKLYYKATVTKTAWYWYQNRDIDQWNRTEPSEIMPHIYNYLIFDKPEKNKQWGKDSLFNKWCWENWLAICRKLKLDPFLTPYTKINSRWIKDLNVRPKTIKTLEENLGITIQDIGVGKDFMSKTPKAMATKAKIDKWDLIKLKSFCTAKETTIRVNRPPTTWEKIFATYSSDKGLISRIYNELKQIYKKKTNNPIKKWVKDMNRHFSKEDIYAAEKYMKKCSSSLAIREMQIKTTMRYHLTPVRMAIIKKSGNNRCWRGCGEIGTLLHCWWDCKLVQPLWKSVWRFLRDLELEIPFDPAIPLLGIYPKDYKSCCYKDTCTRMFIAALFTIAKTWNQPKCPTMIDWIKKMWHIYTMEYYAAIKNDEFMSFVGTWMKLETIILSKLSQEQKTKHRIFSLIGGN | **none** |
