# Supplementary material for: Genetic Determinants of Antibody Levels in Cerebrospinal Fluid in Multiple Sclerosis: Possible Links to Endogenous Retroviruses
Source: Int J Mol Sci. 2018 Mar 9;19(3):786. doi: 10.3390/ijms19030786 (PMC5877647; doi:10.3390/ijms19030786)
Supplement: Supplementary file 1 [file ijms-19-00786-s001.zip › Supplementary Table S2.docx]

**Supplementary Table S2. Genes, pseudogenes, and large retroviral ORFs flanking rs9807334 on chromosome 18**. Presented are all annotated genes and identified ORFs in the two megabase pair region (nucleotide accession: NC_000018.10:49997791-51997791) surrounding rs9807334. Genes/ORFs located on the reverse strand are indicated by the orientation “c”). ORF14 was numbered according to Supplementary Table S1.

| **Orientation** | **Start** | **Stop** | **Gene symbol** | **Feature** |
| --- | --- | --- | --- | --- |
|  | <1 | 197291 | MYO5B |  |
|  | 66002 | 66696 | ADAD1P2 |  |
| c | 128709 | 128773 | MIR4320 |  |
| c | 209208 | 209485 | RN7SL310P |  |
| c | 229403 | 268732 | CFAP53 |  |
| c | 269092 | 283984 | MBD1 |  |
| c | 284553 | 290532 | CXXC1 |  |
|  | 321212 | 321330 | RNA5SP458 |  |
| c | 356219 | 377111 | LOC105372114 |  |
|  | 377232 | 396378 | SKA1 |  |
|  | 402348 | 412227 | LOC105372115 |  |
|  | 460024 | 461069 | RPLP0P11 |  |
|  | 561991 | 734036 | MAPK4 |  |
| c | 750379 | 754871 | LOC105372116 |  |
| c | 797330 | 827623 | MRO |  |
|  | 816598 | 817749 | HNRNPA3P16 |  |
|  | 825787 | 826314 | RPL17P46 |  |
|  | 880814 | 952005 | ME2 |  |
|  | 970227 | 990330 | ELAC1 |  |
| c | 980239 | 991022 | LOC107985152 |  |
|  | 1000001 | 1000001 | - | rs9807334 |
| c | 1027306 | 1032379 | LOC105372117 |  |
|  | 1032423 | 1087252 | SMAD4 |  |
| c | 1116784 | 1117905 | - | **ORF14** |
| c | 1160412 | 1161653 | SRSF10P1 |  |
| c | 1176760 | 1199891 | MEX3C |  |
| c | 1195116 | 1198759 | LOC105372118 |  |
| c | 1285943 | 1286100 | RNU1-46P |  |
|  | 1394252 | 1564679 | LOC100287225 |  |
| c | 1422863 | 1423451 | SS18L2P2 |  |
|  | 1435453 | 1437149 | LOC100420949 |  |
| c | 1614769 | 1631710 | RSL24D1P9 |  |
| c | 1647213 | 1658643 | LOC105372120 |  |
| c | 1689743 | 1690419 | LOC100422053 |  |
| c | 1847160 | 1847844 | RPS8P3 |  |
