# Supplementary material for: Genetic Determinants of Antibody Levels in Cerebrospinal Fluid in Multiple Sclerosis: Possible Links to Endogenous Retroviruses
Source: Int J Mol Sci. 2018 Mar 9;19(3):786. doi: 10.3390/ijms19030786 (PMC5877647; doi:10.3390/ijms19030786)
Supplement: Supplementary file 1 [file ijms-19-00786-s001.zip › Supplementary Table S3.docx]

**Supplementary Table S3. Large ORFs surrounding rs11621145 on human chromosome 14**. The two megabase pairs flanking rs11621145 were analyzed using getorf for the presence of open reading frames with a minimal length of one kilobase. 169 open reading frames were identified. These ORFs were analyzed using BLASTP against the database of retro-transcribing viruses (taxid 35268).

| **ORF number [position in sequence]**  **sequence** | **Representative**  **BLASTP hits** |
| --- | --- |
| **ORF1** [7378 - 8424] RWQERQAARPWWEDPIPGVAASGCWEWHGPVSPTLPQCRQTKDAEAGSCSETSEEAFWGGGFAGRSGRQRNGGGCTWAGSGAGCLPFTGVSLHPVATSNPAGDPVGSTRCPASEPGLDATTASESRGWDLVDAVTPGPQPTLEQLEEGGPRPLERRSSWYVDASDVLTTEDPQCPQPLEGAWPVTLGDAQALKPLKFSSNQPPAAGSSRQDAKDPTSLLGVLQAEADSTSEGLEDAVHSRGARPPAAGPGGDEDEDEEDTAPESALDTSLDKSFSEDAVTDSSGSGTLPRARGRASKGTGKRRKKRPSRSQEGNSGRGPGHRPTPGRWHPAGPSPSPLGTASSSGTQER | **none** |
| **ORF2** [94617 - 95921] RASRLCPGDDGGGMEFPEHGGRLLGRLRQQRELGFLCDCTVLVGDARFPAHRAVLAACSVYFHLFYRDRPAGSRDTVRLNGDIVTAPAFGRLLDFMYEGRLDLRSLPVEDVLAAASYLHMYDIVKVCKGRLQEKDRSLDPGNPAPGAEPAQPPCPWPVWTADLCPAARKAKLPPFGVKAALPPRASGPPPCQVPEESDQALDLSLKSGPRQERVHPPCVLQTPLCSQRQPGAQPLVKDERDSLSEQEESSSSRSPHSPPKPPPVPAAKGLVVGLQPLPLSGEGSRELELGAGRLASEDELGPGGPLCICPLCSKLFPSSHVLQLHLSAHFRERDSTRARLSPDGVAPTCPLCGKTFSCTYTLKRHERTHSGEKPYTCVQCGKSFQYSHNLSRHTVVHTREKPHACRWCERRFTQSGDLYRHVRKFHCGLVKSLLV | **none** |
| **ORF3** [144950 - 146143] SQVLRRLRQENRLNLGGEGCSEPRSRHCTPAWVTEQDSVSKKKKKKKRSDFISLNIEGMLSYQPWQATTHMCIRYHVCEALQGIHFFSHSTPVDVTVSAANRKTPSPAAVFHYDPQRVFSTFNHVGPTALGMALCHLVFLQTLPEFQENLRDHGKGYWAVQMLPSLRHLTSGICSLLPLPEGTLGNPAAGLTQERLREEMGSATRGPSERRFNSFTCADKAAVVSTAVKAAISGGWHAACTRINTSNRKTAKSSKANKDGWVLLTPPMPVPHPLSVPSFTSDSCVHSDVPKKSPTEWTHRKSYSRRMCHPLPFLPRLTLAMRCAWLLEWALHNDSRELRAAVSVLWVSGTWWRRFKGAFSNFTILREKTPHWVAPGSMVSGFDCRGGGVGGVDQHIFI | **none** |
| **ORF4** [152131 - 155265] KLLCLPFLSEQLSDIKCIHIVVQPSPSSISTVHLYCPLPPFIPTLHHHPPSPPSISTVYHHPPSPPSISTLRPPSISISTIHHHSPSPPSITTLHLHPPSPPTISTLHLHHPSPPSITTLHFHCPLPPFISTLYHHSPSPPSIHPPSPSSISTVHRHHSSPPSITTHHLHPPSPTISTLHLHHPSQPFISAVHHHPPFPPCISTLHFHPASPPSITTLHLHPPSPPSIATLHHQPPSPPSITTLHLHPPSPPSISTLHHHLPSPPFVHPPSPPSITTIHLHPSSTLHLHPPSPPTISTLHLHHPSQPFISAIHHHPPFPPCISTLHHHLPSPSSISTVHRHHSSPPSITTLHHHLPSPPFIHPPSPSSISTIHHHHSSPPSITTLHLHPPSPSSITTHHFHAPSPPSITTIHLRCPSPPSITTIHHHSPFRLSISTVHYHHLSPPSITTHHLHPPSTLHLHPPSPPSITTLHHYLPSPPFIHPPSPSSISIVHHHHSSPPSITTLHHHPPSPSSITTHHFHAPSPPSITTIHLRCPSPPSISTLHLHPPFPLSISTVHYHHLSPPSITTHHLHPPSTLHLHPPSPPTISTLHLHHPSQPFISTVHHHPPFPPCISTLHLHPSSPPFISTIHHHPPSSPTISTLHHHPLSPPSISTLHPPSISILHHHQLFLPSISTIHHHPPSPPPITTLHHQPPSPPTISTLHLHCPSLPSTLHHHPPSPPSITAIHHHPPSPPFNSTIHVHHPSPPSISTVHVHHPSPPSISTVHHHPLSPASMSTVHHHLLSPLSITTFYLHCPSPLSISTVHVHCTSLPSISTVHVHRPSLPSTSTAHVHHPSLPSVSTVHVHRPSLPSVSTVHVHHPSLPSVSTVHVHRPSLPSISTVHHHPLSPLSMSTIHHHPLSPLSMSTVHHHPLSPLSMSTVHHHPLSPLSMSTVYHYPLSPLSMSTVHHHPLSPLSMSTAHHHPLSPLSITTLYLQGPSPPSVSTVHVHCSSPPVSTVHVHRPSPPCISTVHVHCPSPPLSPLSMSTVHHHPLSPPSITTLYLH | **none** |
| **ORF5** [152168 - 156070] VTSSAFTLLYNHHHPPFPPSISTVHYHHLSPPSITTHHLHPPSPPSITTLHHHLPSPPFVHPPSPSPPSITTLHHHPPSPPSISTLHHHPPFPRSISTIHHHRPSPPSISTVHYHHLSPPCITTHHLHPPSTLHLHLPFPPSIATIHLHLLSPLTISTLHHPPFPPSISTIHHNHSSLLSITTLHFHPASPPSISTLHLHPPSPPSISTLHHHPPSPPSITNLHRHPPSPPCISTLHHHPPSPPSITTFHLHPSSTLHLHRPSPPFISTLHPPSISILHHHPPFPPSISTIHHNHSSLLSITTLHFHPASPPSITTFHLHPPSPPSIATIHLHHPSPPSITTFHLHPSSTLHLHPPSPPSITTIHLHPPSPPSISTLHLHPPSPPTISTLHLHHPSPPFISAVHLHHPSLPSITTLHFDCPSPLSITTIYLHPPSPLTISTLHPPSISILHHHHPSPPSITIFHLHPSSTLHLHPPSPSSITTIHLHHPSPLSITTLHLHPPSPPTISMLHLHHPSLPFISAVHHHPPFPPCISTLHFRCPSPLSITTIYLHPLSPLTISTLHPPSISILHHHPPFPPSISTIHHNHSSLLSITTLHFHPASPPSISTLHHHHLSPPSITTLHHHPPSPPSITTLYHHHLSPPSIHPPSPSSITTNYFYPPSPPSITTLHLHPPSPPSITNLLHHPPSPPSISTVHHYPPPSITTLHLHPPSPPSITTLHLHPSTPLSMSTIHHHPLSPLSMSTIHHHPLSPLSITTLYLQRPCPLSITTFYLHCPSPPSISTVHHHSLSPLSMSTVHHYPPSPLSTSTVHHYPPPPLPMSTIHHYPLSPLSMSTVRHYPLSPLSMSTIHHYPPSPLSMSTVHHYPLSPPSITTLCLHCPCPPSITTLYLHCPCPPSITTLYLHCPCPPSITTLYLHYPCPPSITTLYLHCPCPLSITTLYLHCPCPPPITTLYLHCPSPPSISRVHHHPLSPLSMSTVHHPRSPLSMSTVHHHPVSPLSMSIVHHPLCLHCPCPLSITTLYLHRPSPPSISTDHHHPLSPLSITTLYLHCPCPPSITTLYLHRLCPPSITTLCPHRPCPLSMSTVYHHPLSPLSMSTIHHHPLSPLSMSTVHHYPLSPLFITTLYLHCPSPPSISTVHHHPLSPLSITTLYLHCPCPLSITTLYLHCPSLPSVSTIHVHCPSPPSISTVHHHPLSPLSMSTVHHHPLSPLSITTLYLHCPSPPSISTIHHYSLSSLSMSTSHHHPLSPLSTSAVHIQYPFIPSITTIHVHCPSPMSIITTHHHHPSPELLPLPKFQVCIH | **none** |
| **ORF6** [152172 - 156173] HQVHSHCCTTITILHFHRPSLLSITTIYPHPPSPPTISTLHLHRLSPPSITTFHLHPSSTLHLHLHHPSPLSITTLHHHPPSPPSITTHHFHAPSPPSITTVHHHPPFPLSITTIYLHPVSPLTISTLHPPSISIFHFHRPSPPFISTFYHHSPSPPSITHHFHPPSPPSITTIHLCCPSPPSISTLHLHPPFPPCISTLHHHPPSPPSITTLHRHPPSPTSIATLHHHPASPPSITTLHLHPPSPPSISTLRPPSISTVHHHHSSPPFIHPPSPSSITTHHFHPPSPPSITTIHLCYPSPPSISTLHLHPPSPPSISILHLHRPSPPFISTIHHHPPSPPSISTLHPPSISILHLHHPSPPFISTLHHHPPSPPSISILHHHPPFPRSISTIHHHHSSPLSISTIHHYHPSPLSISTVHLHCPLPPFISTLHHHSPSPPSIHPPSPSSITTIHHHPPSLSSISTLHPPSISILHLHRPSPPFISTIHHHSPSPPSISILHHHPPFPCSISTIHHYHSSPLSITTLHFHPASPPSISAVHLHCPLPPFISTLYHHSPSPPSIHPPSPSSITTHHFHPPSPPSITTIHLYCPSPPSISTLHLHPPSPPFITTIYLHHPSPPSIITHHLHPPSPPSITTIYLHPPSTLHLHPPSPPTIFTLHLHHPSPPSISTPHHHPPSPTSFTTHHLHPPSPLSITTLHPPSPPSISTLHHRHPSPPSISTLQLHYPCPPSITTLYLHCPCPPSITTLYLHCPSPPSISSVHVHCPSPPSISTVHHHLLSPLSITTLYLHCPCPLYITTLHLHCPRPPSITTLHLHCPCPPSITTLCLHCPCPPSVTTLCLHCPCPPSITTLRLHCPCPPSITTLYLHRPSPPSVSTVHVHHPSPPSISTVHVHRPSPPSISTVHVHRPSPPSISTIHVHRLSLPSISTVHVHCPSPPSISTVHVHRPSPPSISTVHHHPLSPGSITTLCLHCPCPLFITPGLHCPCPPSITTLYLHCPCPLSITPSVSTVHVHCPSPPSISTVHHHPLSPLTITTLYLHCPSPPSISTVRVHRPSPPSISTVCVHRPSPPSVPTVHVHCPCPPSITTLYLHCPCPPSITTLYLHCPCPLSITTLYLHCSSPPSISTVHHHPLSPLSITTLYLHCPSPPFISTVHVHCPSPPSISTVHHYPLSPLSMSTVHHHPLSPLSITTLYLHCPCPLSITTLYLHCPSPPSISTVHHRPLSPPSITTLYLHCPCPPAITTLYLHCPRPLSTSNIHLYHPSPPSMSIAHLQCPSSPPITTIHLQNFYLFPNFKSVFIKQSLPMLLLSATISMKVTTLGTSHKWDCMVRVLL | **none** |
| **ORF7** [158326 - 159489] LSVNPLALGQGAGLLCAPGSARRAGAGPGGEAGGAAETGGHGVCAGCEMSRGGQRGLGGGGRWAWGRGGMAARQASADRTTRGATGAGSPGGRGPALTWFPAGRRGVRRGRAGAGRAGACPGRGVPGAGLSLPQTSGTRARGRRRPLCRGLGPGCHERAGLAGHVLGGGPAQRSAGRAGGREGRDRAGPSWGTSRGPSRGPSRGLRRAGDRARTAGRTRRPSAGRGPGLPSPAPARLWGLTGRGVPAEAGQRRGGWGPPGAPGQAGPRRRGGGPGLRQAGRSRPVSTRPAEGAASLGTRVPAPAPARPWPPPESADSEGAAPSPAGPGRLPPGLVSPPAVPPSVFLLLPSLLISLRRRLPAVPASPGLCAPSTAGLPRPARCPSLPWA | **none** |
| **ORF8** [169110 - 170327] LASASVSPCPSGAVMGAWLWPYDWTTGLDLVRALGHLSSGGVVLGGLVAQKGSAALGVGAPKVWTLAESCVCSCGAGPGVGEGSLRQAPSPPAWLPPPDLGREQRVPDDQRAPSTPTAQRGASLGRAVMRGGLGQQQRVGDVALASWRILLWRQGGHSSVVRGLGATAGVVLGQGMEQGCLCLLGVTSSPGAPEGWLCVSPVPQRGQAACRHQLRPGQGRALGEGPGQPQWGKCERPWPGLLTARPFSPGPSLSVLAPPPVIAPPSQPWPLPLSSASSPSHSPSFSALAPPSQLWLLPQPWPLPLSPGPSPSPGSSPKSGPSPRSGPIPNYRGPQASPEGSPWQGLRPPRIRLLSGRVDPADGKGSTRLGNGAPEMEGAMLGYPVWVWVEPCDPFFCKLGVAGAGR | **none** |
| **ORF9** [176964 - 178025] RVSAQLAAVPVPGQAGDRTLAGSALLPCRGAHSAGLTSHFSVNCLRATCLVRSPVLRLPVALLCPVWGGPEGWGALSTQLRHGVMRLPEIDSCFLSLGLTRWGPLSPPGHKHEDGTQSDSEDPLAKAASAAGVPLEASGEQVRLQRQIKRDPQELLHNQQAFVIEFFDEDTPRKKRSQSFTHSPSGDPKADKRRGPTPADRDRPSVPAPVQAGGRSSGPQRAGSLKREKTEERLGSPSPASRTPARPFGSVGRRSRLAQDFMAQCLRESSPAARPSPEKVPPVLPAPLTPHGTSPVGPPTPPPAPTDPQLTKARKQEEDDSLSDAGTYTIETEAQDTEVEEARKMIDQVQPSGE | **none** |
| **ORF10**0 [178919 - 181573] RGRGPADGATAGVCLPATGCGPPGGAPGTGTDGHPKEGLGRKRAASKPDLGSAGPPWGETGLSSEGHAQRRVGWGFHEEGSGPGVRPTVDLRSWAKRALRSGQGHGCSANLELSSVRLPGLGRQGVALEQVQARQLGSLAAFAGARLLMRQALGGAHAWGWCWLLWTPLLFPGTPCFSPLGLRVETLPPSSPQGLPVPGSPGGQKWVSRWASLADSYSDPGLTGKWLQCCGGVGPGRGGPQATDHGHRLPLPERGGPADQRPSNRLPLCSTEDGLGRRGGEPEGSLPVRMRRRLPQLPSERADSPAGPESSRRSGPGPPELDSEQPSRLFGQEELDPDSLSDASGSDGGRGPEPGVEPQDSRRRSPQEGPTWSRGRRSPRAPGEPTPASFFIGDQNGDAVLSRKPLAAPGDGEGLGQTAQPSPPARDGVYVSANGRMVIQLRPGRSPEPDGPAPAFLRQESFTKEPASGPPAPGKPPHISSHPLLQDLAATRAARMDFHSQDTHLILKETETALAALEARLLSNSVDAECEGGSTPRPPEDALSGDSDVDTASTVSLRSGKSGPSPTTPQPLRAQKEMSPSPPAAQDPGGTALVSAREQSSERQHHPLGPTDMGRGEPVRRSAIRRGHRPRGSLDWPSEERGPVLAHLPSSDVMASNHETPEATGAGRLGSRRKPAAPPPSPAAREEQSRSSASSQKGPQALTRSNSLSTPRPTRASRLRRARLGDASDTEAADGERGSLGNPEPVGRPAAEQAKKLSRLDILAMPRKRAGSFTGTSDPEAAPARTSFSGRSVELCCASRKPTMAEARAVSRKAANTATTTGPRQPFSRARSGSARYTSSECQGGWEARAKTGLPVGLQHLGCQLSAGRGPLVLAREEPLFPKLG | **none** |
| **ORF11** [220932 - 222149] PLPPPWAGGLLCITLPSPLSTLVPPQWQPRSGKSRRSLSQHWRGHRGPARSQAPPCLVTLRVLPHPVAGPGSAGCAVAGLRGSYLPPVASAPSSHLGPGAAQGRAQVLGAWLQPSLGAPGSRGQAAEGLLPVSEPMEACGGRSQFGAAACVSALGSAREIRGALYQLLQEGAGHQGGPRPYELEWGLGTGQACGAFCSKQRLPPGGSDRLENTACAEAHPLSPSTPGGNVTVPCRPSSFPPTLLSLRLGSWNPRTAPEEPVGPSCTHPLETGAYLPLAQPFPSRPALATTKGFHTTRPPQTPDSTALLYSARLQEQLLPVGSASGLSGARTPQSSLLWTRQPLSGSFPNCSWVSPPQQHAGLRPTLPSLFCCLYLKPLPCTLGLGSSRGPPEQRLLWLGSGMGQVM | **none** |
| **ORF12** [234986 - 236050] RPFKTRRAQFWSLKLTVFPSYILVGQGQVPLKPRTIQHGSWGLDIRLHAGQRHLHIGGHHLCLWAFQVQLGALDVHLGALEVHFGHLETGHLHLGQVPFEAGYLGHVAFWKFQVHLASLDLQVGGRGLNAEVSGLEVPLHGGNAHVGLHLWRRHIHRDLDGLAWGQHPKGWHLELGHFELAVFGSHVLVSQGQVPLQPRTVQLGSWGLDVHLHAGQRHVQVGGRHLHLGAFQVQLGDINVYLGTLEVHFGYLETGHLQLRQVPFEAGSLMHRALWEFHILLASLDLQISGRGLNAEVSGLQVPLHGGEAHVSLHLQRRHIQRGLDGLAWGRHPERRHLELGHFELAVFGSHVLVG | **none** |
| **ORF13** [238652 - 240442] VQLGALDVHLGALEVHFGHLETGHLHLGQVPFEASSLGHVALRELHVHLASLDLQVHRRELNAEVSGLEVPLHGGEAHVGLRLRRRHIHRGLDGLAWGRRPVRRHLEFGHFELAVFGSHILVGQGQVPLQPCTIQLGSWGLDVHLHAGLRHLHVGGRHIHLRAFQVQLGALDVYLGALAIYFGHLETGHLQLGQVPFEASSLENVALWELHVHLASLDLQLGRGGLNADVSVLQVPLHGGEAQVGLHLWRRHIHQDLNGLAWGRHPERRHLELGHFELAVFGSHILVGQGQVPLQPCTIQLGSWGLDIHLHAGLRHLHVGGRHVRLRAFQVQLGALDIYLGSLAIYFGHLETGHLQLGQVPFEASSLGNVALWELQVHLASLDLQLGRGGLNADVSGLQVPLHGGEAHFGLHLRRRHIHRDLNGLAWGRHPERRHLELGHFELAVFGSHILVGQGQVTLQPCTIQLCSRGLDVHLHAGQRHLHVGGRHLRLGAFQVQLGALDVYLGALEIHFGHLETGHLHLGQVPFEAGSLGNVALWEFHVHLASLDLQLGGGGLNADVSGLKIPLHGGEAPVSFHLQLRHIHRGLDGLAWGRHPE | **none** |
| **ORF14** [242612 - 243907] VQFGALDVHLGALEVHFGHLQTGHLHLGQVPFEASSLGHVALRELHIHLASLDLQVGRRGLNAEVSGLQVPLHGGETHVGLHLGCRHIHRGLDGPPWGRYPERRHLEFGHFELAVFGSHVLVGQGQVSLQPRTIQLSLLGLDIHLHAGQRHLDIGGCHFRLGAFQVQLGALNICLGALEVHFGHLETGHIHLGQVPFEAGSLGHLALRELHVHLASLDHQVCRRERNAEVRGLEVPLHGGEAHFGLHLRRRHIHRGLDGLAWGRHPERRHLELGHFELAVFGSHILVGQGQVTLQPHTVQLGSWSLDVHLHAGQRHLHVGGHHLRLGAFQVQLGALDVHLGALEGHFGHLQTGHLHLGEVPFEAGSLGKGALREFHVHLASLDLQVGGRGLNAEVSGLQVPLHGGEAHISFHLRLRHIHRGLDGLAWGRHPK | **none** |
| **ORF15** [245384 - 246382] RPERWHLELGHFEPAVFGSHILVGQGQFPLQPRTVQLGSRGLDVHLHAGQRHLHIRGCDFRLGAFQVQLGALDVHLGALEGHFGHLETGHLHLGQVPFKASSLGQVALRELHIHLVSLDLQVSRSRLNAEVSGLQVPLHGGETQVGLHLGCRHIHQGLDGLPWGRYPERRHLELGHFELAVFGSHVLVGQGQVPLQPRTIQLGLLGLDIHLHAGQRHLAIGGCHFRLGAFQVQLGALNIYLGALEVHFGHLETGHLHFGQVHFGAGSLGHRALRKFHIHLASLDLQVSGRGLNAEVSGLEVPLHGGEAHVGLHLRRRHIHRVLHGLAWGRHPE | **none** |
| **ORF16** [246261 - 247679] MLRSVALRSPCMEERLTSASTFGADTSTESSMDLPGADTPNDGILNLGILNLLSLAVTSLSARDRSPSSRAPSSFALGAWTSTSMLDRDIFTSGAVTSALGPFRSSLGPLRSTLGIFKLGLCTLGRCPLRLAPSGRGPSGSFTSTWPSWTSRSAEGVWTLRSVVLRSPCMERRLTSASTFGADTSTEASMDLPGADAPNNGILNLGILNLLSLVATSFSVRICSFFKRFSSCISCIFVVFVIESLSSLSFNPSSVRLCSLLSAVAVASPSLPCSVSSVAFSLPVFVCLLGDHPKEGILNLGILICPSVSSLPLSCSSVILVLCSPSSPSPSSPCASACVVGSLPGIHPAPSTSSPFRPVPAFEDASCLFPRCGVLRRLSLSGSFSVENANFGVFKSCTRTLISGGPICVPPSVVSLKDSLAIPISRLCSPSPSPSRLIPGVGWALGFLWATAVSCACPSRVFPWSLAALSPPS | **none** |
| **ORF17** [258740 - 259873] SQLLGRLRQENHLNPEGRGCSEPRSCHCTPASATKAKLCLKKKKKKKKRKEKKKSNPLFKTVPQKGLQSQPHSELYSKYSKKKQYQSYANSGALAWAPGPPSCSDKEGLRTKAPIPAPDPVAGRTLIGKEHVPILWGGVLASGTSCSLSPAVGVLALGTVPRRQMGRPSPGSGLGLAAPLASVLCGPEQAHPLSELSFPHGYVDHPACPDCGLLGYRHCSRRGTPRAMPAHPKSTSACAGPCHSLTASRAFPCPSARWLTAQVPSGSALRPGRAGGWCLLEEVLSSEGREGQEVRAGRAGSVPGGINTHPHVIPAPPPSYCSGGQAQTPGLHNPPAEPQPPFPPPAKPALPTVSAQELYPWQPHNTISSTGPFSLTRQ | **none** |
| **ORF18** [278909 - 280480] TREAELAVSRDRATALQPGRQSETPSQKKKKKKKKKEPLGRRTEHGQKARAARRPGLGQCGSPIPPAAEAARGREGGSGAGPERRGPALASPRMRADATATDAGSGAGARRCQATGRRRQEGTAGCRFLSPWSRAAPPRRRPRWVRGLSGAVGQALSRPPRALSRPRRSRAALRAACERDSREAPGRRTHSFPLGTPAGARLSLLWPAWASQAPSRLSPEQEPHLGPPLSHLPWVWYAPLCRTPRPPAWEEARTPGRPGHSCGLCQFHSSTWALSTVSHWAARGTPAASPGPLQGRGRLGSPGRPAPRRACLLSSCPSGLCWPGGSKMQGRRELGGEPLSDLQEEAASASLRVAPERLSDDSLEWRRTCPDLLLSDGKASISMPREGGSTCTARCPDPGEHSSTWGEFEGFRESSAKSGQFSQSLELLEGPTEPQPPRTTSAPKECSSHQPCQGGPWVTGTSAVPPSEVFLLCCGHHVEWDVYTPRGLQPSNAVACWPGRGLTAPERLLLSWPSSLAMWLAVPS | **none** |
| **ORF19** [376117 - 377748] ILVNNLLYLFLHTSLHLPIHLSIHPSTPLSTPSPTIYPSIHLYPSIPLSIHPSPINPHPPLQPSIYPFLSILPSIHLSIPLSTPPPIPQASIHPPIHPSLSILLSTHPLLSTPPPIHPSLSILLSTHPLLSPPLHPPLHHPSIHPSLSILLSTHSLLSPPLRPPLHHPSIHPSIHLYPSFYPPTHSSIHPSTQPSILHPPISLSTHPSIPLSALPLLYSPLHSPLHPSTHHSIHPTASLSTPLPTPPSSIHPSIHPSIPLSALPLLYPPLHPPLHPSTHHSMHPSTSLSTPPPTPPSSIHHLSIHTSIYPSICPSTPLSTPPPTPPSIHPPLYPPIHFSIHSSTHPPGIHPSTQPTISIHHSIHPSTPLSTHPPTPPSSLHPSIPLSTPPPSIHPSLYPPIHLSLYLPFHSSIHHPPIHLYPFIPVSTHPLLYSSIHPSFYPFLYPPLHPSIYPPTHPSIHLSIPLSTPIHPSILPPIHPPIPLSSLPLLYPPLHTSSSIHPFLYPFVHLFIHPYIHPSIHPLTTVSIHLSSLCFPYKPHVLGG | **none** |
| **ORF20** [376124 - 377830] LTIYCTCSFTHLCIYPSISLYTLPLLYPPLHPPSTHPSISILRSLYPSIHPPSTPIPPSSHPSIHFYPSSPLSTYPFLYPPLHPSPRHPSIHPSIHLYPSFYPPIHSSPPLHPSIHLYPSFYPPIPSSLHPSTHPSIIHPSTHLYPSFYPPIHSSLHPSAHPSIIHPSTHPSISIHPSIHPPTPLSTPPPNPPSSIHPSLYPHIHQSLYLPFHSSIHPSTHPSIHPPTTLSTQPLLYPPLYPPLHPPSTRLSTHLSLYLPFHSSIHPSTHPSIHPPTTLCTHPLLYPPLHPPLHLPSTISLSTHPSIPLSALPLLYPPLHPPLHPSTHHSIHPSTSLSTPPPTPQASIHPPNQPSPSTTLSTHPLLYPPIHPPLHPPSTHPFLYPPLHPPSTHLSIHPSIYLSICPSTPLFTIHPSISIHSSLYPPTHSSIHPSIHPSIHSCIHLSIHPFIHPPIPLSIFPFLYLPPSIHPSFHPSTHPSLYPAFHSSIHPSIHPALSIHSSIHLSIFSYIHISIPLSIHSPLYPSTSPPFVFPTSPTSWGADSNWGCCFQASCLSELETSRERPDSQT | **none** |
| **ORF21** [376128 - 377996] QFTVLVPSHISASTHPSLYTPFHSSIHPFTHHLPIHPSLSFDPSIHPSIPHQPPSPPPAIHLSISIHPPLYPPIHSSIHPSTHPPGIHPSTHPSISIHPSIHPSTPLHPSTHPSISIHPSIHPSPPLSTPPPTPPSSIHPPISIHPSIHPFTPLSTPPPTPPSSIHPPIHPSLSILLSTHPLLYPPLHPTLHPPSTHLSIHTSINPSICPSTPLFTPPLTPPSIHPPLYPPNRFSIHPSTHPSILHPPVYPPIYPSICPSTPLSTPPPTPPSIHPPLYAPIHFSIHPSTHPSIFHPPSLYPHIHLSLYLPFHSSIHPSTHPSIHPPTTLSTHPLLYPLLHPPPRHPSIHPTNHLHPPLYPPIHSSIHPSTHPSILPPPIHSSIHPSTLHPPISLSTHPSISLSALPLLYSPSTHPSLSIHPCIHPPTPLFIHPSILLSIPVSTSPSIHLSTHPSLYPSFHSSIYPHPSIHPSTHPPTHPSIQPSTPLSTPPYIQLYPSIPLSICPSFHTSIYPSLYPSTHHCIHPPLLPLFSLQAPRPGGLTPTGGAASKPHALVNWRPAEKGLTARPKGCHRLLVQPTPTSIHHRLRTGMSSSPWAQGSSQEPAEARLFFGLCRVCTPRPAE | **none** |
| **ORF22** [380329 - 381375] DVFLSSPNHVPSPAGPGAALPPGGCGCAAPDRPALQGIRPPVPPPAPGSAPRAAEVETAARRWCYPQPRLLRSGPLLSWVQSALRGGPSARRPRTRLPRPQSPPRHQTHCRPGAFVSQAGKWACCAHGRREDGGARPGGLEDSRGLRGPEVLLSPSDPQDSLPQFPPWSRTETHSSPPLGPEFPVPSPQAGNHPVPSAPPQPPAQSIPARTVPFNLRPRPGASLALPARRGRLRAGGQPRGGGIRAHLHAAAGWSAPGPVEAAGGAAAGRAGRLRLRGPEEPPQRLERAQAGWGAGAGRAGGGGRAGPALGFLGPSTHARLSGAPARFPGRSPLASPLALLFAQSPRGG | **none** |
| **ORF23** [380321 - 381493] ETETCFSAPPTTSPAPPVPELPCRLVAAAALRPTGPRSRASDLQFPRPRPGVRREQQRSRQQPGGGVTRNLGSCARGRFSPGCSQLCAGDPQHAVHGHGSLGHSLLPAIRRTADLGPLFPRQGNGRAAPTGAGRTGEPALGAWRIRVASAVQKYSSPLAIPRILFPSSLPGLEPRLTAPHPSVPSSLSPPPRPGTTQSPPRLPNPQPRAFLPAPYPSISAPGQEPPWPFPPGGAGSGQAGSLAAAASAPTFMPPQAGARLGLSRPLAEQRRGGRGGCVCAARRSPRRGWSEPRQAGALARGELAGAGELAQRWVSWVPARTRALVGRRPASQADRPWPRLWLFYLHNHPEVGDAIVPTFQIRKSRHNWNSYPGSRHRPSSGRGLRRNKHHS | **none** |
| **ORF24** [405403 - 406509] PPYLPPGRGGWPGGGLTPPSPSRTGWLARLRGSSLPSRGGRAEAPLTSRTGRLAGRGADPPTSLPDGTAGRAGGLTPPPPTSLPDGVAAGRRRSSLPRWGGCRAERLLTSQTGWLPGRGSPHFSDGAAGQRRSSPPRRGLGRAEALLTSQMGRRGRGAPHISDDGRPGRDAPHFLDVMAAGKRRSSLPRWDGGRAETLLTFQTGQPGRGAPHIPDDGRPGRDTPHFPDGVAAGQRLQSRHFGRPRQAAAPCPRAPRGPSAPPAAASRAALAGAAAKTETAPLPAELHPPGGRAAAAAVGRGSGSASYLQLGPAGVSAATVLAPHCPGPQRSRANHQPRPPWPDGLPKPPTPARGAFDPSGASGAATSSA | **none** |
| **ORF25** [438568 - 439695] HLHPWTGAVGTRGTHPTARPGEHSKDAKGLGGQLPTRLCPIPGGRSDGAHPPILRADPAPPAGQWGKERQGGVQWVSSSSEPLLGDRGPAPSTQQAKVAPRPGQNCPRSYSPRAGARQPWRRQSRPSGQLDKQAPPAGCSPSLLCHLHPAPGPMPLSSDPTGPLSVTKSDSAQPTRASLPCGARLSPCHEQMPACGLCKLRCHQAPHLGHVVTVEMQGEAGTVIVQVAGAWQAGGARWLFCAALPPGLTGWSQTLAWGLLQAPLPQWALGAQGLGLAGQQTRLPSAPHLGRHAQEGQAQAHRRVRCRHTDENPQAGTGPMPRRDTAQVCEPQGPAHPLPRAPREAGQARHQKGQAEQGRTQTRPVFPTAHRSMGPG | **none** |
| **ORF26** [450659 - 452182] SEPSPGCSPCTASPLLPRPSHELLPWPVGASAFTHGSHPVTPAGPCRAPASGRGPPRLHAPVQTSRGGHKARDTLKGVPSTKSLPWAPKEHTQEENNIGKARTGEESACTALQVPNVAELPGSQSKKGNSTHDPQGKARLCPKQTEELGPATGTLKLRGAATWPRAHSPFGVVSLSQSQASTMRVKERCRHGEAGQVPEATPACLVQGGTRPRAQTLPLGLPGSGPSSPARDPPPSSSSGHILCRPKNDPAQGRETAPPGHAGSGAWAPYRWGARGPACPELAQVSTEHRKSDRGERWAKGSLAGVATDRGPMGSRDPSVSCGTPLGASLGVTTGENVGPHHHAGGLHQVHGVAKYRAPSQRPQQAGAQVLQAYSTQANPHLPPPSHRTGGRRGPCGTVGETEAKRSWDCPAQPQTMATMPPLPPGHLALVPQPWRQAMLSAKQRDHRAGPLLRLVQPTETVTHRHAQTGGTPGTCPVLPSTGYPHSTASQPSLPRNPPVYPWCALDP | **none** |
| **ORF27** [452809 - 453975] PRRADNRPSIPPLKLPLNPFSHIRHGPARPRGPVPAHLQGSLRSPGPANLCLQAPDNPQSSIHMPPKAPSTPPAELCLHPPELHPHPQRTQSTSPNGVPSTPPNRLHHLPRAPSTPRNMLHPHPPRAPSTPPQGSIHTPPEFHPHTPTCSVHTPPQSSVHTPQHAPSTPPRAPSIPPRAPSTPPEFHPHPPTCSIHTPQHAPSTPPNRAPSTPPPELCPPPPTCSIHTPPGLHPYPPRAPSTPPNMLHPYPPRAPSTPPRTLSTPPPGLCPHPPRALSTNHPRQLHPHPPGLHPHPHQDSVHALSAQSTLGVPKAPAHPHPPTPAYQPICLEHHPRSCTWPSPSPPPVTPGAPSRFPGVWHLAPSFVPSFLRSHQVYSRHASATKDGNL | **none** |
| **ORF28** [452850 - 454085] APLKPVFPHPPWPRQATGPSPRPSPGLPEVTRARKPLPTGARQSPEFHPHASQGSVHTPRRALFTPPRAPSTPPKNSIHIPQRGSIHTPQQAPSPPQGSIHTPQHAPSTPPQSSIHTPPGLHPHPPRVPSTHPNMLRPHPPPELCPHPPTCSIHTPQGSIHTPQSSIHTPRVPSTPPNMLHPHTPTCSIHTPQQGSVHTPPRALSTPPNMLHPHPPRAPSIPPQSSIHTPQHAPSIPPQGSIHTPQDSVHTTPRTVSTPPKGSVHKPPPSAPSTPPRAPSTPPPGLCPRPLSSEHPWGSQGSCPPPPTHPSLSTHLPGAPPSQLHLAKPFPTPGNTWSTQPLPRRLAFGSFLRSFLPSFTSSVQQACLCHQGRQPIASGARGKQQGAGAVVPIRPGQRVTVAHACNPNTLRG | **none** |
| **ORF29** [455739 - 457085] PTPTAQADPQPNFISHHLIASSPARTLKAKSKPLLLSPGRGNSSGTSQRTGRQDNRCSTPGQGTSKPRVPQVQGTPKPRVSHSPGHCMAQHTPRPRAPQFQGTPKPRVPQSPKALHGPAHPKVQGTLKPRTPQVQGTQSTEYPPAQGTLDPVHPTALCTPWPSVPTVQGTPKLRALQVQGTPSPGHCMAQHTSWPRVLQMPSTLSPRHPTAHCTPIYSIPKLRQPKSRALHGQGQHSLPAAPLTAPPGPGPCTVQANTTQPPRPAAPPTPRLGFFSSASRISLPPPTEHSAWCRRQELQAQIHFLKPELLRGPGLCHHCHPRQGRRGGGKHHPQGSGPQGRSRRSRPKPIPRRNTHTHAPHQRPARALPGCTPTPQHTPSRSQVPHREDGGSQGRLAPATKPRVQLMPATPCSGLGCGDRNPAQACGLGTGTPLRPVVWGQGLRSGLWS | **none** |
| **ORF30** [460741 - 461754] VPRRPPVPPRHPGNCRAGAGWPRARGGSVRACPRPAPARAAHMPPRPARDRGASPRLPTQTDTRRATQAHAAHRRARGTPPPRPRRDPAPGGPGAPARPRGQARTKSPEHAPPAGPAPPGRARLARRPGRRCWGSRSARGRGAEREGRAGARGMERTYRPGELEGDDDEARVLVAAGPGPRPRSVPRSARRRQVEGVAAQHGRGAVAVAAGPRGRHLGLVLLKAHAHVRVALVVAAAPRARCPAVAVTAGAAQQLPVHVPQRAQLQLEIAHGPRRLKIRQRESGGRRGPGSAGRAPGVGEQAPPPRPHPAAPARSPSRRAQPRGLPNHGGPRQCPPGP | **pol protein, partial [HIV1]**  **Sequence ID: AJD23612.1**  **E value: 3.3** |
| **ORF31** [469441 - 470469] RPWGSGWASYQHLVLGRQPHPSWQPSRGKSWCLEFGGSGPKAQALVPVPQESWSHPFSSPGHSFSLIPKPPSENRAVSHPHPQRPVLILQEVEAPVSSSCHPACLTQLGAAGWGHGAETLQREPQEPRFWPGLGAEAHSVQPSPNPMLENKEESILLAHSQGRGPHSLPSPESHHRPEGLVPLTSMWRPGADQRGPSGSHTPPKPPCKPLPPARAQAGPRGRHTAQLSPPLSRAGLAVAPEARPGLQLLGTLGSGAPPHPHTQLRGKKPDCAHSHVVATSRSPTQPSPLPGPQLPLEPPPTSVFRPSGPQGPEGDPADPLPTPAGLTWPELLHQHQAPPRRVE | **none** |
| **ORF32** [485561 - 486835] DVGGVPAAPDLCRRCPGSLGSTSAHKAFSTLTPHTPRRTLRPHADPTHPRRTLGLHADPTHPRRTLGLHADPTHPRRTLGLHADPTHPHRTLGLHADPTHPRRTLGLHADPTHPRRTLGLHADPTHPRRTLGLHADPTHPRRTLGLHADPTHPRRTLGLHADPTHPRRTLGLHADPTHPRRTLGLHADPTHPRRTLGLHADPTHPRRTLGLHADPTHPRRTLGLHADPTHPRRTLGLHADPTHPRRTLGLHLLLGPAAFPNHGRVSPRQSSQTGPQQGWGLQALPGGRGWAGLTVLGNALWSLPGKRREGVASQRRTHLPSSPQPTLYMGTPETTARHRIDLPHSGPPSPSFDRPPALLLPAPALWTCVTWGWPLDRGPHSWPLGAPGSRRETAQPRAPAPSEPQPPLQSKPTRKALAQGTMPFV | **none** |
| **ORF33** [499425 - 500723] APTQHEAASCRGCWPHSLLFPCSSALDRGCWVIRCQLCWTWELLPLALGVSCKAPALRKAWRQPAPLQGLEEAKVRGTDARPVPPLASVVHTHTAGPNEHGSRLWAGMAAPLQLGYRDCGQAWQPLYSWGTGVWAGMAAPLQLGYRDCGEAWQPLYSWGAGVWAGIAAPLQLGYGIVGRHGSPSTVGVRDCGQAWQPLYSWGTGLWADIAAPLQLGYRDCGEAWQPLYSWGAGVWAGIAAPLQLGYGIVGRHGSPSTVGVQGCGQAWQPLYSWGTGCGQHGSPSTVGVRAVGRHGSPSTVGVQGLWAGMAAPLQLGYKIVGRHGSPSTVGVQDCGQAWQPPAVGVQGCGAWPSLFSGSGSEVTGQTSLRTWQGAQRRQRVRSRAPPSNSGSRGFLQGALPTPAPWAAKKEPSRHRTAGPRQWSSGPAVVEPRA | **none** |
| **ORF34** [508358 - 509677] KAAPMGCKGNRDKPFCPQVDPLSPAPLELPELPRPEWGWECGGSPALRSSCFRLRTLNIPESDHGHVGGKTDGFLKQEAVHEHADVCAHRLTDAHTLHTVERLKTHTHAPRTQRNTVYTDTCMHTRVHRDKDLHTTICRHTGTHVACTHTACTERHIGVHTCICTQKLTHMHTHYVQSQIQAHTVCRHQNTCTHTRTECTNAHTYCVHTHCMHRHRHTHMHTYTAGTQTQAHTHCIHRNRHTHTAYTDTGTHTLHTQAHTHMHTNTACTQAHTHMHTHTLHTQTQAHTHAHTLHTHTCTHTAYTGTHTCTHTAYTDTGTHTHAHTRCRHTDTGTHTLHTQTGTHTLHTQTQAHTHMHTHCIHRRTHTCTHTLQAYRHRHTHCVHRHRHTHTHAHKYCMRTDTGTHCMHTDTGIHALHVHVYTHRETHMHTRALSGPAGQM | **none** |
| **ORF35** [508830 - 509852] ACTPAFAHKNLHTCTHTMCSHRYRHTLCADTRTHAHTHVLSAPTHTRTVCIRTACTDTGTHTCTHTLQAHRHRRTHTAYTETGTHTLHTQTQAHTHCIHRHTHTCTQILHAHRHTHTCTHTHCIHRHRHIHMHTHCTHTHAHTLHTQAHTHAHTLHTQTQAHTHMHTHAAGIQTQAHTHCIRRQAHTHCIHRHRHTHTCTHTAYTGAHTHAHTRCRHTDTGTHTAYTDTGTHTHMHTNTACAQTQAHTACTQIQAYTHCMYMYTHTERHTCTPGPCLGQLVRCDRGGCEPCHQAPESPNWDRTPTSSADRGWLGRQRTRRLKPQIGPGSAQAQGNRATDLP | **none** |
| **ORF36** [522935 - 530323] GVRALPTWGSNSGAGVVDQATATSTHQHQGQSESLSDSCDLGGHRGPTRTVAQLGAGDSLAAPWHPQEQQPSISGERGHTTVRVVAQLGAGDSLAALWHPQEQQPSISGERGHTTVRVVAQLGAGDSLAAPWHPQEQQPSISGERGHTTVRVVAQLGAGDSLAAPWHPQEQQPSISGERGHTTVRVVAQLGAGDSLAAPWHPQEQQPSISGERGHTTVRVVAQLGAGDSLAALWHPQEQQPSISGERGHTTVRVVAQLGAGDSLAALWHPQEQQPSISGERGHTTVRVVAQLGAGDSLAAPWHPQEQQPSISGERGHTTVRVVAQLGAGDSLAAPWHPQEQQPSISGERGHTTVRVVAQLGAGDSLAAPWHPQEQQPSISGERGHTTVRVVAQLGAGDSLAAPWHPQEQQPSISGERGHTTVRVVAQLGAGDSLAAPWHPQEQQPSISGERGHTTVRVVAQLGAGDSLAAPWHPQEQQPSISGERGHTTVRVVAQLGAGDSLAAPWHPQEQQPSISGERGHTTVRVVAQLGAGDSLAAPWHPQEQQPSISGERGHTTVRVVAQLGAGDSLAAPWHPQEQQPSISGERGHTTVRVVAQLGAGDSLAAPWHPQEQQPSISGERGHTTVRVVAQLGAGDSLAAPWHPQEQQPSISGERGHTTVRVVAQLGAGDSLAAPWHPQEQQPSISGERGHTTVRVVAQLGAGDSLAALWHPQEQQPSISGERGHTTVRVVAQLGAGDSLAAPWHPQEQQPSISGERGHTTVRVVAQLGAGDSLAAPWHPQEQQPSISGERGHTTVRVVAQLGAGDSLAAPWHPQEQQPSISGERGHTTVRVVAQLGAGDSLTALWHPQEQQPSISGERGHTTIRVVAQLGAGDSLAALWHPQEQQPSISGERGHTTVRVVAQLGAGDSLAALWHPQEQQPSISGERGHTTVRVVAQLGAGDSLAALWHPQEQQPSISGERGHTTVRVVAQLGAGDSLTAPWHPQEQQPSISGERGHTTVRVVAQLGAGDSLAALWHPQEQQPSISGERGHTTVRVVAQLGAGDSLAALWHPQEQQPSISGERGHTTVRVVAQLGAGDSLTALWHPQEQQPSISGERGHTTVRVVAQLGAGDSLAALWHPQEQQPSISGERGHTTVRVVAQLGAGDSLTALWHPQEQQPSISGERGHTTVRVVAQLGAGDSLTALWHPQEQQPSISGERGHTTVRVVAQLGAGDSLAALWHPQEQQPSISGERGHTTVRVVAQLGAGDSLAAPWHPQEQQPSISGERGHTTVRVVAQLGAGDSLAAPWHPQEQQPSISGERGHTTVRVVAQLGAGDSLAAPWHPQEQQPSISGERGHTTVRVVAQLGAGDSLAALWHPQEQQPSISGERGHTTVRVVAQLGAGDSLAALWHPQEQQPSISGERGHTTVRVVAQLGAGDSLAAPWHPQEQQPSISGERGHTTVRVVAQLGAGDSLAAPWHPQEQQPSISGERGHTTVRVVAQLGAGDSLAAPWHPQEQQPSISGERGHTTVRVVAQLGAGDSLAALWHPQEQQPSISGERGHTTVRVVAQLGAGDSLAAPWHPQEQQPSISGERGHTTVRVVAQLGAGDSLAALWHPQEQQPSISGERGHTTVRVVAQLGAGDSLTALWHPQEQQPSISGERGHTTVRVVAQLGAGDSLAAPWHPQEQQPSISGERGHTTVRVVAQLGAGDSLAAPWHPQEQQPSISGERGHTTVRVVAQLGAGDSLTALWHPQEQQPSISGERGHTTVRVVAQLGAGDSLAALWHPQEQQPSISGERGHTTVRVVAQLGAGDSLTALWHPQEQQPSISGERGHTTIRVVAQLGAGDSLAALWHPQEQQPSISGERGHTTVRVVAQLGAGDSLAALWHPQEQQPSISGERGHTTVRVVAQLGAGDSLAALWHPQEQQPSISGERGHTTVRVVAQLGAGDSLAALWHPQEQQPSISGERGHTTVRVVAQLGAGDSLTALWHPQEQQPSISGERGHTTIRVVAQLGAGDSLAALWHPQEQQPSISGERGHTTVRVVAQLGAGDSLAALWHPQEQQPSISGERGHTTVRVVAQLGAGDSLTALWHPQEQQPSISGERGHTTIRVVAQLGAGDSLAALWHPQEQQPSISGERGHTTVRVVAQLGAGDSLAALWHPQEQQPSISGERGHTTVRVVAQLGAGDSLAALWHPQEQQPSISGERGHTTVRVVAQLGAGDSLTALWHPQEQQPSISGERGHTTVRVVAQLGAGDSLAAPWHPQEQQPSISGERGHTTVRVVAQLGAGDSLAAPWHPQEQQPSISGERGHTTVRVVAQLGAGDSLTALWHPQEQQPSISGERGHTTVRVVAQLGAGDSLAALWHPQEQQPSISGERGHTTVRVVAQLGAGDSLTALWHPQEPQPSISGERGHTTVRVVAQLGAGDSLTALWHPQEPQPSISGEKGHTTVRVVAQLGAGDSLAALWHPQEHNLASQEKKATPLSA | **none** |
| **ORF37** [530188 - 534561] HLRRKRPHHCPRSRPAGGGGQPGSPVAPSGAQPSISGEKGHTTVRVVAQLGAGDSLAALWHPQERQPSISGERGHTTVRVVAQLGAGDSLAALWHPQEQQPSISGERGHTTVRVVAQLGAGDSLAALWHPQEQQPSISGERGHTTVRVVAQLGAGDSLAALWHPQEQQPSISGERGHTTVRVVAQLGAGDSLAAPWHPQEQQPSISGERGHTTVRVVAQLGAGDSLTALWHPQEQQPSISGERGHTTVRVVAQLGAGDSLTALWHPQEQQPSISGERGHTTVRVVAQLGAGDSLAALWHPQEQQPSISGERGHTTVRVVAQLGAGDSLAALWHPQEQQPSISGERGHTTVRVVAQLGAGDSLTALWHPQEQQPSISGERGHTTIRVVAQLGAGDSLAALWHPQEQQPSISGERGHTTVRVVAQLGAGDSLAALWHPQEQQPSISGERGHTTVRVVAQLGAGDSLAAPWHPQEQQPSISGERGHTTVRVVAQLGAGDSLAALWHPQEQQPSISGERGHTTVRVVAQLGAGDSLAALWHPQEQQPSISGERGHTTIRVVAQLGAGDSLAALWHPQEQQPSISGERGHTTIRVVAQLGAGDSLAALWHPQEQQPSISGERGHTTVRVVAQLGAGDSLAALWHPQEQQPSISGERGHTTVRVVAQLGAGDSLAALWHPQEQQPSISGERGHTTVRVVAQLGAGDSLAALWHPQEQQPSISGERGHTTIRVVAQLGAGDSLAALWHPQEQQPSISGERGHTTVRVVAQLGAGDSLAAPWHPQEQQPSISGERGHTTVRVVAQLGAGDSLAALWHPQEQQPSISGERGHTTVRVVAQLGAGDSLAALWHPQEQQPSISGERGHTTVRVVAQLGAGDSLAAPWHPQEQQPSISGERGHTTVRVVAQLGAGDSLTALWHPQEQQPSISGERGHTTVRVVAQLGAGDSLTALWHPQEQQPSISGERGHTTVRVVAQLGAGDSLAALWHPQEQQPSISGERGHTTVRVVAQLGAGDSLAALWHPQEQQPSISGERGHTTVRVVAQLGAGDSLAALWHPQEQQPSISGERGHTTVRVVAQLGAGDSLAALWHPQEQQPSISGERGHTTVRVVAQLGAGDSLAALWHPQEQQPSISGERGHTTVRVVAQLGAGDSLAALWHPQEQQPSISGERGHTTVRVVAQLGAGDSLAALWHPQEQQPSISGERGHTTVRVVAQLGAGDSLTALWHPQEQQPSISGERGHTTVRVVAQLGAGDSLAALWHPQEQQPSISGERGHTTIRVVAQLGAGDSLAALWHPQEQQPSISGERGHTTVRVVAQLGAGDSLTALWHPQEQQPSISGERGHTTIRVVAQLGAGDSLAALWHPQEQQPSISGERGHTTIRVVAQLGAGDSLAALWHPQEQQPSISGERGHTTVRVRSRAPRDHGQGCPCSRTPEVGNLGSRPLCPESSAPYARTRRGQRGSQEVLEG | **none** |
| **ORF38** [542889 - 544127] NSHSRRGARSLSDPLKVSPLYWGGTGGSVLGGLRRLLKGSTQELMDSSPRPSPAMGAWEPAPDAGPASPRYMYSDEIDLEADTVLATLYAAKKYIVPALAKACVNFLETSLEAKNACVLLSQSRLFEEPELTQRCWEVIDAQAEMALRSEGFCEIDRQTLEIIVTREALNTKEAVVFEAVLNWAEAECKRQGLPITPRNKRHVLGRALYLVRIPTMTLEEFANGAAQSDILTLEETHSIFLWYTATNKPRLDFPLTKRKGLAPQRCHRFQSSAYRSNQWRYRGRCDSIQFAVDRRVFIAGLGLYGSSSGKAEYSVKIELKRLGVVLAQNLTKFMSDGSSNTFPVWFEHPVQVEQDTFYTASAVLDGSELSYFGQEGMTEVQCGKVAFQFQCSSDSTNGTGVQGGQIPELIFYA | **none** |
| **ORF39** [564745 - 566001] WAHTAVRFLNVEIRGKELKPRSCTARARSQRATELPSENAGGKATAGAQAGRQTCGRARHAHQTQGREGDHLQRGAARKRSLSQGAIAGGTSQDEEGVDQEAGSPQRAGKRCRCTAGVDSTAQSRGGWKALGSLSRGRRQLLTHVCVCRWQQWAQDQMHRDQCKRTPDTGSPRKAKLHTAEGRQPPRPPPAVTVSTHKAKLHTAEGRRPPRPPPAVTVSTHKAKLHTAEGRRPPRPPPAVTVSTHKAKLHTAEGRRPPRPPPAVTVSTHKAKLHTAEGRGPPRPPPAVTVSTHKAKLHTAEGRRPPHPRPAVTVSTHKAELHTAEGRRPPHPSPTVTVSTAAGTRCTPGCSPLSNSTPPAQARGRPEPQGTWRQHPRRSRSTSGENPGHDSQAPTEPSLCMPKGCARKAGAQLPSSSIQ | **none** |
| **ORF40** [607833 - 609077] AFLRGRRLKLAGTRWTPTTLPALARGSRGAGRGAPGGVCAQARECALSRTGGRGRPVRRRRRKRARTSLPARAAPAAIGPEAASWRPAQPIGGAGAGRRAPGARGAAGGARGAGGAPGGAGRSGRSSSPPAGLSDAGARPVPSAARQPCDRAAALRAPGPPAARPRPGRSPRPPRERRGRRHGRARPPRPPRRARRAQHARAHEPVRHLGGGRLQPQLRAQVRAARRALFPPGTCWGCPGRGLCAPHPRPGSPSGAQVAPRAPARRFDACSRRPPAAPASAAPAPQVPSTGGRAQLPRTRCCSDPAGPWGSRAALTWLAATAPAHLPQCGAGRGDEGASSLGYHTLPFAFGDTVRPRPGSDPRRTGRAGLGCILRPARGGDALSCGAGSSSRVPGWRCLSFRGVAKPVLLFAPPS | **none** |
| **ORF41** [608005 - 609117] GAAGGSARAPHFRRALRRRRLDPRRRAGAPPSQSAAPARVGGRRARAGRPGARGARAGRRAGRGGAAAARRRPRACPTPGPGPSPPPPGSHVTAPPPSARPARPPRVRGPAAAPGRRGSGGAGAMAERGRLGLPGAPGALNTPVPMNLFATWEVDGSSPSCVPRYAPPAALCSRRAPAGGVLAAASARPIPGPGPPAEPRSPPAPPPAGSTRAAAAPPQLRQARPQPPRSRAPGAALSFRGPGAAQTRRDLGAPGRRSPGSPQPHLHTCLNAVPAGVTRVLPLSGTTRFLSRSATLSDLGLGATPGARGAQDSVVSSVQRAVEMPCPAGRGRPRGYLVGAVSHLEASPSQYCYLRLPVDLAIAEDWLFCTW | **none** |
| **ORF42** [752030 - 753880] KGGPSGGQACVPAHVSLMGLSQDGPEVLSHSWATGSKWAGPGHGAEQLQPGQTPGAGRRPRPRRGPCTFPASSLGGGENPKRSPKAAANLSGCPQLDAHGFWGGCRALLRSLGPRTRGVLATCGLLPAMLMQSVPGPWRLGSCVPGGSPRCPWPLVPGPWGAVCLGGVRAAHGPWSLAPGELCAWGESALPVAPGPWSLGSCVPGGSPCCPWPLVPGAWGAVCLGAVRAAHGPWSLVPGELCAWGESVLPVAPGPWSLGSCVPGGSPCCPWPLVPGAWGAVCLGGVRAAHGPWSLVPGELCAWGESVLPVAPGPWRLGSCVPGGSPCCPWPLVPGTWGAVCLGGVRAARGPWSLVPGELCAWGESVLPMAPGPWRLGSCVPGGSPCCPWPLVPGPWGAVCLGGVRAARGPWSLVPGELCAWGESVLPMAPGPWRLGSCVPGGSPCCPWPLVPGAWGAVCLGEVRAAHGPWSLAPGELCAWGKSVLPMAPGPWHLGSGVRGGSPCCPWPLVPGTWGAVCLGEVRAAHGPWSLAPGEWCAWGKSVLPMAPGPWHLGSGVPWGSPCCPWPLVPGAWGAVCLWGVPAWPTLWSAHLLSPALWAPSHGPWTAAAPSDSPACT | **none** |
| **ORF43** [752628 - 754127] GAVCLGGVRAAHGPWSLAPGELCAWGQSVLPMAPGPWSLGSCVPGGSPCCPWPLVPGPWGAVCLGGVRAAHGPWSLAPGELCAWGESVLPMAPGPWSLGSCVPGGSPCCPWPLVPGAWGAVCLGAVRAAHGPWSLAPGELCAWGESALPVAPGPWSLGSCVPGGSPCCPWPLVPGAWGAVCLGAVRAAHGPWSLVPGELCAWGESVLPVAPGPWSLGSCVPGGSPCCPWPLVPGAWGAVCLGGVRAARGPWSLAPGELCAWGKSVLPMAPGPWHLGSCVPGGSPCCPWPLVPGTWGAVCVGEVRAAHGPWSLAPGELCAWGKSVLPMAPGPWHLGSGVPGGSPCCPWPLVPGTWGVVCLGGVRAARGRWSLAPGERCACGESLLGRHCGQRISFPQLCGHLRTGPGLQQLRPTAQPAHERRSCLPRHHPGKWAQGGTGETWGGPWPRVAAPFFLPGPSTCPRYSGPLQRCCPTWTGLSYPPQTRPDCAEGAGRVVLLGLQ | **none** |
| **ORF44** [752026 - 754146] IEGRPFWGAGLCACPCVPNGAFPGWARGAFPLLGHWEQVGWAWSWSRAAAARADPWGRAKAEAQERTMHLPCQQPGRWRKPQKKPQGGCQSIGMPSVRRTRVLGWVPGPTPQPGAQDTRSPRHLRAAPCDAHAVCPWSLAPGELCAWGESALPVAPGPWSLGSCVPGGSPCCPWPLVPGTWGAVCLGGVRAARGPWSLVPRELCAWGESVLPMAPGPWRLGSCVPGGSPCCPWPLVPGPWGAVCLGGVRAARGPWSLVPGELCAWGESVLPMAPGPWRLGSCVPGGSPCCPWPLVPGPWGAVCLGGVRAARGPWSLAPGELCAWGQSVLPMAPGPWHLGSCVPGGSPRCPWPLVPGPWGAVCLGGVRAAHGPWSLAPGELCAWGQSVLPMAPGPWSLGSCVPGGSPCCPWPLVPGPWGAVCLGGVRAAHGPWSLAPGELCAWGESVLPVAPGPWRLGSCVPGGSPCCPWPLVPGTWGAVCLGEVRAAHGPWSLAPGERCAWGKSVLPMAPGPWHLGSCVPGGSPCCPWPLVPGTWGVVCLGEVRAAHGPWSLAPGEWCALGESVLPVAAGPWRLGSGVPVGSPCLADTVVSASPFPSSVGTFARALDCSSSVRQPSLHMSAAAASRDITLVSGPRAGQVRPGVAHGPEWLRPSSYQGPAPAPGIQDPCRGAVPPGLAFPIHPKLGQTVLRVQGGLCCWACSRGSLRY | **none** |
| **ORF45** [767497 - 768852] ARIPRRWCPPPLPCLGGRRRPPHTPYQQLLELFLEVAAPSFLSPALHPRSLQHHRQCWGGARAGAERDMETRTGLGAVPAGNLTPLPRRGCCYGNRATKGSGTGAARGGVGVGFSRSLRPLPAAERSLGAQGRECWTPRPKSLGLPESRGPGSPHPARRPGDGPARRPRRRHGNSRGAGGAAVARAGPCRRHLRREGPGGARPGPAAPHPPPPAGAPRPRGAPRRPPARGTGYRGPAPAAEPMGARTAGQAGLGAGGGGPEENGRRARADRAHRPWPPNAPSATRPCTSVSACPLPARSVHPAALRGQSRAAEPRRNSGCARPRPRTEDARPGGWLAALPKRLKPKGGAARKRGRSRPNPEAACGGGAGTTRAGLVVPRDEASVWGSARGSADASRFLGRRRAGRGRAWLYAPGASRALPWAWALSPGPPTPASPGNKWPGTVTPSSHSGLR | **none** |
| **ORF46** [774270 - 775268] GDGQAFVDRPALGVAQGQNPCPSVLVKVLVMTDSPLASSWSRLCPFSQGRWRLQGALPSPAPRLCCAPLLGCPLEGALSHSGSTEQKRGDLPQWLSPVLAPSGLPTLVYAPLLLAPHRSCLGPSFSEGHSDLATSRPLSPVLQGGRGFTPLPCSQPLAQLPAWPPSSPWAQTELSLDAEPIVPRAAIHVLAGPGLAPGSSRTTAALQPGTQCGLLPPGIQPKPPGPDVAEGTASPQPQAEGRIRQVPDPCSQERLGWFPFSSPPARPRLPCVCWGVVSGELRISGVPVSALAAGSQGCMGNSCFQLGGAGGGGGTGGLSWTPLPWEPPVETMS | **gag protein, partial [HIV1]**  **Sequence ID: AAR86605.1**  **E value: 7.1** |
| **ORF47** [819463 - 820668] CHLSDKMKPKSPNPSLPPGRAGEEGGGRSSFLSSALRRNLGRLGFSPKRTEGGAEGPGGQASPAPTSLSAPGPVGACPAEWGRSRGRQRSFRARGALGGTLGFWAVGEEGGDGTPRLGVREGAPLPRATSPARRRAVLSPWQPALRRGRGTGTPALPGPQVRRGSRAEGRGAGPLGRGRCRAGGGGARREEPARPRQWRQRLGGRARERRSICGRRGRRGRGRDWLGARLRPRRGRRAWATLHLWAVGTVGWGGGRCGRGEAWGRRGDGDPALRGEASGGQAETQPAGSGPSSAPARGGSTAAPSRPSSRGLRGVVMGRGSLRGPGSEGPGGPRVCARSEPRGVGGGGASAGAQETQDRWAWGQVPTERWPGRALACRGRGSARGWSPSLRQRRAPRPASAK | **envelope glycoprotein [HIV1]**  **Sequence ID: ALA27495.1**  **E value: 5.6** |
| **ORF48** [819572 - 820672] EETWEGWVSPQRGQRVARRAPAARPAPRLHPCQLLGRWAPVRLSGVALGGGSGVSGRGGHWAGPWDSGRWVRRVETGLRAWGCARAPRSPEPPVLRAAARCCRRGNRRCGGGGAPAPPRSQVPRSGGARGPRGAGRGLWVGGGAARAGEGRGGRSRRGPGSGGRGWAGGPGSGAASAGGGVGAGAGGTGWVRGCVRGGGGGPGRPCTSGPWGLWGGAGADAAGVRPGAGGVTGTRPCVGKPRGDKQRRSLLGAAQALHRPAAGAQQPPPAPPPGGSEGWLWGEGPCGARAVKGLEAPESVPGLSRGAWGEEGPRPGPRRPRTGGPGARSLRRGGRAGLWLAGVGAPLGAGVRPFASAEPLGPHLPSD | **none** |
| **ORF49** [820956 - 822152] GPSRHPPRDLSELRGVGVSCVWLWGSWAAGAGWGQGWECRVQRFPGSVGPAVSPGQSERPTVTGTLNFMPLSRHGSKRSPRPGAVEGVRGWLGVSLSVTLGWVRVCGPPLSPRCAQAQVRGLRQHGHEDQCQVCDRGAGACGSPTGLGGRASRDSRAATGIALAQRSKAGWLCRGRGWGTTPRQGNAEPWGAGQAVATRPGWWTGGETEPARAGRRGVGAAGQGRGGSERLVGRMHRDLGGSRSGIRKPGRAEASLGTPRAEGQARLPAQCGGGERVFPELRGNGLRPAARAWGPGRRSQEGGRRGPSSDKPPRAPGTSSAGRKRVRSVSFLAAGTRPGCSGEAAAPSGGLAVVRGRRFAHGRRRARCLGAPARRPRLVGSTRGAGGGGCWGRPLQPAP | **none** |
| **ORF50** [821815 - 823233] GQQRGRGVRAAGARREGGAGPAVTNPPGLRERARRAENASVPFPSWRRERGPGALARQQRPLEASLWFAAAGSRMGAGVRDASGPRRGDRAWWEARGVRGAGGVGGGRSSPHPDLVSSGLPRFRARRAVSRHARWLSAAGPRPARRGGRARPGRGPAADHGAAAPGPAPRVPDHAGDHGQHGRHGRVPGGAEPGPAQDRRVHHRAGGRRVLPAGAALRGRVGGRRGAHGQARLRHDPVVPLHLRAGDQALLHLPELQGGAARRGGPRGAQGADAAAVCVCARPVPAARGAGPHGVRAHLPQARGPARPPVLGGAGPAGPAGHAGQPVGAAALRAAAVGRGPHLLLLLHAAAGAAVRGAQRGQHAGRAHSAAEDDAVPGAQPRHRQCGGRAGARRQHGAVPGQPCLGHLRRQKRGGARHQGLHLPGVPPPGARLPAACAITGAAAATPAAQLGAAAAAAAARPAWAPPHVLAHA | **none** |
| **ORF51** [822224 - 823249] APQGLVPPGVGAARAQAGARRPTMVLPPPDRRHVCLTTLVIMGSMAVMDAYLVEQNQGPRKIGVCIIVLVGDVCFLLVLRYVAVWVGAEVRTAKRGYAMILWFLYIFVLEIKLYFIFQNYKAARRGAADPVARKALTLLLSVCVPGLFLLLVALDRMEYVRTFRKREDLRGRLFWVALDLLDLLDMQASLWEPPRSGLPLWAEGLTFFYCYMLLLVLPCVALSEVSMQGEHIAPQKMMLYPVLSLATVNVVAVLARAANMALFRDSRVSAIFVGKNVVALATKACTFLEYRRQVRDFPPPALSLELQPPPPQRNSVPPPPPPLHGPPGRPHMSSPTRDPLDT | **none** |
| **ORF52** [852460 - 853509] ADCEHTEAFLLSGALEPLGACRAWAWAWLLSSVLRRLLPPTHFRAQPPAPHPAAGRASPGEDPLLRSRVPCPPGTVAGGWCLKLVRLMQHPVGPFATIVKTRRPQARAGSRAGRVLGLLRGHWATPGGRVLTRVREHRMLPAEVGGAGPHRGAQASFPICPRCSPARPPGHSVPRSQRPQVTASPHPGNAGLWQRLGSQTTEPHKLTPVRGCHSHLRGRRWGGEGITVLDTDRAPALSPRQSRDPAPRAPLPLPLPLPLPLPVPLPVPLPLPRAREAPRGRRRSMLLSATRGQQSLSGCRSGSSFRPPGLGWRCLGVLPRGPRVKRPSALGAGHGDGCLGHGPGGPHTAG | **none** |
| **ORF53** [852471 - 853979] AHGGLPPLWGSGAPGGLQGLGLGLAAVLCAQEASPTHPLPGTAPSPTPSRREGIPRGGPSAPEPRPLSSRDSGRRLVPQARAPHAASRGSIRDHSEDPQAPGPSRLPGRPRSGASAGTLGHARRKGPDQGQGASDAAGRSGGRGAPPRSPGQLPHLPQVQPCKAPRPQRPQVTASPGHSVPTPRECRTLAEAGIPDHRAPQADACSRMPQPSPRQALGRGGDHSAGHGPGPSPEPKAEPGSGPESAPAPAPAPAPAPARAPARAPAPAPSPGSAAWSAAFDAAVGHTGAAEPQRLPQRVQFPAAGVGLAVPGGPPQRPACETSECAGRWTRRWVLGPRAWGTPHGRLGRPRQPRVGVPWGQEGRSLGCGAAGTSGEGPGGQRGGSRDREARGGAGFVAAPEPPLKLPSAASCGAGSASLAGHPHPPPRGVRQPPFQPTQMVSSPSRHSEPRCRCETPPCIPGRPSAGPSPPHRQSHPSRPCPIRSPWPHHRIAGRRAYATSMK | **none** |
| **ORF54** [862083 - 863084] GGGSGDSASSYVCTAVCPPCIPWWTLVGLPGRRPQTWADSRTQRDGAEAGMTETDTKQRDGGRGRHAESRRHPGSGPDSQGPPPGRTRSAPPHPHLTHTLTPTYPHPHTPRDTQTPTATHSHSRTYPVTSHTLPPNPYTTHLHNPYTTHTHKLYTTHTPTIHLYTLTHPYTTHSHTTHTPHAHNPYTYTHSHLQTIYYTLTHNPYTYTHTPIYHTQPYTTHTNHIPHTHTTHTPIHTHTHIPHTTNIPHTHNPYTYTHTPIHHTQPIHYTITPQTVYHTLTHNPYTYTHTPIYHTHNLCTYTHTYTTHTQLLHLHTPTTHTHTAYTPTHTHTLI | **none** |
| **ORF55** [862237 - 863775] QRQTQSRETEAEGDMQSRGDIRAQAPTLRGHPQGGHAVHPHTHTSHTHSHPHTPIHTHPETHRHPQPHTHTHELIQSRPTHSHPTHTPHTYTTHTPHTLTNYIPHTHQPYTYIHSHTHIPHTHTQPIHHTHTTHTPIHTHTYKLYTTHSHTTHTPIHTHPYTTHNHTPHTQTIYHTLTQPIHLYTLIPIYHIQPIYHTHTTHTPIHTHLYTTHSPYTTQSHHKLYTTHSHITHTPIHTHPYTTHTTCAPTHTHTPHTHNSYTYTHPPPIHTQPIHLHTLIHSYSTHTTHIPHTQPTYLYTHAHTPHTHTTHAPHTPTNYMLHTHTQPIHLYTIPQPIHTYTTYTPHTLTICIPHTQPIQLYTHTHIPHTHTQPIYHTLTHKPYIYTHTHMPHIQPIHHTLTHNTYTTHTTHRTQTQVITTHTAHIPIHIHTHNPYTTHTTHTPIHTLNSYTTHTQPIHLYTNSHTIHIPQTHTQRIHLYTHMQLIQHTLTQPTDHKHTQAITTHKPIHLYTHS | **none** |
| **ORF56** [869104 - 870216] TCCHLWDQDPGAAPRLAGRAGEPTLAGPPANPHPRSPKEAERRWPWWPGHRQPDWYKACLVQARTSPTLMAWWSPLYRENTEAQQGQGHGLRLHGDGGWVSGPGGRGGCSTRTAWAVASLLPALSHPSPSEPPAPSFPLSLSLATLLSPCLSASLKVSPCPNLCLCPIVGVSDSSLSSPSAGLCPAILPVLASLSFCVSPSVPPFSIHLSPVSVSIPPSLFPSIPPSVPVPLLCPFLCLCLSVCLFVSLCVSLSVSLLLFLYLCLYLSLFVSLCVCLSLLCLSMSLSISLSLCLCISVCLFVSLCMSLSVSFCISVYICLSHLSLPVCVSLSLCLCLCLSVSSSLSVSACLCHLVSLFLYVSLFVCVSSSL | **none** |
| **ORF57** [869570 - 871183] RYLPARISVSVPLSVSLTHLCQARLLGFVPPSCLSWPLSLSVCLRLCLLSPSISPPCLSLSLRLSSRLSLPLCLSLCCVPFFASVSLSVSSCPCVCLSLSLCFSFCISVYISASLCPCVCVSLFSVFPCLCLYLCLSASVSLSVCSCPCVCLSLSLSVSLSISVSLICLFLCVSLCLSASVSVSLSLHLCLSLPVCVIWCLCFCMSLCLSVSLPLCSSASLYLSLYLSFSLPVSFSVSFCLSESVSVSVSSSLYIPLCLSVAVCLCFSSPLSISLCLSFSAFLTLSFSVSLSLSQSLFLSLSLSLSLSVSLCLCLSLISIALSLSLFLSLCISISVSLMSISLSFSVSLYLCLSSLSLSLSFCLYLCLSISFSVSLYLSVSISLCLSLCLCISVSISVSLSLSVSPCLSLSLSLHPMWVCGFPRGSEPCPGQHRQGTGRGLGGGPVTLGARPGGCRWARAAVPPTHRQAPALAQHPLRRSQLQAELCPFAAGPCVFGAFPEPGLGWALFPQTTHEQSVGHVPSSQRGPLRRGVVGTQQ | **none** |
| **ORF58** [874032 - 875129] LCERGLHLSSWAHLAASFSPCVFSASVVFSLHVCLHVSFSLFPSPPFFSHLCLCFLSISSHLSLSVSCVSVPPRPCLLSLSIPISLSVSVFVPVSMSLVSPHPCLSLCLCVSYLSPSLSLYVSVSLISPCPCLSLSTSLCLSPSLSLSMSLCLLSLPIPVSLCLCVSYLSLSLSFSVYVSVSLPIPVSLSLCLLSLPIPVSLYVSSSLISLPIPVSLCLYVSYLSLSLSFSVYVSCLSPSLSLSLYVSVSLISPHPCLSLCLFISYLSPHPCLYVSVSLISPHPCLCLCLCVSCLSLSLSLSMSLCLSGPLVQCPLPRTPVSLVWGAGPSVVLWAVLVLGTACQGSRSSPRAGHCVLALAKGPGES | **none** |
| **ORF59** [874057 - 875151] VPGPILLPLSLPVCSLPLLCSLCMSVSMSLFPCSHLLPFSPISVSVSYLSHLISLFLSLVSLSLPVPVSYLSPSPSLSLCLSLSLSLCLLSLPIPVSLYVSVSLISPHPCLSMSLCLLSLLVLVFLCLRLCVSLHPCLSLCLCVSYLSPSLSLYVSVSLISPCPCLSLSMSLCLSPSLSLSLCVSYLSPSLSLSMSLHLLSLSPSLSLYVSMSLISPCPCLSLSMSLVSPHPCLSLSMSLCLLSLPIPVSLYVSSSLISLPIPVSMSLCLLSLPILVSVYVSVSLVSPCPCLSLCLCVSLGPWSNVPFPEPLFPWCGVQVLLWSCGPCLCWAQPARAAAQAPGLGTASWLWPRALGRAEGGACRV | **none** |
| **ORF60** [874040 - 875260] EGLAPEFLGPSCCLFLSLCVLCLCCVLSACLSPCLFFPVPISSLFLPSLSLFLIYLISSLSFCLLCLCPSPSLSLISLHPHLSLCVCLCPCLYVSCLSPSLSLSMSLCLLSLPIPVSLCLCVSYLSLSLSFSVYVSVSLSIPVSLYVSVSLISPHPCLSMSLCLLSLLVLVFLCLCLCVSPHPCLSLSVSLISPHPCLSLCLFISYLSPHPCLSMSLCLLSLLVLVFLCLCLLSLPIPVSLSLCLCVSYLSPSLSLSMSLHLLSLSPSLSLCLCVSYLSPSLSLSMSLCLLSLPVPVSLYVSVSLWAPGPMSPSQNPCFPGVGCRSFCGPVGRACAGHSLPGQPLKPQGWALRPGSGQGPWGELREVRAGCEAHHSHCQMLSAGASLGLLCGGHSPREGCPWRRPAA | **none** |
| **ORF61** [879142 - 880398] KPLPRPGPGSHGLEGFSVAGLPQVTCCSQLPSGDWTLRGDSGLHLTLALRAEGGSQKAGAPGEARKMSKARAGASLPPCGSLGARCPHSGCGTVLSLLGAPGRSQRHAEGPGQALSHWPASDATPRGPSLSMLWVLVGLRKGWETWPGREQGLPGRGLWSWSLSCAQWKHREARGAWGSRLSLGAWEGTPLAFLELGLGCERSTPAGLRRICECECACVPTCPVQSSVVKGRCPFLHASVGRPEAHPLGWWGPPGACPPSGALESSPSLPRHSCHYLRLSWLACSFYPPYSLPATARGGSLRDKRPCHCSLRTVRGMCVSQNPSWAVPALADLRSHTSEASCTTHSHTHNSHSHQGGKSHGGARGPPRLRAGGILPCPCPGPFPSAETLRMDRQAPDGSVGLLGGAWSLFRGGDRRAAQ | **none** |
| **ORF62** [886819 - 887826] SRVSFTSKSLTIQGALTSKSLTIQGALTSKSLTIQGALTSKSLTIQGALTSKSLTIQGALTSKSLTIQGSLTSKSLTIQGALTSKSLTIQGALTSKSLTIQGSFTSKSLTIQGSFTSKSLTIQGSFTSKSLTIQGALTSKSLTIQGALTSKSLTIQGALTSKSLTIQGSLTSKSLTIQGALTSKSLTIQDSFTSKSLTIQGALTSKSLTIQGSFTSKSLTIQGALTSKSLTIQGALTSKSLTIQGSLTSKSLTIQGALTSKSLTIQGSFTSKSLTIQGSLTSKSLTIQGSLTSKSLTIQGALTSKSLTIQGALTSKSLTIQGSFTSKSLTIQGALT | **none** |
| **ORF63** [886392 - 888296] TGGFSSNIHFSGKLEVQGQDGSRVKFQEGPIPGSQTPIFSLRPHMVERGQGCSLGSLFFWVSSEIRDRVTFLSVPPQALTTSLRPQLCMLSLWDDGCHTGVSGRHRPSDHSTTPVCLCLSPSLCLLVCLQLGHLSSDLAPSIIQGVLHLEILDNPGRPHLEILDNPGRPHLEILDNPGRPHLEILDNPGCPHLEILDNPGCPHLEILDNPGFPHLEILDNPGRPHLEILDNPGCPHLEILDNPGFLHLEILDNPGFLHLEILDNPGFLHLEILDNPGRPHLEILDNPGRPHLEILDNPGRPHLEILDNPGFPHLEILDNPGRPHLEILDNPGFLHLEILDNPGCPDLEILDNPGFLHLEILDNPGRPHLEILDNPGRPHLEILDNPGFPHLEILDNPGRPHLEILDNPGFLHLKILDNPGLPHLEILDNPGLPHLEILDNPGRPHLEILDNPGCPHLEILDNPGFLHLEILDNPGCPHLEILDNPGFLHLEILDNPGRPHLEILDNPGFLHLEILDNPGCPHLEILDNPGFLHLEILDNPGCPHLEILDNPGFLHLEILDNPGLPHLEILDNPGRPRLEILDNPGRPHLEILDNPGCPHLEILDNPGCPHLEILILMGSAKALLTNNAALNGPRG | **none** |
| **ORF64** [910485 - 911669] PESCLLLCNPLGSRGAGAPSRVGFVDSRATSSSRSTCSDRSILPLPLATETSVCWGDHKCHNSRVTPGMSHHVTPHHTMSCQGCHATSHHETHHHTRDVVPCHTPSCHITPCHTASHHTKDVAPCHSTSHVTSCHTTPHHTTDVIPHHTTSHYITPRQTTSHPITPGMSHPITPHHMSHHVTCHTISQYVTACYTSSHHITPGMSHLVPSHHIMSHHSTSFHIIAHHTRDVIPITLCHSTSHQGCHTVSHHIPPCATPPHTRDITPCHTTSHHVQHHLTPGTLQHVTPHHITPRISHCHTTSYNITPCPVTSCHMTSTSHQRHHTTSHHVMLHHSTDCWGMCRGRPQCSLAGELREGPGAGHGVPSGGLTLWRMLGPRWKGGGGPRVAQGGAQFP | **none** |
| **ORF65** [910775 - 912178] GCRTLPHPILPHHTMSHRITSHQGCGTLSQHVTRHIMSHHTSSHHRCHTPSHHITLHHTTSNYLTPHHTRDVTPHHTTSHVTPRHMPHHITVCHSLLHFITPHHTRDVTPCPITSHHVTPFHIISYYSSSYQGCHTHHTMSQHITPGMSHCVTPHPTMCNTTSHQGHYTMSHHIPPCATPPHTRDITTCHTTSHHPSDITLSYHIIQHHTMSCHIMPHDIHVTPETSHHVTPCHVTSQHGLLGYVQGPPTVQPCWRVEGGSWGWAWCSLRRADPLEDARSQVERGRWAPGGSGRGPISLGEPGPGARPCRGRSCRKHLLLPNSACSVHGMTRSPAPSWVSFPYPGQAVHPQTVDWSPPHQGTRRPVGPLEGQRVEISPAGSLRAGTFSGQSSPASLGRHPWAWDRAGDAAAARPAPQVLSPSPPAGLQQRVLRVKGLGFSTQFRPGPLEPTLQWRAFPRHGRAWSLGI | **none** |
| **ORF66** [943035 - 944489] GQRDWADQKPEEVSHRKCPEWNRKCPEWKRKHPAWNRKHPEWNKKGPAWKRKHPEWNGKRPEWNRKHPECNRKHPEWNRKCPAWNRKHQECNRKHPEWNRKCPAWNRKHPECNRKHPEWNRKCPAWNRKHPECNRKHPEWNRKCPAWNRKHQECNRKHPEWNRKCPAWNRKHPECNRKHPAWNRKHPEWNKKRPAWNRKHPACNRKHPEWNKKRPAWNRKHPAWNRKHPAWNRKHPEWNKKCPAWNRKHPEWNGKRPAWNRKHPEWNKKRPAWNRKRPAWNGKHPECNRKHPAWNRKRPAWSRKRPVWSRKCPARNRKCPAWKRKHPAWNRKHPEWNKKRPAWNRKRPAWNGKHPECNGKHPAWNRKRPAWSRKRPVWNRKHPEWNRKHAEWNRKCPAWNRKHPAWNRKHPAWNREHPEWNRKCPAWNRKHPGWNRKHPVWNRKHPECNRKHPAWNRKHPAWNRKHPAWKRKRPAWNGKRPEWAL | **none** |
| **ORF67** [942991 - 944514] GRTNVEVGGKGGWYEARGTGQTKSPRRCRTGSVQNGIGSVRNGKGSIQHGTGSIQSGTRKVQHGKGSIQSGMGSVQSGTGSIQNVTGSIQSGTGNVQHGTGSIKNVTGSIQSGTGNVQHGTGSIQNVTGSIQSGTGNVQHGTGSIQNVTGSIQSGTGNVQHGTGSIKNVTGSIQSGTGNVQHGTGSIQNVTGSIQHGTGSIQSGTRNVQHGTGSIQHVTGSIQSGTRNVQHGTGSIQHGTGSIQHGTGSIQSGTRNVQHGTGSIQSGMGSVQRGTGSIQSGTRNVQRGTGSVQHGTGSIQNVTGSIQHGTGSVQPGAGSVQCGAGSVQRGTGSVQRGKGSIQRGTGSIQSGTRNVQRGTGSVQHGTGSIQNVTGSIQHGTGSVQRGAGSVQCGTGSIQSGTGSMQSGTGNVQHGTGSIQHGTGSIQHGTGSIQSGTGNVQHGTGSIQDGTGSIQCGTGSIQSVTGNIQRGTGSIQRGTGSIQRGRGSVQHGTGSVQNGHFEGKSCPSH | **none** |
| **ORF68** [953959 - 955257] GCHTLSHYITSCHTIPHQTILCPIIPGMSYHIILCHTASHQGCNTTSHHVQPCSPTPHHRRDITPCHTTSHHATPHHMREVEPCHTPSCHITPCHTALHHTNDVTLCHTTSHMSHQRCHTPSHHITSHPLNDIIPHHTRDVTPHHIRSYHITLRHTTSHQGCHTLSQDVPPCHTIPHHTISYPIILGMSCPIPLCHTTSHHVQPCHITSHHITPCHTRDVTLCHTTSHHVLSHHTLHPGTPWISHHITPCHVTSQHGLLGGAWRQPCWRVEGGSWGWAWCSCRRTGPLEDARFQVERGRWGLGASGRGHPWAWDRAGDAAAARPAPQVLSPSPPAGLQQRVLRVKGLGFSTQFRPGTLKPTLQRRALPRHSRVRALGISPPTLVQLQLLDATQTNPTTPPSWVRWSLSCHRQLRRHFPATAATAATAVTRPCP | **envelope glycoprotein [HIV1]**  **Sequence ID: AEQ75947.1**  **E value: 9.2** |
| **ORF69** [972874 - 973926] ADSEHTEAFLLSGALEPLGACRAWAWAWLLSSVLRRASPTHPLPGTAPSPTPSRREGIPRGGPSAPEPRPLSSRDSGRRLVPQARAPHAASRGSIRDHSEDPQAPGPSRLPGRPRSGASAGTLGHARRKGPDQGQGASDAAGRSGGRGAPPRSPGQLPHLPQVQPCKAPRPQRPQVTASPHPGNAGLWQRLGSQTTEPHKLMPVRGCHSHLRGRRWGGEGITVLDTDRAPALSPRQSRDPAPRARLALPLALALPLPLPLPLPLPVPLPLPLPRAREAPRGRRRSMLLSATRGQQSLSGCRSGSSFRPPGLGWRCLGVLPRGPRVKRPSALGAGHGDGCLGHGPGGPHTAG | **none** |
| **ORF70** [973269 - 974396] PGSGSIGCCRQKWGARGPTEEPRPASPSAPGAALQGPQATASPGHSVPTPRECRTLAEAGIPDHRAPQADACSRMPQPPPRQALGRGGDHSAGHGPGPSPEPKAEPGSGPESTPGPAPGPGPAPAPAPAPAPARAPAPAPAPSPGSAAWSAAFDAAVGHTGAAEPQRLPQRVQFPAAGVGLAVPGGPPQRPACETSECAGRWTRRWVLGPRAWGTPHGRLGRPRQPRVGVPWGQEGRSLGCGAAGTSGEGPGGQRGGSRDREARGGAGFVAAPEPPLKLPSAASCGAGSASLAGHPHPPPRGVRQPPFQPTQMVSSPSRHSEPRCRCETPPCIPGRPSAGPSPPHRQSHPSRPCPIPSPWPHHRIAGRRAYATSMK | **none** |
| **ORF71** [981724 - 982743] DPEGWGRGRDDRDRHKAERRRQRETCRVTETSGLRPRLSGATPREDTQCTPTPTPHTHTHTHIPPSTHTQRHTDTHSHTLTLTNLSSHLSSEAGMTETDTKQRDGGRGRHAESRRHPGSGPDSQGPPPGRTRSAPPHPHLTHTLTPTYPHPHTPRDTQTPTATHSHSRTYPVTSHTLPPNPYTTHLHNSYTTHSHSQTIYHTLTHPYTKHAQPIHHTLTLTNYVPHSHTPTIHLYTLTHPYTTHTQLIHHTLTLTNYIPHTHIPIYHIHTTHTSDTHTQPIHLYTLTQPIHLYTLTPIYHIQPIHHTHTTHTPIRTHPHTTHTAHTVHTHTTNYVAHTHT | **none** |
| **ORF72** [981756 - 982772] QRQTQSRETEAEGDMQSHGDIRAQAPTLRGHPQGGHAVHPHTHTSHTHSHPHTPIHTHPETHRHPQPHTHTHELIQSPFQRGRDDRDRHKAERRRQRETCRVTETSGLRPRLSGATPREDTQCTPTPTPHTHTHTHIPPSTHTQRHTDTHSHTLTLTNLSSHVPHTPTQPIHHTPTQLIHHTLTLTNYIPHTHTPIYQTRTTHTPHTHTHKLCTTQSHTNHTPIYTHTPIYHTHTAHTPHTHTHKLYTTHSHTHIPHTHNPYIRHSHTTHTPIHTHTTHTPIHTHTHIPHTTHTPHTHNPYTYTHTPTHHTHSPYSTHSHHKLCSTHSHITHTHIHTHI | **none** |
| **ORF73** [982730 - 983764] HTLTHNPYTYTHTYITHTHTARAHIHSHTYFPHTTHTTHTTHTYIHTHTYTTHSAYTIYTPHYIYHTHIYNTYTYTHTPMSHTHTPCTYIHTHTQNTHNPYPYIYTHTPPIHTQSIHLHTLIEHTHTTHIPHSHTAHIPIHTHTPIHHTFTQLHHIHPQTICYTLTHRPYTYIHSHSHIPHTTHIPHSHKLYATHSHKLYTTLTHNPYTYTQSHNPYTTHNPYTTDTFTIYIPHKPIHLYTLTHPYTTHSHTAHIPHAHTQMIHLYTHPYATHTTHTPHTQTHTPHTQPIDHKHTQAITTHTHSPYAYTHSHTQPIHCTHNTYTYTHSHSQFIHHTLTHNPYIYT | **none** |
| **ORF74** [983625 - 984878] PHTHTAHMPIHIHTHNPYTAHTTHTPIHTHTHNSYTTHSHTTHTSIHKLTHNPYTTNTHNQTPIHTHTTHTAHTQPTDHKHTQAITTHTTHTPIHTLINKPYTPPHTTHTPHTHTQPIHLYTHTTHIPQTHTQPVHLYTHTTYTPHTHTQPTDHIHKPYPHTHNPYTCIHTHKQATHLHTLIHNPYTTHSHTTHTPVHTLTHITHIPQTHTQPIHLYTHTTHTPHTHTHIYHTTHTTHTRNTHTHTHTPPTHTTHTPHTQPIHLYTHTNSCTTKTHNTYTYTHIQPIHHTFIHNPYTTHTTHRPIHTQHMQLSHTHNTYTSYTHTQHAHFLYTQPTRLYTLTPSLTLTLTFIYSLTHFHTHTLTHTYTPTHTQTPTLSHCHICTHAHTLPPSHAHPPPAPGLTGVCPRNPASGVPSVS | **none** |
| **ORF75** [1004827 - 1006131] LTRAWSSSAQLSSAQLRPAQPGPARLGPAQSGPARPNPARPSSARPSPAQPSSAQPSLAQPNPAQPSPAQPSPAQLIPAQPSSAQLSPAQLSPAQPSPAQLIPAQSSSSQPSPAQLSSAQPSSAQPSPAQHSTAQSSLAQLSPAQLSPVQLSPAQPSPAQPSSAKSSSAQPSPAQPSPVQLSPAQLSPAQPSPAQPSPAQPSSAQLSSTQPSPARPSPAQPSSAQPSPAQPRLAQPSLAQPSSAQPSPAQPSPVQLSPAQSSSAQTSSAQPSSAQPSSAQLSPDQFSSAQFSLALLSPAQPRAAQGSPHGRVSAGSTSSSLPSGSAQPRPQLSHETCSPCIPGRAGSSLVRPGQPGSAPSAQILPAWLGSPALWFRSPCGDLFCPSLLSWCLFSTFCFQKGPGPAWLDSSMCRGGDGGLETSKTHLVRLLEGPQC | **none** |
| **ORF76** [1007796 - 1009148] SRVSFTLKSLTIQGALTLKSLTIQGALTSKSLTIQGSLTSKSLTIQGALTSKSLTIQGALTSKSLTIQGSLTSKSLTIQGSLTSKSLTIQGALTSKSLTIQGALTSKSLTIQGSLISKSLTIQVSLTSKSLTIQVSLTSKSLTIQGALTSKSLTIQGSFTSKSLTIQGSLTSKSLTIQGALTSKSLTIQGALTSKSLTIQGSLISKSLTIQVSLTSKSLTIQVSLTSKSLTIQGALTSKSLTIQGSFTSKSLTIQGSFTSKSLTIQGALTSKSLTIQGSFTSKSLTIQGALTSKSLTIQGSFTSKSLTIQGALTSKSLTIQGSLISKSLTIQGSLTSKSLTIQVSLTSKSLTIQGALTLKSLTIQGSFTSKSLTIQGSFTSKSLTIQGALTSKSLTIQGSFTSKSLTIQGALTSKSLTIQGSFTSKSLTIQGSFTSKSLTIQGALTSKSLS | **none** |
| **ORF77** [1007363 - 1009207] TGGFSSNIHFSRFSKLEVQGQDGSRVKFQEGSIPGSQTPIFSLRPHVVERGQGCSLGSLFFRVSSEIRDRITFLRVPPQALTTSLRPQLCMLSLWDDGCHTGVSGRHRPSDHSTTPVCLCLSPSLCLLVCLQLGHLSLDLAPSIIQGVLHLEILDNPGRPHLEILDNPGCPHLEILDNPGFPHLEILDNPGCPHLEILDNPGCPHLEILDNPGFPHLEILDNPGFPHLEILDNPGCPHLEILDNPGCPHLEILDNPGFPHLEILDNPGFPHLEILDNPGFPHLEILDNPGCPHLEILDNPGFLHLEILDNPGFPHLEILDNPGCPHLEILDNPGCPHLEILDNPGFPHLEILDNPGFPHLEILDNPGFPHLEILDNPGCPHLEILDNPGFLHLEILDNPGFLHLEILDNPGCPHLEILDNPGFLHLEILDNPGRPHLEILDNPGFLHLEILDNPGCPHLEILDNPGFPHLEILDNPGFPHLEILDNPGFPHLEILDNPGCPHLEILDNPGFLHLEILDNPGFLHLEILDNPGCPHLEILDNPGFLHLEILDNPGRPHLEILDNPGFLHLEILDNPGFLHLEILDNPGCPHLEILILMGSAKALLTNNAALNGPRG | **none** |
| **ORF78** [1024563 - 1025999] SKPQKNVTLPSCFFLFHLYPPPAFPPFNIEAGRSVTASEVRCGVVHVDSEEGGPCPWWSGFPGRWPDVGAEGKRPVQSLDGREHVHCAEWAGRDPQRRQCGSIASTGDGNVGEEPGTWSSSRCEVVTRPGSLCLSVLGGCWGCKHSCACEARVCMCVSRVPMHGRHRRVSPTRGRHCCVATMHGRHCRVSPVRGSIVAWPPFVAGMCSLSTMPDGVFCLLQARPCRVSRELPHGTDVLSPPGLALSCHVSSELGHGSGGHRRGCAGPHWLDSVGTPYPLLSVGSRGVEVPSGRWSGRGRVGSVWGDGCSARLLPCGGAQLKPGLSLPSPLPPQHPPSARLFSLPRTPRSQPRSVSSQGHVVAGPSSCLTRVLLAALQAHLGHVGQGHLEGWASWTQGPPIPPGQHAQMVPFSPFPWPQRPLYPGVTTNVTTQESHQERHTMSHWGKERSDCYCAYVEKEHIRNSILICTKKNCSALRRC | **none** |
| **ORF79** [1054879 - 1055952] EDRRSFSSTLCWVDAEVTALLCVSSQGHVVAGPSCSLTRVLLAALQAHLGHVGQGHLAGWASWTQGPPIPPGQHAQMVLFSPFPWLQGPLYPGVATNVRTQESHQECHTMSHHISLCHARDVTPHHTMKLIITLGMSYPATPHHTTSHHLTPGTLYHVTPHPTMSRHLTPHHIISHHVTLCHSLLRFITPHHTRDVTLCHTTSQHVQRHLTPGTLHHVTPHHITPRISHRHTTSYNITPCPVASCHMTSTSHQRHHTTSHHVMLHHSMDCWGMCRGRPQCSLAGELREGPGAGHGVPSGGLTLWRMLGPRWKGGGGPRVAQGGAQFPQGNLVQAPGPAGAGAAGSICFFPTQPAQCTE | **none** |
| **ORF80** [1054926 - 1056368] GHCLALCLLTRPRGGRSFLQSNQSPACCSASPLGSRGAGAPGRVGFVDSRATNSSRSTCSDGSILPLPLATGTSVSWGGHKCQNTGVTPGMPHHVTPHLIMSRQGCHATSYHETYHHTRDVVPCHTPSYHITPPHTRDIIPCHTTSHHVTPPHTTSHYITPRHTMSQPVTLHHTTSHQGCHTVSHHIPACPTPPHTRDITPCHTTSHHPSDITPSYHIIQHHTMSCRIMPHDIHVTPETSHHVTPCHVTSQHGLLGYVQGPPTVQPCWRVEGGSWGWAWCSLRRADPLEDARSQVERGRWAPGGSGRGPISPGEPGPGARPCRGRSCRKHLLLPNSACSVHGMTRSPAPSWVSFPYPGQAVHPQTVDWSPPHQGTRKPVGPLEGQRVEISPAGSLRAGTFSGQSSPASLGRHPWAWDRAGDAAAARPAPQVLSPSPPAGLQQRVLRVKGLGFSTQFRPGPLEPTLQWRAFPRHGRAWSLGI | **none** |
| **ORF81** [1132622 - 1133719] WESVGRTWAPAQMGPRRGGGSGQGVPVPGGQPWVGAAASGKTLWRGQGGWGTEKQLLLQGAVPAGSGWAAAPQVAHCCGAHLCEAGRVRHCPQRGFSVRPPHRVASGEGPCRVRAAGSPSGQKSLGQPSALPLVDLGVFLSLALQGGRGPGCLAPAYTRGPSMPPLAGQLGHRQQALSRADSLLLCVQPLVATTSHWGHARAVGALVCVPCLKGAEKGGPGRRLGAVTLGGRPLWACVGLTFRWALSPSLTSGGLWRPLWLPEVLTVVLDSQMSPERAAVQGDHIHGHTREGLCPAPSRGECLGRKPPLLRARRGVVSTSYTVPASLGQHVVWTARMDAGTSMAQEYWEVCAPAGHREGACVPRTA | **none** |
| **ORF82** [1720677 - 1721765] GSKSKHSKASRRQEITKIRAELKEIETQKTLQKISESRSWFFEKTNKIDRPLARLIKKKREKNQIDAIKNDKGGITTDPREIQTTIREYYKHLYANKLENLEDMDKFLDTYTLPRLNQEEAESLNRPITGSEIGAIINSLPNQKSPGPDGFTAEFYQRYKEELVPFLLKLFQSIEKEGILPNSFYEASIILIPKPGRDKTKKENFRPISLMNIDAKILNKILANRIQQHIKKLIHHDQVGFIPGMQGWFNIRKSINVIQHINRTKDKNHMIISIHAENAFDKIQQHFMLKTLNKLGIDGMYLNIIRAVYDKATANIILNGQKLEVFPLKTGTRQGCPLSPLLFNIVLEVLARAIRQEKEIKDI | **none** |
| **ORF83** [1721769 - 1723526] LGKEEVKLFLFGDDMIVYLENPIISAQNLLKLISNFSKVSGYKINMQKSQAFLYTNNRQTESQIMSELPFTIASKRIKYLGIQFTRDVKDLFKENYKRLLNEIKEDTNKWKNIACSWVGRINIVKMAILPKVIYRFNAIPIKLPMAFFTELEKTTLKFVWNQKRACIAKSILSQKNKAGGIMLPDFKLYYKATVTKTAWYWYQNRDIDQWNRTEPSEIMPHIYNYLIFDKPDKNKQWGKDSLFNKWCWENWLAICRKVKLDPFLTPYTKINSRWIKDLNVRPKTIKTLEENLGNTIQDIGMGKDFMSKTPKAMATKAKIDKWDLIKLKSFCRVKEITIRVNRQPTEWDKIFATYSSDKWLISRIYNELKQIYKKKTNNPIKKWAKDMNRHFSKEDIYAAKRRMKKCSSSLAIREMQIKTTMRYHLTPVRMAIIKKSGSNRCWRGCGEIGTLLHCWWDCKLVQPLWKGVWRFLRDLELEIPFDPAIPLLGIYPKDYKSCCYKDTCTRMFIVALFTIAKTWNQPKCQTMIDWIEKMWHIYTMEYYAAIKNDEFMSFLGTWMKLETIILSKLSQGQQTKHRVFSLIGGN | **none** |
| **ORF84** [1985911 - 1984622] (REVERSE SENSE) GALLRITRGLSEPPRVTQETRGRSGHQGELRTTRWYSGHQGALMTSRACLGLYEACRRSRPCSGQQGAHRTTWGCSEPLGVTQDSSRHSGHHGALRTCRGRSEHQGKVRKTWGHLEPPGGTQDSRGRSGPLGGAQDQQGVLRTPWGMQEQQAVLRVTGGSQNNLGVLRTTRGHPGHHAALRTCRGRSGHQGKLRMTWGHSEPPGGTQDSRGRSVPRGALRTAGGAQDTVGCSGPARGTQDHQGALGGAGGTQDTRRGSEPLGGTQNHQGALRTLGAFRTTSRFSGHPGTLRTAGALRTPGRVLRTPGGSQKHLGVTRTSRGMQDTREGAQDTRGCSGQQGAQNHEGAFQTTKKYSGPGVTETPKDTQDHQEALTTAQSAEDTRASGRTGGAQATMGGSGTTRVSLRQQGSLRRQLHIRCLGRVRFAFRPC | **none** |
| **ORF85** [1497684 - 1496575] (REVERSE SENSE) PPIAFRYDRMGGSSPSTPTERGTSPAADERSAPFLVYVPFSTSDLYNWKTHNPPFSEKPQALTSLMESVLRTHRPTWDDCQQLLLTLFTSEEREHIRREARKHFLASAGGPEEEARDLLEEEEARDLLEEEEARDLLEEEEVFPSTWPNWGPDSSGGRRALDDFHQYLLAGIKGAAWKPINLSETTEVVRGPDESPGVFLEPLQEAYRIHTPFDLATPENSRALNLAFVAQAAPDIRRKLQKLEGFTGMNISQLLEIAQKVFENREFEKQKQATQAAEKAADKAYKRQAKILVTAIQEGRKERPAFQKNGQGTSGSHQKSERGEQAPLGKHQCAYCKQTGHWKKECPLLPKEKSENKKVLTLAATEEPDD | **gag protein [Galidia ERV]**  **Sequence ID: AGV92852.1**  **E value: 2e-85** |
| **ORF86** [1496148 - 1494859] (REVERSE SENSE) SPEENTNTLLLDLQTLFPQVSAESNPPGLAKHHPPVVIELLATAIPVQAKQYPTSQQAREGINPHIQRLLQAGILTPCQSAWNTPFLPVQKPGTNDYWPVQDLRDVNKWTVAVHPTIPNPYTLLSLLPPEHTVYTVLDLKDAFFAIPLAPKIQPIFAFKWTDPRSGDTTQLTWTQLPQGFKNSPTLFGEALQQDLIPFRASHLNCTLLQYVDDILITTETMDGCLQHTRDLLYLLQELGYGVSAKKAQLCLPRVSYLGYKINKGKKALTSARKEAILRIPTPATKRQVHEFLGALGYCRLWISGFAEITKPLYTATKGNGPLIWTDTEEQAFQNLKKALTAAQALALPNISKPFHLFVHESQGVAKRVLTQTLGTWRGPVAYLSKKVDPVASGWPSCLPAIVATASLVQETDKLTLGQNLTLTVPHAVET | **polymerase, partial [Chick syncytial virus]**  **Sequence ID: ABC26828.1**  **E value: 1e-177** |
| **ORF87** [1492592 - 1491126] (REVERSE SENSE) DTYQFVRPDWACVTLATYSGGSTRSPTLSISRASRPKSCSKNNCNPLNIIVHEPNSAQWYYGMSWGLRLYVPGFDVGTMFTIQKKILVPWSPPKPIGPLTDLGDPMFQNHPDKVNLTVPPPFSVPKTQLQRHQLQPSLMSILGGVHHFLNLSQPTLAQDCWLCLKAKPPYYIGLGVEVALKGSPLSCHAQPHAFTLGDVSGSASCLISTGYDLSISPFQAICNQSLLTPMSISVSYQAPNNTWLACTSGLTRCLNGTKSEPLLCVLVHVLPQVYEYSGSEGQLLIAPPELHPRLRRAAPLLVSLLAGLSIAGSAAISTAALVRGETGLMSLSQQVDADLNNLQSAIDILHSQVESLAEVVLQNCRGLDLLFLSQGGLCTALGESCCFYANQSGVIKDTLQKVQKNLDRSQQERENNTAWYQSMFNWNPWLTTLITGLAGPIIIILLSLIFGPCILNWFLDFVKQRIASVKLMYLRTQYNPLVVTEESTT | **envelope protein [Gibbon ape leukemia virus]**  **Sequence ID: ALV83301.1**  **E value: 1e-79** |
| **ORF88** [1265278 - 1263416] (REVERSE SENSE) YLGYLEEVPLSMVTVDTCYGDLRGIMRLDDLAYEIKPLQDSRRFEHVVFQIVAEPNATGPTFRDDDNETDPLFSEANDSMNPRISNSLYSSHRGNIKGHVQCSNSYYRIYGNITTCSKEVVQMFSLIDSIAQNIDLRYYIYLLTIYNNRDPAPVNEYRIQSAMFTYFKTTFFDTFHVHSSTLLIKYVPHESNYEPERYNFCSRIALLHIGTPGRHYLLVAVIITQTQMRSIGLEYDDNYCTCQRRASCIMQRFPGMTDAFSNCSYGHAQNCFIHSGQCVFETLAPVYNETMTTVRCGNLIVEGREECDCGSFKQCYASYCCQSDCHLTPGSICHIGECCTNCSFSPPGTLCRPIQNICDLPEYCHGTTVTCPANVYTQDGTPCTEEGYCYRGNCTDRNVLCKAIFGVSAEDAPEVCYDINLESYRFGHCIRQQTYLSYQACAGIDKFCGRLQCTNVTHLPRLQERVSFHHSVRGGFQCFGLDEHHATDTTDVGRVIDGTPCVHGNFCNNTQCNVTITSLGYNCHPQKCGHRGVCNNRRNCHCHIGWDPPLCLRRGAGGSVNSGPPPKRTRSVKQSQQSVMYLRVVFGRIYAFIIALLFGTAKNVRTIRTTTVKEGTVTNPE | **none** |
| **ORF89** [1253635 - 1252541] (REVERSE SENSE) PANIIMTGSNSHITILTFNVNGLNAPIKRHRLANWIKSQDPSVCCIQETHLTCRDTHRLKIKAWRKIYQATGKQKKAGVAILVSDKRDFKPTKIKRDEEDHYIMVKGSIQQEELTILNIYASNTGAPRFIKQVLGDLQRDLDSHTIIMGDFNTPLSTLDRSTRQKVNKDIQQLNSALHQADLIDIYRTLHPKSTEYTFFSAPHHTYSKIDHIDGSKALLSKCKRTEIITNCLSEHSAIKLELRIKKLTQNHSTTWKLNNLLLNDYWVHNETKAEIKMFFETSENKDTTYQNLWDTFKAECRGKFIALNAHKRKQEKSKIDTLTSQLKELEKQVQTHSKASRRQDITKIRAELKETETQKNPSKNQ | **none** |
| **ORF90** [1135163 - 1133787] (REVERSE SENSE) GGVPGSMPSLCASDQGVPHTASSPVPSTGHTWPEAGERRAQWLSQSLQGDGTRKTWPVDESRPGSHRAESSGSNEGPSLLREERAAAPGPDSDQEWHRPWLNGSLPTDPPAPGDRVCVPGRAGRHHTQGEATVELCAEPRAGAYCSRRSGHSCSRLLSPAECPSHTQPLGVYLLTPAVQDLWLRDKATFTCFVVGSDLKDAHLTWEVAGKVPTGGVEEGLLERHSNGSQSQHSRLTLPRSLWNAGTSVTCTLNHPSLPPQRLMALREPGEPGSQVGRRGCPQPADPYACPRAMTPAGPQQHRSSSTGKEKGGTSTLAGPTSLPVPPWPEPDSLPHLPAAAQAPVKLSLNLLASSDPPEAASWLLCEVSGFSPPNILLMWLEDQREVNTSGFAPARPPPQPRSTTFWAWSVLRVPAPPSPQPATYTCVVSHEDSRTLLNASRSLEVSCESPPGPGLGRGL | **none** |
| **ORF91** [1043479 - 1042304] (REVERSE SENSE) APWGEAGTRGWEPTGSLRLEDGFPQGPTRAPPPVSWTFSLRAGLVPAQGSSRVTRAGRVLVESTFSGRDMISLQSAHSGRFPFHAGRFLFHAGCFLFHTGCFLFHAGCFLLHSGCFLFHTGCFLFHPGCFLFHAGHFLFHSGCSLFHAGCFLFHAGCFLFHAGHFLFHSACFLFHSGCFLFHGGRFLFHSACFLFHAGCFLFHSGCFLFHVGCFLFHSGCFLFHSGCFLFHAGHFLFHAGCFLLHPGCFLFHAGCFLFDSGCFQFHSGCFLFHAGCFLFHSAQFLFHSGHFLCDTSLGFLSAQSLYPHPVPCYLPPPQSSLPSSSLSLELRPGKVPPDLSPGSPSTGRSLAPALGPPHPAWCQHPPVPREAPFLSLLVVQWPWSPTATRVCP | **none** |
| **ORF92** [1014692 - 1013640] (REVERSE SENSE) NPHTDTHDPTALAQVCATGVRLSALSHLAESWPQHRGQWSRAHSHPAASATPPCGHTRPRSAARPPLLLPPTAPWSILNRPPGDRQPHTPSLAHSRLTQGTAVHTLGPIATPQHPVPPPGRHRHPTRGHTWPTLRLPPCLQRGYCQALPLSHTWAAGSQREMERPSRGVLQSNPPCHPVSSTPQPNTGWMRGLHPPLTHTGTPEQATCPSQTSNPHVHLTPQIQTLPPVMHTKGTAPTKSTRKHGHRAPHEHKAPPCLKTVPAHRHSHTFSFTLTSQPTCTQLWAWSSRKRCEGPRPDLHLLMTRDQQAAHGVGEGSGGDSGQEPEGPGWWAGPGRGRARRQLWTPTGLGS | **none** |
| **ORF93** [1012427 - 1011303] (REVERSE SENSE) DPSAWHPRPPSLTQTFFPFTHPSCHPSTAPIPSPTPPAIYPQPPSLHPPLLPSIHSPHPFTHPSCHLSSAPIPSPTPPAIHPQPPSLHPPLLPSILSPHPFTHPSCHPSTAPIPSPTPPAIYPQPPSLHPPLLPSIHSPHPFTHPSCHLSSAPIPSPTPPAIHPQPPSLHPPLLPSILSPHPFTHPSCHPSTAPIPSPTPPAIYPQPPSLHPPLLPSIHSPHPFTHPSCHLSSAPIPSPTPPATHPKPPSLHPPLLPSTSAPIPSPTPPAIYPQPPSLHPPLLPSILSPHPFTHPSCHLSSAPIPSPTPPAIYPQPPSLHPPLLPSTSAPIPSPTPPAIYPQPPSLHPPLLPSILSPHPFTHPSCHLSSAPISAA | **none** |
| **ORF94** [1012552 - 1011260] (REVERSE SENSE) LYNHQKPRRAQHPAQDTAESASSPTPAHGLTPQPRSDPSSPEIQVPGIPVPQASPRPSFPSPTPPATHPQPPSLHPPLLPSILSPHPFTHPSCHPSTAPIPSPTPPAIYPQPPSLHPPLLPSIHSPHPFTHPSCHLSSAPIPSPTPPAIHPQPPSLHPPLLPSILSPHPFTHPSCHPSTAPIPSPTPPAIYPQPPSLHPPLLPSIHSPHPFTHPSCHLSSAPIPSPTPPAIHPQPPSLHPPLLPSILSPHPFTHPSCHPSTAPIPSPTPPAIYPQPPSLHPPLLPPIQSPHPFTHPSCHPPQPPSLHPPLLPSILSPHPFTHPSCHLSSAPIPSPTPPAIYPQPPSLHPPLLPSIHSPHPFTHPSCHPPQPPSLHPPLLPSILSPHPFTHPSCHLSSAPIPSPTPPAIYPQPPFLQPEGTGKPSEARPQDG | **none** |
| **ORF95** [1012692 - 1010686] (REVERSE SENSE) RRRENHQSQPPPENRVCPFNRVIEEGWLLAVLSSSQAPGSPGHSHSDCTITKSQGGPSTQPRTQLSLRPAQHQPTASLPSLGLTLLALRSKCLASPSPKPHPDLLSLHPPLLPPIHSPHPFTHPSCHLSSAPIPSPTPPAIHPQPPSLHPPLLPSILSPHPFTHPSCHPSTAPIPSPTPPAIYPQPPSLHPPLLPSIHSPHPFTHPSCHLSSAPIPSPTPPAIHPQPPSLHPPLLPSILSPHPFTHPSCHPSTAPIPSPTPPAIYPQPPSLHPPLLPSIHSPHPFTHPSCHLSSAPIPSPTPPAIHPQPPSLHPPLLPSILSPHPFTHPSCHPSKAPIPSPTPPAIHLSPHPFTHPSCHLSSAPIPSPTPPAIYPQPPSLHPPLLPSILSPHPFTHPSCHLSTAPIPSPTPPAIHLSPHPFTHPSCHLSSAPIPSPTPPAIYPQPPSLHPPLLPSILSPHFCSLKAQGSPLRPGHRTVEGFGPLEWVEGLPQPEKGFPAGTAAADLVPMTSSSVWLQQSYFFLSEFLSKDPFHKPHVSGGLCRRCVWPWSGWWEAGPLRTCRRSQRGRKGRGHSGVLRAKVPKQWFPSITPQHPRDSPAQRCHTWSAGLQPRLMPPSQVKSKQQSTSTPLWAKPEEAHAVVSTGGPKNAAAQSPRWRLVGAPRTTGHTP | **none** |
| **ORF96** [1009219 - 1007783] (REVERSE SENSE) SHILAPGAIECGVICQKGLCRSHQDKDFEVRAPWIVKDFEVKEPWIVKDFEVKEPWIVKDFEVRAPWIVKDFEVKEPWIVKDFEVRAPWIVKDFEVKEPWIVKDFEVKEPWIVKDFKVRAPWIVKDFEVRETWIVKDFEVREPWIVKDFEMREPWIVKDFEVRAPWIVKDFEVKEPWIVKDFEVRAPWIVKDFEVKEPWIVKDFEVRAPWIVKDFEVKEPWIVKDFEVKEPWIVKDFEVRAPWIVKDFEVRETWIVKDFEVRETWIVKDFEMREPWIVKDFEVRAPWIVKDFEVRAPWIVKDFEVREPWIVKDFEVKEPWIVKDFEVRAPWIVKDFEVRETWIVKDFEVRETWIVKDFEMREPWIVKDFEVRAPWIVKDFEVRAPWIVKDFEVREPWIVKDFEVREPWIVKDFEVRAPWIVKDFEVRAPWIVKDFEVREPWIVKDFEVRAPWIVKDFKVRAPWIVKDFKVKDTLDYRGG | **none** |
| **ORF97** [1009142 - 1007577] (REVERSE SENSE) GFRGEGTLDCQGFRGEGTLDCQGFRGEGTLDCQGFRGEGALDCQGFRGEGTLDCQGFRGEGTLDCQGFRGEGTLDCQGFRGEGTLDCQGFQGEGTLDCQGFRGEGNLDCQGFRGEGTLDCQGFRDEGTLDCQGFRGEGTLDCQGFRGEGTLDCQGFRGEGALDCQGFRGEGTLDCQGFRGEGTLDCQGFRGEGTLDCQGFRGEGTLDCQGFRGEGTLDCQGFRGEGNLDCQGFRGEGNLDCQGFRDEGTLDCQGFRGEGTLDCQGFRGEGTLDCQGFRGEGTLDCQGFRGEGTLDCQGFRGEGTLDCQGFRGEGNLDCQGFRGEGNLDCQGFRDEGTLDCQGFRGEGTLDCQGFRGEGTLDCQGFRGEGTLDCQGFRGEGTLDCQGFRGEGTLDCQGFRGEGTLDCQGFRGEGTLDCQGFRGEGTLDCQGFQGEGALDCQGFQGEGHPGLSRGLGPRTSVLAGDRRGDTGMGRGRGRQGWCYGLRVCVCPKLLCDSRHPRVRACRAGASGRWLGPEGEPSKM | **none** |
| **ORF98** [989725 - 988619] (REVERSE SENSE) TCCHLWDQDPGAAPRLAGRAGEPTLAGPPANPHPRSPKEAERRWPWWPGHRQPDWYRAGLVQARTSPTLMAWWSPLYRENTEAQQGQGHGLRLHGDGGWVSGPGGRGGCSTRTAWAVASLLPALSHPSPSEPPAPSFPLSLSLATLLSPCLSASLKVSPCPNLCLCPIVGVSDSSLSSPSAGLCPAILPVLASLSFCVSPSVPPFSIHLSPVSVSIPPSLFPSIPPSVPVPLLCPFLCLCLSVCLFVSLCVSLCLSASISVFLYLCLYLSLFVSLCVCLSLLCLSMPLSISLSLCLCISVCLFVSLCMSLSVSFCISVYICLSHLSLPVCVSLSLCLCLSLSVSSSLSLPLCHFVCVSLCLSVCVSSSL | **none** |
| **ORF99** [989207 - 988029] (REVERSE SENSE) LISVKPVCWALSRHLACLGLSLFLCVSVGASFLHPSLPRVCLYPSVSLPVYPSLCACPFAVSLSLPLSLCLSLRVPVCLSLSLCFYLCLSVSLSISQPLCVPVCVSLSSLSFHASVYISVSLPLYLCLSVRVPVYVSLGLFLYLCLYLSLSSVSSCVCLSVSLPLSLSLCLFISVSASVSFCVCFSMSLCLCLFLSVALPLSVSPYTSLSLSLFLSLSLFVFLCLSVSVSSPLYISVFLLLSVSVSLQPSLSLFVSVFLCLSNSLSLSLCLYLSLSSYLCLYLSLSLTLCICLSLISIALSLSLSLSSLSLSLSLFLSLCISLYLCLCNSLYLLLPLFLSLCLCLSLSLSWSLSLCNFISVSLFLSLFFSVCLCLYLCLSLSVSLSLCISVSL | **none** |
| **ORF100** [988137 - 986932] (REVERSE SENSE) LYLCLSVSISVFLCLSLPISLSLFICLSVSVYLCLSVTLSLSFSVSVSVCLSLCLCFSLSLSLPIFLSLSLSMSLSGSVFLSLCLCLSISVSIYLSLCNSSLSLFVSACLFLCLCFSLCFCLFVSLSLCLSLCLCLYLCVSISLSLSVSVSLFLCYSVSVSVSLSLCNSSSLSLFVSACLFLCLCFSLSLSLCFSVSVSVSLSLSVSLCLYFSVSLSLSVSVSLSFSVTLSLSLCVSLCLSVFLCYSVSVSLCLSLLLCLCLCVSLSVFLCYSVSVSVSLSVSLSFSVTLSLSLCLSLSLFLCYSVSVSLCLSVFLCYSVSVSVSLSLCLSLLLCLCLCVSLCLSFSVTLSLSLSVSLSFSVTLSLSLCLCLSVFLCYSVSVSVSLSVSVFLRYSVSLSVSL | **none** |
| **ORF101** [988025 - 986751] (REVERSE SENSE) LYLCLFLSLCLCVCLCVSVFLSLSLCLFFSLCLCQCLFLDLCFFLSVSVSPSLSLSICLSVTHLCLYLCLHVSFCVSVSLSVSVSLFLCLCVFLSVSVCISVFLFLCLSLSLSLSFSVTLFLSLCLCLSVTPHLCLYLCLHVSFCVSVSLCLCLSVSLSLSLCLCLCLYLCVSISLSLFLSLSLSLCLSLLLCLCLCVSLSASLSFSVTLSLSLSVSLCYSVSVSVSLCLSFSVTLSLSLCLSLSLCLSLLLCLCLCVSLCLSFSVTLSLSLSVSLSFSVTLSLSLCLCLSVFLCYSVSVSVSLSVSLSLLLCLCLSLSLCLSLLLCLCLCVSVSLSFSVTLSLSLCLSLSLSFSVTLSLSLSLCDSICYSVSVSVSLCLSFSVTLSVSLCLSLVCLSLLLCLCLCVSVSLHCFPGEGEAMYLPA | **none** |
| **ORF102** [977909 - 976695] (REVERSE SENSE) ASQTQGGTGVRGVHEQPRGSPATPTGPRSILRHPPGAGGFLLGTRGPPFCTETHVQKGTPQGHTQTHPARLGHPAHARHTTNTGAGTRLAPGQDSSRQLGQCTQPLTHTTHPHKPSHPHIPTHPHRSSHSHTSTHILKHPTHPHTPHTPHTSTHTLTSPHPHTPHTPHTPTHPHTPSHPHTPPHIHTHPTPHTHPHTLTHPHTPTHPHTPSHPHTSTHTPHTHTHPHIPTPSHILTLPHIPTHPHTPHPHTSTHILTHIHTPSHTTHPLPHTPSHTPHIHTPSHSHTFTHPHTPHTPHVHTHPHIPTHPHTPHTPHTPHILTSTHTPHTKHTLTHPHTPTHPHTPHTPHIHTHPHIHTHAHTPHTSHTSTHTLTPSHSLTPPHSHTPPLSHPHSHTLTHPHTLTV | **none** |
| **ORF103** [977865 - 976537] (REVERSE SENSE) AAEGQPCHAHRPQVYSQAPAWGWGLPSGNSRTSVLHGDTRPERDTPGPHPDTPSPTRAPSTRQAHNKHRSWHTPGPRPGQQQATRPVHTAAHTHHTSPQALTPSHPHTPSQILTLPHIHTHPQTPHTSTHTPHTPHIHTHPHIPTPSHTPHPTHPHTSTHTLTSPHTPTHPHTPHTPHTPSHPHTSSHSHTSTHTLTSPHIHTHPTHPHTPSHPHTLTYPHTPTHPHTSPHPTSTHIHTHPHTHTHTLTHHTPTPSHTLTHPTHPHTLTLSHLHTSTHTPHPTRAHTPSHPHTSTHTPHPTHPTHPHIHTHPTHQTHPHTSSHSHTSTHTPHPTHPHTPSHPHTCTHTPHIPHIHTHPHTLTLPHTPTLSHPPTLTPSLTHSHTPSHPHSLKTSHTLTPSHTHTPSHTVTHSTHTLTHSLTHTLTPSLSHTHTLSHTLTHTHP | **none** |
| **ORF104** [978775 - 976346] (REVERSE SENSE) RHTIHTDTLTHTQTPRHTYRDRHTQTHRNTSTHTTHTRHTDTHRHTQRQRHTQTQRHTNTDIPHTHTHGHTYKDRDTHKHTDTQAQTHYHTHTGTQTHRHIYRDRDTHRHRNTSTDTLPHTQRHTDTHRHTQSQRHTDTETQAQTHYHTQTRIGTHRDRDTRRHRYTSTDTLPHAETHTEIDTDTHKVRGTHRHIQTHRGTRHRHTHSEAHTQPQPRAHSSHGPHSGPQGLTASQRPPVPQPDKHRPCSRSHTDAHILPPAAKLTLTHAHVALLGQALAHGVREGSESEHHRHRVGLVSGGSMSSRGAALPRPQAPGLFSGTRLGLGASFWELEDLRSARRHTSRKGHPRATPRHTQPDSGTQHTPGTQQTQELAHAWPQARTAAGNSASAHSRSHTPHIPTSPHTLTSPHTLTDPHTPTHPHTSSNTPHIHTHPTHPTHPHTPSHPHTLTHPTPHTPPHIHTHPHIPTHPHTSTHTPHPTHTLTPSHILTLPHIHTHPHIPTHPHTPHTPTHTLTSPHPHISSHSHTSPHIPTPHIHTHPHTSSHTYTHPHTPHTHSLTHPHTPHTSTHPHTLTPSHIHTHPTPHTCTHTLTSPHIHTHPTPHTPHTSSHPHTPHTPNTPSHILTLPHIHTHPTPHTSTHTLTSTHMHTHPTHPTHPHTPSHPHTPSHPHTLTPPHSHTLTHTLSHTLTPSQSENFTHPHTLTHPHTLTHRHTLHTYPHTLSHTHPYTLTLTHPHTLTHPHTYTPLTACLQSVRGVPQRATTPCGSGAQNPPSSGSGPFPSQLMTFGYHGSGVPLCSPAANPCISLGPC | **none** |
| **ORF105** [974211 - 972364] (REVERSE SENSE) RPSEWAGMGVVAHPSGVDVGAQQEKQTRPHRRLQMEASEAAPGLPQTPPLHVPPCPGSRPSDPQALPRTSQLLHSPGFSPPAPMEPPPWAVWACLAGRVGSPRPVAQAPISVSSAQRTRTFHTRASGEDPQAPPAQPRRPETGPAAAAAEALLPPCGRQQHRTPPTTRRFPGSGQGQGQGHGQGQGQGQGQGQGQGQGQGQACSRGRIPALPWAQGWGPVRVQHCDPLPSPAPAAEVAVASANRHQLVGLCGLGSQPLPKSCIPWVWGRCDLGTLWPGGLARLHLGQMGKLAWAPRWGPAPPTSAGSIRCSLTLVRTLPPGVAQCPRGSPRTRPAREPARAWGLRVFTMVANGPTGCCMRRTSLRHQPPATVPGGQGTRLRSRGSSPGDALPAAGCGAGGCARKWVGGRSPPEHRGQQPGPGPGPAGPQGLQSPREEEGLRVLRISSHPGDEPQRAGTRKGRPRPNIGVWALVGQGRLSLGAGQGPVGVRVPLPGPRLQPQPLNPSRDPRDRLGTPWWPSCATAVLLLRGSRGSRGSRGPWPGSLLGSQAGALCRPLMRGQRTSEASLKDAPRSGSQEPSSHTNQKSPKEAETRISETRNYRIRTQTKAKCRRYQT | **none** |
| **ORF106** [944710 - 943490] (REVERSE SENSE) APWGEAGTRGWEPTGSLRLEDGFPQGPTRAPPPVSWTFSLRAGLVPAQGSSRVTRAGRVLVESTFSGRDMISLQSAHSGRFPFHAGRFLFHAGCFLFHAGCFLFHAGCFLLHSGCFLFHTGCFLFHPGCFLFHAGHFLFHSGCSLFHAGCFLFHAGCFLFHAGHFLFHSACFLFHSGCFLFHTGRFLLHAGRFLFHAGCFPLHSGCFPFHAGRFLFHAGRFLFHSGCFLFHAGCFLFHAGHFLFRAGHFLLHTGRFLLQAGRFLFHAGCFLLHSGCFPFHAGRFLFHAGRFLFHSGCFLFHAGRFPFHSGCFLFHAGHFLFHSGCFLFHAGCFLFHAGCFLFHAGRFLFHSGCFLLHAGCFLFHAGRFLFHSGCFLFHAGCFLLHSGCFLFHAGHFLFHSGCFLLHS | **none** |
| **ORF107** [944499 - 942973] (REVERSE SENSE) FPFKVPILDASRSMLDASSSTLDASCSTLDASCSTLDVSCYTLDASCSTLDASCSILDASCSMLDISCSTLDAPCSMLDASCSMLDASCSMLDISCSTLHASCSTLDASCSTLDASCSTLDASCSMLDASRYILDASRSMLDASCSTLDVSCSTLDASCSTLDASFSTLDTSCSALDTSCSTLDASCSRLDASCSMLDASCYILDASRSMLDASCSTLDVSCSTLDASCSTLDASHSTLDASCSMLDISCSTLDASCSMLDASCSMLDASCSMLDVSCSTLDASCYMLDASCSMLDVSCSTLDASCSMLDASCYILDASCSMLDISCSTLDASCYILDASCSMLDISCSTLDASCYILDASCSMLDISCSTLDASCYILDASCSMLDISCSTLDASCYILDASCSMLDISCSTLDASCYILDASCSTLDASHSTLDASFSMLDLSCSTLDASCSMLDASFSIPDTSYSILDTSCATPPRAFGLPSPSGLIPSPLTSHFHVRPSSAPPSL | **none** |
| **ORF108** [944513 - 942945] (REVERSE SENSE) WEGHDFPSKCPFWTLPVPCWTLPLPRWMLPVPRWMLPVPRWMFPVTLWMLPVPHWMLPVPSWMLPVPCWTFPVPLWMLPVPCWMLPVPCWMLPVPCWTFPVPLCMLPVPLWMLPVPHWTLPAPRWTLPVPCWMLPVTFWMLPVPCWTLPVPRWTFLVPLWMLPVPRWMLPFPRWTLPVPRWTLPAPHWTLPAPGWTLPVPCWMLPVTFWMLPVPCWTLPVPRWTFLVPLWMLPVPRWTLPIPLWMLPVPCWTFLVPLWMLPVPCWMLPVPCWMLPVPCWTFLVPLWMLPVTCWMLPVPCWTFLVPLWMLPVPCWMLPVTFWMLPVPCWTFPVPLWMLPVTFLMLPVPCWTFPVPLWMLPVTFWMLPVPCWTFPVPLWMLPVTFWMLPVPCWTFPVPLWMLPVTFLMLPVPCWTFPVPLWMLPVTFWMLPVPLWTLPIPLWMLPFPCWTFLVPLWMLPVPCWMLPFPFRTLPIPFWTLPVRHLLGLLVCPVPLASYHPPLPPTSTFVLPQLLPLSRASAWQGPS | **none** |
| **ORF109** [892964 - 891909] (REVERSE SENSE) NPHTDTHDPTALAQVCATGVRLSALSRLAESWPQHRGRWSRAHSHPAASATLPCGHTRPRSAARPPLLLPPTAPWSILNRPPGDRQPHTPSLAHSRLTQGTAVHTLGPIATPQHPVPPPGRHRHPTRGHTWPTLRLSRCLQRGYCQALPLSHTWAAGSQREMERPSRGVLQSNPPCHPVSSSTPQPNTGWMRGLHPPLTHTGTPEQATCPSQTSNPHVHLTPQIQTLPPVMHTKGTAPTKSTRKHGHRAPHEHKAPPCLKTVPAHRHSHTFSFTLTSQPTCTQLWAWSSRKRCEGPRRDLHLLMTRDQQAAHGVGEGSGGHPGQEPEGPGWWAGPGRGRARRQLWTPTGLGS | **none** |
| **ORF110** [891010 - 889775] (REVERSE SENSE) WPVNLSPEGPAQPQGYRRRENHQSQPPPENRVCPFNRVIEEGWLLAVLSSSQAPGSPGHSHSDCTITKSQGGPGTQPRTQLSLRPAQHQPTASLPSLGLTLLALRSKCLASPSPKPHPDLLSLHPPLLPPIHSPHPFTHPSCHLSSAPIPSPTPPAIHPQPPSLHPPLLPSILSPHPFTHPSCHPPEPPSLHPPLLPSTCSPHFCSLKAQGSPLRPGHRTVEGFGPLEWVEGLPQPEKGFPAGTAAADLVPMTSSSLWLQQSYFFLSGFLSKDPFHKPHVSGGLCGRCVWPWSGWREAGPLRTCRRSQRGRKGRGHSGVLRAKVPKQWFPSITPQHPRDSPARPCHTWSAGLQPRLMPPSQVKSKQQSTSTPLWAKPEEAHAVVSTGGPKNAAAQSPRWRLMGAPRTTGHTP | **none** |
| **ORF111** [887825 - 886806] (REVERSE SENSE) VRAPWIVKDFEVKEPWIVKDFEVRAPWIVKDFEVRAPWIVKDFEVREPWIVKDFEVREPWIVKDFEVKEPWIVKDFEVRAPWIVKDFEVREPWIVKDFEVRAPWIVKDFEVRAPWIVKDFEVKEPWIVKDFEVRAPWIVKDFEVKESWIVKDFEVRAPWIVKDFEVREPWIVKDFEVRAPWIVKDFEVRAPWIVKDFEVRAPWIVKDFEVKEPWIVKDFEVKEPWIVKDFEVKEPWIVKDFEVRAPWIVKDFEVRAPWIVKDFEVREPWIVKDFEVRAPWIVKDFEVRAPWIVKDFEVRAPWIVKDFEVRAPWIVKDFEVRAPWIVKDFEVKDTLDYRGG | **none** |
| **ORF112** [887628 - 886600] (REVERSE SENSE) GEGTLDCQGFRGEGALDCQGFRGEGTLDCQGFRGEGALDCQGFRGEGALDCQGFRGEGTLDCQGFRGQGTLDCQGFRGEGILDCQGFRGEGALDCQGFRGEGTLDCQGFRGEGALDCQGFRGEGALDCQGFRGEGALDCQGFRGEGTLDCQGFRGEGTLDCQGFRGEGTLDCQGFRGEGTLDCQGFRGEGALDCQGFRGEGTLDCQGFRGEGTLDCQGFRGEGTLDCQGFRGEGALDCQGFRGEGALDCQGFRGEGALDCQGFRGEGHPGLSRGLGPRTSVLAGDRRGDTGMGRGRGRQGWCYGLRVCVCPKLLCDSRHPRVRACRAGASGRWLGPEGEHSKM | **none** |
| **ORF113** [861960 - 860740] (REVERSE SENSE) ANPPALRAPHRPSPDTQTRRHAQTHTHTHRHTQTCTHRNAQTHTDMHRDTHTETCTDMHRDTHRHAQRHTQKCTDTHGHAQTHTYVHRDTHIDTHPHGCTDTETHTGKHIHTQTHKDTDTRICTHTCTWMHRHTLTHIETHTHRHIKTHTDMPAHRHPLPQVTLAAPGHCPAPCHEPASPVHTTHLSTPHTCPHYTPVHTTHLSTLHPIHPTHLPTPHTCPHRTPVHTTHLSTLHPVHPTHLSTPHTCPHYTPSTPHTCPHHTPVHTTPIHPTSLSTPHTCPYHTPVHPAHLSTPHTCPPCTPVHTAHLSTLHPIHPTHLPTIHTCPHRTPVRTTHLSTPDTCPHYTPSTPHTCPHHTPVHPTHLSTPHTCPHYTPVRITHNLTPPAHPPLTAHTLASHTLMLTLMH | **none** |
| **ORF114** [861433 - 860237] (REVERSE SENSE) TCLPCPHHTPVHPTHLSTLHPCPHYTPVHTTPHPPHTPAHTTHLSTPHTCPHHTPVHTTPRPPHTPVHTTHLSTLHPVHPTHLSTPHTCPHYTHPPHIPVHPTHLSIPHTCPPRTPVHPAHLSTLHTCPHCTPVHTTPHPPHTPAHNTHLSTPHTCPHHTPVHTRHLSTLHPVHPTHLSTPHTCPPHTPVHTTHLSTLHTCPHHAQPHTSSSSTTHCTHACISHAHAHTHALTHTFSHLLPSSHSHMHTWRCWARLGHMGSGKARRAYALASLAACPGLRGRGHSCCPTTPHVPLQGLGMAPRRARAQGQLVSAPRGPRPEDTWASQCCGLQRVCVHTHAHICVVCACVHGRGSLGGPGSASGAGVGGLGPSAFLQPSLTSPPLSALRGHQPALGLAPA | **none** |
| **ORF115** [861521 - 859938] (REVERSE SENSE) RHTQTCPHTGTPSRRSRLQPLATVLPRVMNLPPLSTPHTCPPHTPVHTTPLSTLHTCPHYTPSTPHTCPHHTPVHTAHLSTPHTCPHYTPSTPHTCPHHTPVHTTPRPPHTPVHTTHLSTLHPSTPHPCPPHTPVHTTHLSTPHTCPPRTPVHPAHLSTLHTCPHYTPSTPHTCPQYTPVHTAHLSAPHTCPHQTPVHTTPRPPHTPVHTTHLSTPHTCPHHTPVHTTHLSASRTTSHLQLIHHSLHTRLHLTRSCSHSCTNTHILPPVAKLTLTHAHMALLGQAWAHGVREGSESVRLGLSGGMSGSAGQRSQLLPHDTACAPAGPGNGTPQSPRSGPARVSTAWTTSRGHLGQPVLRAAASVCAHACPYMCCVCLCAWEGVPGWARVCLGGWSGRPGPLSFPPTQPHFPAPLSSAWPPASPGLGPGMRPEAGPWITLRTGTWAFWASSPRTRLGLSASHSSGVMPWGPARTLAAPLRRLDTLAGGGAAAPPPPPPSSLKNKTSWAPGRSSHPHPTTQAQTHSCKYF | **none** |
| **ORF116** [858608 - 857370] (REVERSE SENSE) THRGTRHRHTHSEAHTQPQPRAHSSHGPHSGPQGLTASQRPPVPQPDKHRPCSRSHTDAHILPPAAKLTLTHAHVALLGQALAHGVREGSESEHHRHRVGLVSGGSMRSRGAALPRPQAPGLFSGTRLGLGASFWEHEDLRSARRHTSRKGHPRATPRHTQPDSGTQHTPGTQQTQELAHAWPQARTAAGNSASAHSRSHTPHIPTSPHTLTSPHSHTSTHILKHPTHPHTPHTPHTSSHPHTPSHPTPHTPHTSTHTLTSPHTPTHPHTPHPTHTLTPSHSHTLSHPHTSTHTPHIHTHPHIHTHPHIPTPSHILTLPHIPTHPTPHIHTHPHTHTHTLTHHTPTPSHTLTHPTHPHTLTLTPSHIHTHPTHPTHPHTPHTPHVHTHPHTLTHPHTPHTLTHPHTPTHPHTF | **none** |
| **ORF117** [858286 - 856868] (REVERSE SENSE) GAEGQPCHAHRPQVYSQAPAWGWGLPSGNTRTSVLHGDTRPERDTPGPHPDTPSPTRAPSTRRAHNKHRSWHTPGPRPGQQQATRPVHTAAHTHHTSPQALTPLHPHTPTHPHTSSNTPHIHTHPTHPTHPHIPTHPHTPHPTHPTHPHTPSHPHTPPHIHTPHTPHTPSHPHTHTHSHIPTHPHTPHTSTHTLTSTHTLTSPHPHISSHSHTSPHTPHPTSTHILTHIHTPSHTTHPLPHTPSHTPHIHTPSHSHLHTSTHTPHTPHIHTHPTPHTCTHTLTPSHIHTPHTPSHILTLPHIHTHSEAPHTSTHTPHTPHIHTHPTHTHTSSHSHTSTHTPHTPHIHTHPTHPHTPSHPHTSTHTLTSPHTLTHPHTPTHPHTPHIPHTPTHPHTSHTSSHTYTHPHTPHTHTPSHPHTPHIHNPHTLTPSYTHPHTHSHFTHPHTSTHPHIHTHPHSHTPLHPHTPTFSHTC | **none** |
| **ORF118** [858330 - 856438] (REVERSE SENSE) ASQTQGGTGVRGVHEEPRGSPATPTGPRSILRHPPGAGGFLLGTRGPPFCTETHVQKGTPQGHTQTHPARLGHPAHAGHTTNTGAGTRLAPGQDSSRQLGQCTQPLTHTTHPHKPSHPYIPTLPHIHTHPQTPHTSTHTPHTPHILTSPHTLTPHTPHTPHIHTHPHIPTHPHTSTHPTPHTHPHTLTLTHTLTSPHIHTHPTHPHTPSHPHTPSHPHTLTYPHTPTHPHTPHTPHPHTSSHTYTHPHTPHTHSLTHPHTPHTSTHPHTHTFTHPHTPHTPHTSTHTPHPTRAHTPSHPHTSTHPTHPHTSSHSHTSTHILKHPTHPHTPHTPHTSTHTPHTPTHPHTPTHPHTPHTPHTSTHTPHIHTHPHIPTHPHTPSHPHTPSHILTLPHIHTPHTSLTLPHIHTHPTHPHTHTHTLTHHTPTLPHTLTHPTSTTLTPSHPHTHTHTHILTSHTHTHPHTHTSIHTHILTHPYTLIHPHSHTHADTIPTSYIPHSHPPTHSHNPHTHPTLPHISTLPHLHKHLQALTPSHSHAPLTLISSHTHPHTSPHPYTLSHTLTPSHPYTPSHALIHTLTHPHTLTHHTLTSHAFSLTLTHTPSCALTPTHPLTLTHPHPHTPSHPHMPTHTP | **none** |
| **ORF119** [853508 - 852216] (REVERSE SENSE) PAVWGPPGPWPKHPSPCPAPSALGRFTRGPLGRTPRHRQPNPGGRKLDPLRQPLRLCCPRVADSSIERRRPRGASRARGRGRGTGRGTGRGRGRGRGRGRGALGAGSRLCLGLRAGARSVSSTVIPSPPQRLPRRWLWHPRTGVSLWGSVVWDPSLCQSPAFPGCGDAVTWGRCDLGTLWPGGLAGLHLGQMGKLAWAPRWGPAPPTSAGSIRCSLTLVRTLPPGVAQCPRRSPRTRPAREPARAWGLRVFTMVANGPTGCCMRRTSLRHQPPATVPGGQGTRLRSRGSSPGDALPAAGCGAGGCARKWVGGRSLLSTEDSSQAQAQALQAPRGSRAPERRKASVCSQSAHTPGRNLRGSGQGKEGPGPTSGSGRWWDRAGSALGPVRALLGFGSPCQVRVCSHSPSTPAGTPETGSGPPGGRLVPLQFCC | **none** |
| **ORF120** [852984 - 851950] (REVERSE SENSE) PGDAVAWGPCRAAPGADGEAGLGSSVGPRAPHFCRQHPMLPDPGQDPSSWRGPVSPQKPQNAACPGAGSGLGPAGLHYGREWTHGMLHEAHELEAPASGHCPWRTRDAAPEQRVLPWGCPPGGWVWGWGLCPEVGGWEKPPEHRGQQPGPGPGPAGPQGLQSPREEEGLRVLTISSHPGEEPQRVGTRKGRPRPNIGVWALVGQGRLSLGAGQGPVGVRVPLPGPRLQPQPLNPSRDPRDRLGTPWWPSCATAVLLLRGSRGSRGSRGPWPGSLLGSQAGALCRPLMRGQRTSEASLKDAPRSGSQEPSSHTNQKSPKEAETRISETRNYRIRTQTKAKCRRYQT | **none** |
| **ORF121** [822344 - 821289] (REVERSE SENSE) SPAWSGTRGAGPGAAAPWSAAGPRPGRARPPRLAGRGPAALSQRAWRDTARRARNLGRPEETRSGCGLERPPPTPPAPRTPRASHQARSPRRGPEASRTPAPMREPAAANHSEASRGRCCLARAPGPRSRRQEGNGTDAFSARRARSRSPGGFVTAGPAPPSLLAPAARTPRPRCWPQAVSAEFREYPLPAAALGREAGLPLRSWSTQGGLGPPRLSDPGPRATQVPMHPSHKPLRASPPLPGSPHAPPPCTGRLGLPSCPPSWARGHCLPCSPWLRIALARCCPPPSAPAEPPCFAPLRQGDACGCPRISGSPSTKARRASTGPCTSVTHLALVLMAVLSESPHLGLCTPG | **none** |
| **ORF122** [823126 - 821081] (REVERSE SENSE) RRRREVAHLAAVLQEGAGLGGERHHVFADEDGRDTAVPEQRHVGGARQHGHHIDGGEAEHRVQHHLLRRYVLALHADLAERHARQHQQQHVAVEEGEALGPQRQPGARRLPQAGLHVQQVQQVQRHPKQAAAQVLALAEGAHVLHAVQRHEQQEQAGHTHRQQQRQRLARHGVRRAAPRRLVVLEDEVELDLQHEDVKEPQDHGVAALGRAHLGAHPHGHVAQHQQEAHVAHQHDDAHADLARALVLLHQVRVHDGHAAHDHQRGQAHVAPVRGRQHHGRPPGPGLGARGPHAWRDEALRRSASGHGATRPGELETWGDRRRQDQGAGWSGLPQHPPPPAPRVLPTRRGLRAGAPRHRARLRPCANRRPRTTARPPEGAAASPEHPGLVPAARKETERTRFLPAELVPGARGGLSLLGPRRPPSWLRRPGPHARAAGLRPFPRSSGNTLSPPPHWAGRRACPSALGVPKEASALPGFLIPDREPPRSRCIRPTSLSEPPRPCPAAPTPLLPARAGSVSPPVHHPGRVATACPAPHGSALPWRGVVPHPLPRQSHPALLLCARAMPVAALESREARPPRPVGLPQAPAPLSHTWHWSSWPCCRSPRTWACAHLGDNGGPHTRTHPKVTDRLTPSQPLTPSTAPGLGLRLLPCLDKGIKFNVPVTVGRSDCPGDTAGPTDPGKR | **none** |
| **ORF123** [820752 - 819670] (REVERSE SENSE) RGSHGAAHPFRPCLQPLRPTTLSLDPQSLGRCGPRGSALAKGRTPAPSGAPTPASQSPARPPLRRDLAPGPPVLGLLGPGRGPSSPHAPRLRPGTDSGASRPFTARAPQGPSPHNHPSEPPGGGAGGGCCAPAAGRCRAWAAPSRLRLCLSPRGFPTQGRVPVTPPAPGLTPAASAPAPPHSPHGPEVQGRPGPPPPPRTQPRTQPVPPAPAPTPPPADAAPLPGPPAQPLPPLPGPRRLLPPRPSPARAAPPPTQRPRPAPLGPRAPPDLGTWERGGAGAPPPPQRRLPRRQHRAAARRTGGSGERGALAHPQARSPVSTLLTHRPESQGPAQCPPRPETPLPPPRATPLSRTGAHRPRS | **none** |
| **ORF124** [820811 - 819462] (REVERSE SENSE) LQPQPENFLPLRAWRGQINSGGATAPLTLSGRASSPSAPLRCRSILSHLADAGRGALRWRRDGLQPLAEPLPRQARARPGHLSVGTWPQAHLSWVSWAPAEAPPPPTPRGSDLAQTLGPPGPSLPGPRKDPRPITTPRSPREEGREGAAVLPPRAGAELGPLPAGCVSACPPEASPRRAGSPSPRRPQASPLPHRPPPHPTVPTAQRCRVAQARRPLRGRSRAPSQSRPRPRLPRRPQMLRRSRARPPSLCRHCRGRAGSSRRAPPPPARHRPRPRGPAPRPSAREPRRTWGPGSAGVPVPRPLRSAGCHGDSTARRRAGLVALGSGAPSRTPRRGVPSPPSSPTAQNPRVPPSAPRALKLRCRPLERPHSAGQAPTGPGADRDVGAGLAWPPGPSAPPSVLFGEKPSLPRFLLRAEERNDERPPPSSPARPGGKLGFGDLGFILSLRWH | **none** |
| **ORF125** [753793 - 752627] (REVERSE SENSE) PQCRPSRDSPQAHRSPGARDQRPRAARTPPRHTTPQVPGTRGHGQHGLPPGTPLPRCQGPGAMGSTDFPQAHSSPGARDQGPWAARTSPTHTAPQVPGTRGHGQHGLPPGTQLPRCQGPGAMGSTDFPQAHSSPGARDQGPRAARTPPRHTAPQAPGTRGHGQHGLPPGTQLPRDQGPGATGSTDSPQAHSSPGTRDQGPWAARTAPRHTAPQAPGTRGHGQHGLPPGTQLPRDQGPGATGSADSPQAHSSPGARDQGPWAARTAPRHTAPQAPGTRGHGQHGLPPGTQLPRDQGPGAMGSTDSPQAHSSPGARDQGPWAARTPPRHTAPQGPGTRGHGQHGLPPGTQLPRDQGPGAMGSTDCPQAHSSPGARDQGPWAARTPPRHTAP | **none** |
| **ORF126** [753986 - 752421] (REVERSE SENSE) EEGRSHSGPWATPGLTCPALGPLTRVMSREAAAALMCRLGCRTELLQSRARAKVPTELGKGDALTTVSAKQGLPTGTPLPRRQGPAATGSTDSPKAHHSPGARDQGPWAARTSPRHTTPQVPGTRGHGQHGLPPGTQLPRCQGPGAMGSTDFPHAHRSPGARDQGPWAARTSPRHTAPQVPGTRGHGQHGLPPGTQLPRRQGPGATGSTDSPQAHSSPGARDQGPWAARTPPRHTAPQGPGTRGHGQHGLPPGTQLPRDQGPGAMGSTDCPQAHSSPGARDQGPWAARTPPRHTAPQGPGTRGHGQRGLPPGTQLPRCQGPGAMGSTDCPQAHSSPGARDQGPRAARTPPRHTAPQGPGTRGHGQHGLPPGTQLPRRQGPGAMGSTDSPQAHSSPGTRDQGPRAARTPPRHTAPQGPGTRGHGQHGLPPGTQLPRRQGPGAMGSTDSPQAHSSLGTRDQGPRAARTPPRHTAPQVPGTRGHGQHGLPPGTQLPRDQGPGATGSADSPQAHSSPGARDQGQTA | **none** |
| **ORF127** [754254 - 752314] (REVERSE SENSE) MFLWRVSMAWNCGRRQQGHPGQGGFSCTCGTVPPPSVPQGSPTAGPAAQPSLHPQHSLAEFGVDRKGQSRWDSTSAGVLNTWGRCWALVGRRAQPLWAMGHPRSHLSRPGPTYQGDVSGGSCGAHVQAGLSDGAAAVQGPCEGAHRAGERRCADHSVGQAGTPHRHTAPQAPGTSGHGQHGLPQGTPLPRCQGPGAMGSTDFPQAHHSPGARDQGPWAARTSPRHTAPQVPGTRGHGQHGLPPRTPLPRCQGPGAMGSTDFPQAHSSPGARDQGPWAARTSPRHTAPQAPGTRGHGQHGLPPGTQLPRRQGPGAMGSTDSPQAHSSPGTRDQGPRAARTPPRHTAPQGPGTRGHGQHGLPPGTQLPRRQGPGAMGSTDSPQAHSSPGTRDQGPRAARTPPRHTAPQVPGTRGHGQHGLPPGTQLPRRQGPGATGSTDSPQAHSSPGTRDQGPWAARTPPRHTAPQAPGTRGHGQHGLPPGTQLPRDQGPGATGSTDSPQAHSSPGTRDQGPWAARTAPRHTAPQAPGTRGHGQHGLPPGTQLPRDQGPGATGSADSPQAHSSPGARDQGPWAARTPPRHTAPQGPGTRGHGQRGLPPGTQLPRRQGPGTDCMSIAGSSPQVARTPRVLGPRLRSRARHPPQNPCASN | **none** |
| **ORF128** [743638 - 741896] (REVERSE SENSE) CAWRAETPAAGRGTAPDAAAGALVSRAGPPFPGSPFPLLFPLKDKEPLSAPRQTHTARPRPTPPAPPGASTQEAQSWEACLLLLPDTVPLPHPMAARRLLGRYPCRHSGAHVPFKSRHPKGPDSPGHEDGRRRENKVRASPRSSRQEQEERGSRRLPRAQARRARGSRPLSVPISWPDSPIQPTTDLPHQQVTLRPGPHQAWAVGRTQGRAPPQPGRPGAGHRHAGGPPKLCPSAALPPPAQGHRQEDALLAEAGSYLPRRCPPPVRPYSRLTGLYNRQTAPGSTVSTGWRPAPCPGPGPRAVPPGGRAERGPQASRWGVKTTRLKIIWASTSPHSGCPSTQCQASLGRAQTSLAVEGPCPEATPAPTPLGLPRTGCPDETGAGHRPAAWGFLPCARNPSRVLITPKMGACSGAPHRPSHAQHRPPVGHSSRSAPCRLSEPKVPRWDHSQPGCSLSAAPLWCLQEVLAPLGPPRRQQLRLRLGAAMLGLFWAGPIWASKAWGVWRLSLTSSRSIEQGRADQDSPSPLIPGQHISLAPTSGDPRANCTLNSDITRAQRPTHQPQTRGWLLFTPALCQAPSEH | **none** |
| **ORF129** [731933 - 730830] (REVERSE SENSE) QAGSGTGRCIWRGRSQQEQTAPIPDPHRRQLPPRAQALLGEAVGARRQLPRQSPAPALPRPCREPQDTFPLAAASLDATPPEPRGHHQPPALPTAAPIFPSKTNSSDYTENTVGASWAQAGSKTGQSCSRNDRNTRAHQSSGTATGSRHWECPKIGHQVSTALPSAMKPSGPPAFSPAPQTESHGTFRSPEAYPGRPREVQAGGADTPMLQASWGHRRGSAWEALRPCPHEPAPRAPSPAHPASAPQGGLSRRPVSAGISPEARTGLGFSKIQQDQHQRSPAHLSTRRPNPCREDPVEGPGEHLPPAAGTHLQAHAPEDTPVRTHLQARAREDTPMRTHLQAHAREDTPLRMHLQSHAHEDTPMRTHP | **none** |
| **ORF130** [716010 - 715003] (REVERSE SENSE) LNLVPLHTRSLGWGGTGASPFPGWVPAGISHRPGLATQMEPVPGSRRQTDKGCSGDTAHLPLSCLGAQESRRPPPRASTKTGSQPAMPSPLRPQGSAGVLPEPRVPVQKPGINAASPIGTVRVERGRPTVSPAGRGSPRGGHVGGLTAPSTPGHSDHGLHTQKQSGSHAWLCCQQTAPNLPCSSSQEKRPAASLPGMVGPLRHSLGVQATHPHSTGVRGSVRPWDGPAGTGGQRVRGGRRSPTKGSSQACVGPRGAAPPGWDKAGSWLSSATAQLPQGTKGRLRDEVLTHTMGKPRHGKVGGGAARLAPRSQAGRPEGRAMQPLGRHELGSGCPQP | **none** |
| **ORF131** [692651 - 691488] (REVERSE SENSE) GHLPAKGQASLTPLGGVDPPAPAAALLHTSLPNCPLCPCRPHPGSLESLRQPAARTPRLCQPSQTFQGHQPSASTAGTLSGPGLATPGAKERPRALATAPLPTLRPPLHAAQASPQAQPGALTCPPSPVGRRGLRGRGPRRDGGRRLRTHAGGRGHSGVRRGGGVHGNGGRRWGVRGHPETEGGTDPPRMERPQGAAGVHRGLRGPKGPAPRSRTSATASPAPTGSASGLRRACVLGSVAAGGGWARSGGPRRISGSMAATAPRALPGVGLRGLPKHRPELGVCRPEPLSALRPSSGAMPLSPGPPPRARGRRAGAGRPRTGRAGGREALLRRVLPRGGRPRESHCRRGPAPPGLLVLHQHLEIVHTVTYVTLESVPQKAIYINPGFH | **none** |
| **ORF132** [663462 - 662455] (REVERSE SENSE) AAEPEVARRQTPEASSRPGAVAGVGAASRRRGALTSSDTLNLRSSATTFCLKFCCCKTGGAESEPPPELQTRRWSPFLRPRPHPGPPWESSKASAPATLMDSLVAPRGFPTGLRSDSSIPGQGGPGEACADSMVLPEASAALPRLTGPFPQHPLPGKALADPRRKVLGTHVGWAGGRVWGGACSKSLSPVGASCHPGLQNLGSSHWVSGTLSHPLGDQPYKGSPRPPFTTVGPKGALEWRLKLVFCTAGAALQASKTQLHHFGSSLPVPGTVCPQRPLECCQRRRRCPGWAVPMSLVSTRHRAPRPPALASLEPCLSLGCPGARRWTLCASAGHGL | **nef [HIV1]**  **Sequence ID: BAE95952.1**  **E value: 0.073** |
| **ORF133** [608422 - 607400] (REVERSE SENSE) ARRARRGGRGGLARPWRRPRRSLGGLGLRPGRGRAAGGPGARRAAARSHGCRAAEGTGRAPASDRPAGGDELRPLRPAPPGAPPAPRAPPAAPRAPGALRPAPAPPIGWAGRQLAASGPIAAGAARAGSEVRARFLRRRLTGRPRPPVRESAHSRACAQTPPGAPRPAPREPRARAGRVVGVHRVPASFRRRPLRKAHRRPGDAHLPTRGFPGLDTFPPPPRHAPSLPAPLPSPPLVSCRPPLGWPGCAGPARPRAPPSRPPAWVPRDAGRWQGGGRAWRRSRGLEATRGARLPSLASRAPDDPGRPLCVPASLFSAAFQRCAQDNGEQMSGAKAGSWPWG | **none** |
| **ORF134** [575132 - 573930] (REVERSE SENSE) APPTTQLPLTPSVPPSTVVHELQSAAPHRLIRITQALSTHPGSGSRRLCPHTRAQGHAGCIRTPGLRVTQAVSTHPGSGSHGLHLTPGLRITQPVSTHPGSGSHRLYPHTWAQDHAGCIHTPGLRITQAVSTHPGSGSRRLYPHTRAQDHAGCIHTPGLRITRPPPAHLGSGSRSLYPHTRAQGHAGCIHTPGLRVTQAVSAHLGSGSRRLYPHTWAQGHAGCIHTPGLRVTQALSTHLGSGSRRLCPHTWAQGHAGCIRTPGLRVTWAVSTHPGSGSCSLHLHTWAQDYAASTCTPRLRVTWAISAHPGSGSRGLYPHTRTQDHAAFTAQPGSGSCSLHLHTQAQDHTASTCTRGLRIMQPPPAHAGSGSFRLYLHTWAQDHAASTTHPGSRWAVRVLWV | **none** |
| **ORF135** [575104 - 573926] (REVERSE SENSE) PRQCPPPLWCTSCSQPPHIDSSGSRRLCPHTRAQDHAGSVHTPGLRVTQAVSAHLGSGSRRLYPHTRAQDHTASTSHLGSGSRSLYPHTRAQGHTGCIRTPGLRITQAVSTHPGSGSHRLYPHTRAQGHAGCIHTPGLRITQAVSTHPGSGSHGLHLHTWAQDHAACIHTPGLRVTQAVSTHLGSGSRRLYPHTWAQGHAGCIHTPGLRVTQAVSTHPGSGSRRLCPHTWAQGHAGSVHTPGLRVTQAVSAHLGSGSRGLYPHTRAQGHAASTCTPGLKIMQPPPAHPGSGSRGLYPHTRAQGHAGCIRTPGLRITQPSPHSRAQDHAASTCTRRLRITQPPPAHAGSGSCSLHLRTRAQDHSGCICTPGLRITQLPPHTQAHAGRCVFYGFR | **none** |
| **ORF136** [575139 - 573856] (REVERSE SENSE) TLSTSHHTASPDPVSAPLHCGARAAVSRPTSTHQDHAGSVHTPGLRITQALSTHPGSGSRRLYPHTWAQGHAGCIHTPGLRITRPPPHTWAQDHAACIHTPGLRVTQAVSAHLGSGSRRLYPHTRAQDHTGCIHTPGLRVTQAVSTHPGSGSRRLYPHTRAQDHTASTCTPGLRITQPVSTHPGSGSRRLYPHTWAQGHAGCIRTPGLRVTQAVSTHLGSGSRRLYPHTRAQGHAGSVHTPGLRVTQALSTHLGSGSRRLYPHTWAQGHVGCIHTPGLRVMQPPPAHLGSRLCSLHLHTQAQGHVGYIRTPGLRVTRAVSAHPDSGSRSLHRTAGLRIMQPPPAHAGSGSHSLHLHTRAQDHAASTCARGLRIIQAVSAHLGSGSRSFHHTPRLTLGGACSMGLDECVVTGNHCYSFIQSAFRVLKIP | **none** |
| **ORF137** [480957 - 479557] (REVERSE SENSE) NKNNTPTKKRKTKNNLSSPKLPIIGEKKRLFQMPRKNSSSQALPAKHLTKAGPSAWEEAFVYSASSAAHSWVAGAPTCSAPLLSEAALFLLSAEAWLHTHSHTHPPHIPPHTHTHAHIPPHTHPLYTPPHIHIPPHTYPPPPHKHTPHTYPHTHTHTHTHTSHTPPIHTHTHPHTHPHTHTPYTHTPTLSHTHPYTYTPHTYPPHSHTHTPIHIRPTHIPPTLTHTHPPHPPYPPQRYPHTHSPHTHLPHTPYTHPHIPPHPLTYPTHTPHTHTPPHTHTPTFTPTHPPTYSPTLTHIPPHTHTPTPTHIPHTHTPYTHTPPHTYPHIHSHTPPHILPHTHTHTPPHTYPHTHSHTPHTHPIHTHPPTHIPPHSLPHTPPHTPPHSHTYPPHTHVHPPHTAPHTHLHTHTYCFSPLHTQTHRDAQIHTHTHTHTHTAQPPAAPPGVVSAVSPQHPTASPGAHHSAHL | **none** |
| **ORF138** [480884 - 479478] (REVERSE SENSE) VKRKGFSRCPEKTAVPRLSLPSTSRRQDLQPGRKPLCTQPLLQRTPGLQVLQPAQPLCLVKPPYSYSVQKLGCTHTHTHTPHTYPPTLTHTHTYPHTHTPYTHPHIYISPHTHTPHPPTNIPPTHTPTHIPTHTHTPPTHPLYTPTHTHTLTPTHIPPTHIPPHSHTHTHTHTPHTHTPHTHTHIPPYTYAPHTYPPHSHTHTPHTPHTPHKDTPTHTAHTHTSHTPPIHTHTYPHTHSHTPHTHPIHTHPPTHIPPHSLPHTPPHTPPHSHTYPPTHIPPHPLTYPTHTPHTHTPPHTHTPTFTPTHPPTYSPTLTHIPPHTHTPTPTHIPHTHTPYTHTPPHTYPHIHSHTPPHILPHTHTHTPHTLMYTPHTQPPTHTSTHTHTASLHYTHRHTEMHRYTHTHTHTHTRPSPLLLLRVWSLLYPPSTPLPVLEPTTLLTYEHSALNSCPHPVDFCAPHPRPPDPPS | **none** |
| **ORF139** [480703 - 479375] (REVERSE SENSE) SRPIPTQCRSLAAHTLTHTPPTHTPPHSHTRTHTPTHTPPIHTPTYTYPPTHIPPTPPQTYPPHIPPHTYPHTHTHLPHTPYTHPHTPTHSPPHTYPLHTYPHTLTHTPIHIHPTHIPPTLTHTYPHTHTPHTHTPHTHTHTPPTPPIPPTKIPPHTQPTHTPPTHPLYTPTHTPTPTHIPHTHTPYTHTPPHTYPHIHSHTPPHILPHTHTHTPPHTYPHTHSHTPHTHPIHTHPPTHIPPHSLPHTPPHTPPHSHTYPPTHIPPHPLTYPTHTPHTHTPPHTHTPTFTPTHPPTYSPTLTHIPPTHSCTPPTHSPPHTPPHTHILLLSITHTDTQRCTDTHTHTHTHTHGPAPCCSSGCGLCCIPPAPHCQSWSPPLCSLMSTLHSTPAHTQWTSVPHTPDLLTPHPDPPPASPQLSLSRDTPSALTSESPPLAFLHPSRP | **none** |
| **ORF140** [454054 - 452849] (REVERSE SENSE) ATVTRWPGLIGTTAPAPCCFPRAPLAIGCRPWWQRHACCTLDVNEGRKERRKEPNARRLGSGWVLQVLPGVGKGLARCSCEGGAPGRWVDRLGWVGGGGQEPWEPQGCSELRGRGQSPGGGVDGALGGVDGADGGGLWTEPLGGVDTVLGVVWTESWGVWMEPWGGMDGACWGVWMELWGGMDGALGGCGWSMLGGVDRALGGVWTEPCWGVWMEHVGVCGWSMLGGVDGTRGVWMELWGVWMEPWGVWMEHVGGCGQSSGGGCGRSMLGCVDGTLGGCGWSPGGVWMELWGGVDGACCGVWMEPWGGDGACWGVWMEPRWGMWIEFFGGVDGALGGVNRARRGVWTEPWEACGWNSGDCLAPVGRGLRALVTSGSPGDGRGLGPVAWRGHGGCGKTGLRGA | **none** |
| **ORF141** [442572 - 441379] (REVERSE SENSE) LLTVLPAIVIDGCGSDAGPGMPGTAASGVCGPHGRCVSQPGGNFSCICDSGFTGTYCHESEWPRTAGWWWGWAGLRPWLTPLASADIDDCLGQPCRNGGTCIDEVDAFRCFCPSGWEGELCDTSECSSTRPHGLCLHPCGPLITLRWTAVWVRQAPYPERPGQGVLPPWGGVPGCPHARGQLPRPDAPPPPLSVLTWPSSSASSPGKPSLSAGDPPPADRRPRPQIPTTAFPIPATAAAAATTWSMTSTVRATTAGRARPATHVSVRRPWPPGAAPRTLALAVWGLPAERPMCQQASSSAMPTPAATVAPATTAATPSAAPAPPAGRAAPAPSVRSPRCLCDRRAYALPGTAPSGAMGRGVFFEGHTCHLPPAPCPRVCLPCLGWGRGMETQGQPRAR | **none** |
| **ORF142** [437342 - 435765] (REVERSE SENSE) GPHTSGPWALLCARMGTASTPPLLPPLLPTHCLVVKSPLQDPGLSSPVRPPCSSVARASVLGAGCWEPGALEPSPQCRSLPQSFSPARDLPDSSLIQGAAHAIVAAITQRGNSSLLLAVTEVKVETVVTGGSSTGKRGRWAPGKAPGRQLRHWAQSRPILPILPATRNTEVPGTCCPSRPAPQPDLPSPRPGTLPTSQQPEGGARPPGPQGPPRPGCVASAPQVPRPHCPPALLPGPQVCWCLCCVVPSACCGWRAWSCACGGHASAGKSGRGAGCRGRRAPTTSGPRSTPSATPLSGRGATRTCSTSARTSRRRRAGRTRRCPGRPATRPSGRMRRTRIWAAVRRTPWRRRSSSHTNSPKILAARRGGRPTGPQAPKWTTARSGASMRPATPARSRGGCQLGRDPGPSVGAMPSAGPGGRGHVHSFFILCKKTTKNKNQMFIFYVSLTLYKLFSNCQAENNGVFSDSCYFCKVSVRGTRCMKGESKGCLRRHQIVAFVTRGCALFTESSFYSSLGFLCGSRPKCR | **none** |
| **ORF143** [345618 - 344455] (REVERSE SENSE) SLSQVVSRDSRPHSCLCPTGNATPVTTTAPWASLGLSAKTCNNVSFEESRIVLVVVYSAVCTLGVPANCLTAWLALLQVLQGNVLAVYLLCLALCELLYTGTLPLWVIYIRNQHRWTLGLLACKVTAYIFFCNIYVSILFLCCISCDRFVAVVYALESRGRRRRRTAILISACIFILVGIVHYPVFQTEDKETCFDMLQMDSRIAGYYYARFTVGFAIPLSIIAFTNHRIFRSIKQSMGLSAAQKAKVKHSAIAVVVIFLVCFAPYHLVLLVKAAAFSYYRGDRNAMCGLEERLYTASVVFLCLSTVNGVADPIIYVLATDHSRQEVSRIHKGWKEWSMKTDVTRLTHSRDTEELQSPVALADHYTFSRPVHPPGSPCPAKRLIEESC | **none** |
| **ORF144** [315084 - 313939] (REVERSE SENSE) EAFGDNPGLWRGLDRPRSPDRPGIAVPGSSRPRHGCLGGEVEPGRSERVQTSVCPATPARGPHVGPRRRRVQFRARRAETWELAGRGRTGRGETWEPAGRGRGGAGRRGGGGATEGAREPQRPSPQRGKTRHRCPARVGLNKRPRPRPASGQSAGVARGERSVDPLWTPSGGRSPRPAPSGAPSSAAGSWRQRRWRWLSRGPGGRPRGSGERARRTARASTAACTFVLQGAEEPGGGARSLWLLALGPGRRRRPGPPLGLPRRPRRPPGRWFPRGVAAAEGARGTEGPGPPSAFLAERGAGALPRPAGGPTREPLHEGPAGVRSGVSRTSALVAAEGTPGAPQPPLSEPGRGEGRGCGRRPRPRAGRPAPSSLGSSGGARWD | **none** |
| **ORF145** [273595 - 272423] (REVERSE SENSE) VCVSAWPFRYSLASSHPRKRCPCVCVCVCVYDRACVFVSLCVCVCVSLCVCLCVCECASMCTCVSLCLCLCPCVSVCLCVCLCVPLCVCLYVYLCICVSVSLSVCVCVSLCLYLCMYVCVCVSVCVSVCVCVPASDPHPWTLPGSSSWRWRSSPGSLPALPSPTIPAELHSFWGMPGLGLHQARRHQTLRTPLGPCPPCPHPQVCCLCVVPKLDPREAPTLCTLILTKSPLPAYPFTPSHTMAVVPLLLFPAVPHANTPSHLRSLSSALTTLRDPAWSLKWHLLPTGAPSASLLPCPRPLGLTFNPSPFAPILCTHATLSPLSRTLPTLPGTCLTPKVPAYTARSPPGPPFPRPQGSAALQNASSLQACLCLRQTWSPWGRASGVLVAAVS | **none** |
| **ORF146** [271210 - 270086] (REVERSE SENSE) KPGPPGPGAPAPGLPSHLLCGRAARLVQESGLSTLVRGFQAGLGCVYCVAVWWGRWLGGGCGRARPEVWGLVRGLNGVKVRPQGPAVLGKGKWVLPTGMPPGMVSSLPLSRRLRHVYLLLFVCLSLHWAWFRGLGVSASWRAGLRGAGQSLGRWVYAAAADHTQGIGSHWPGGWAGRHWCRLDPTPWGKQVNANGADNAAGERGAPGLGMPPSCSRHYQGPSCGLSVWCALRVGGLSGGLQSREAPTAPASRCRRWEPKVFGHSSGRMMAPVGWRPLAPSSFTMTGPAHMAAGRPPQREKGRAPRHGTTTLPPASLHSLSALLPPHSRAPRDSQAAGSRTISSTPCLHRGLCWLHGSQPVWGSPSLCPAYGTIRS | **none** |
| **ORF147** [248410 - 231524] (REVERSE SENSE) DHYARGAGPQLASLPCWLSLGGDSTPTPSVPGPGPQASWTGTNRFPLPMFQDTDVADGCRETPTKTLEGDGDQERLISKPRVGRGRQSQRERLSWPKFQSIKSKRGPGPQRSHSSSEAYEPRDAHDVSPTSTDTEAQLTVERQEQKAGPGSQRRRKFLNLRFRTGSGQGPSSTGQPGRGFQSGVGRAGVLEELGPWGDSLEETGAATGSRREERAEQDREVMPAQSMPLPTELGDPRLCEGTPQEGGLRAARLHGKTLEGQAQETAVAQRKPRAQPTPGMSREGEGEGLQSLEIGIARLSLRDTTEGGTQIGPPEIRVRVHDLKTPKFAFSTEKEPERERRLSTPQRGKRQDASSKAGTGLKGEEVEGAGWMPGREPTTHAEAQGDEGDGEEGLQRTRITEEQDKGREDTEGQIRMPKFKIPSLGWSPSKHTKTGREKATEDTEQGREGEATATADRREQRRTEEGLKDKEDSDSMTNTTKIQLIHDEKRLKKEQILTEKEVATKDSKFKMPKFKMPLFGASAPGKSMEASVDVSAPKVEADVSLLSMQGDLKTTDLSVQTPSADLEVQDGQVDVKLPEGPLPEGASLKGHLPKVQRPSLKMPKVDLKGPKLDLKGPKAEVTAPDVKMSLSSMEVDVQAPRAKLDGARLEGDLSLADKEVTAKDSKFKMPKFKMPSFGVSAPGKSMEDSVDVSAPKVEADVSLSSMQGDLKATDLSIQPPSADLEVQAGQVDVKLPEGPVPEGAGPKVHLPKVEMPSFKMPKVDLKGPQIDVKGPKLDLKGPKAEVTAPDGEVSLPSMEVDVQAQKAKLDGAWLEGDLSLADKDVTAKDSKFKMPKFKMPSFGVSAPGKSIKALVDVSAPKVEADLSLPSMQGDLKTTDLSIQPASTDLKVQADQVDVKLPEGHLPEGAGLKGHLPKVEMPSFKMPKVALKGPQVDVKGPKLDLKSPKAEVTAPDVEVSLPSVEVDVEAPGAKLDSARLEGELSLADKDVTAKDSRFKMPKFKMPSFGASAPGKSIEASVDVSAPKVEADVSLPSMQGDLKTTDLSIQPPSADLEVHAGQVDVKLLEGHVPEGAGFKGHLPKVQMPSLKMPKVDLKGPQVEVRGPKLDLKGHKAEVTAHEVAVSLPSVEVDMQAPGAKLDGAQLDGDLSLADKDVTAKDSKFKMPKFKMPSFGVSAPGKSIEASVDLSAPKVEADMSLPSMQGDLKTTDLSIQPPSTDLELQAGQLDVKLPEGPVPEGAGLKGHLPKLQMPSFKVPKVDLKGPEIDIKGPKLDLKDPKVEVTAPDVEVSLPSVEVDVEAPGAKLDGGRLEEDMSLADKDLTTKDSKFKMPKFKMPSFGVSAPGKSIEASVDVSAPKVEADVSLPSMQGDLKATDLSIQPPSADLEVQAGQVDVKLPEGPVSEGAGLKGHLPKVQMPSFKMPKVDLKGPQIDVKGPKLDLKGPKVEVTAPDVKMSLSSMEVDVQAPRAKLDGAQLEGDLSLADKAVTAKDSKFKMPKFKMPSFGVSAPGKSIEASVDVSEPKVEADVSLPSMQGDLKTTDLSIQSPSADLEVQAGQVNVKLPEGPLPEGAGFKGHLPKVQMPSLKMPKVALKGPQMDVKGPKLDLKGPKAEVMAPDVEVSLPSVEVDVEAPGAKLDSVRLEGDLSLADKDVTAKDSKFKMPKFKMPSFGVSAPGKSIEASVDVSAPKVEAEVSLPSMQGDLKTTDLCIPLPSADLVVQAGQVDMKLPEGQVPEGAGLKGHLPKVDMPSFKMPKVDLKGPQTDVKGAKLDLKGPKAEVTAPDVEVSLPSMEVDVQAQKAKLDGARLEGDLSLADKDMTAKDSKFKMPKFKMPSFGVSAPGRSIEASVDVPAPKVEADVSLPSMQGDLKTTDLSIQPPSADLKVQTGQVDVKLPEGHVPEGAGLKGHLPKVEMPSLKMPKVDLKGPQVDIKGPKLDLKDPKVEMRVPDVEVSLPSMEVDVQAPRAKLDSAHLQGDLTLANKDLTTKDSKFKMPKFKMPSFGVSAPGKSIEASVDVSPPKVEADMSLPSMQGDLKTTDLSIQPLSADVKVQAGQVDVKLLEGPVPEEVGLKGHLPKLQMPSFKVPKVDLKGPEIDIKGPKLDLKDPKVEVTAPDVEVSLPSVEVDVKAPGAKLDGARLEGDMSLADKDVTAKDSKFKMPKFKMLSFGVSALGKSIEASADVSALKVEADVSLPSMQGDLKTTDLSVQPPSADLEVQAGQVDVKLPEGPVPEGAGLKGHLPKLQMPSFKMPKVDLKGPQIDVKGPKLDLKGPKTDVMAPDVEVSQPSVEVDVEAPGAKLDGAWLEGDLSVADKDVTTKDSRFKIPKFKMPSFGVSAPGKSIEASVDVSAPKVEADGSLSSMQGDLKATDLSIQPPSADLEVQAGQVDVKLPEGPVPEGAGLKGHLPKVQMPSFKMPEMDLKGPQLDVKGPKLDLKGPKAEVTAPDVEMSLSSMEVDVQAPRAKLDGARLEGDLSLADKGVTAKDSKFKMPKFKMPSFRVSAPGESIEALVDVSELKVEADMSLPSMQGDLKTTDISIQPPSAQLEVQAGQVDVKLPEGHVPEGAGLKGHLPKLQMPSFKMPEVDLKGPQIDVKGPNVDLKGPKAEVTAPDVKMSLSSMEVDVQAPRAKLDGARLEGDLSLADKGMTAKDSKFKMPKFKMPSFGVSAPGKSIEASVDVSELKVEADGSFPSMQGDLKTTDIRIQPPSAQLEVQAGQVDVKLPEGHVPEGAGLKGHLPKVQMPSFKMPKVDLKGPQIDVKGPKLDLKGPKAEVTAPDVEVSLPSVEVDVEAPRAKLDGARLEGDLSLADKDVTAKDSKFKMPKFKMPSFGVSAPGKSIEVSVDVSAPKVEAEVSLPSMQGDLKTTDISIEPPSAQLEVQAGQVDLKLPEGHVPEGAGLKGHLPKLQMPSFKMPKVDRKGPQIDVKGPKLDLKGPKTDVTAPDVEVSQPGMEVDVEAPGAKLDGARLEGDLSLADKDVTAKDSKFKMPKFKMPSFGVSAPGKSIEVLVDVSAPKVEADLSLPSMQGDLKNTDISIEPPSAQLEVQAGQVDVKLPEGHVLEGAGLKGHLPKLQMPSFKMPKVDRKGPQIDIKGPKLDLKGPKMDVTAPDVEVSQPSMEVDVEAPGAKLDGARLEGDLSLADKDVTAKDSKFKMPKFKMPSYRASAPGKSIQASVDVSAPKAEADVSLPSMQGDLKTTDLSIQLPSVDLEVQAGQVDVKLPEGHVPEGAGLKGHLPKVEMPSFKMPKVDLKSPQVDIKGPKLDLKVPKAEVTVPDVEVSLPSVEVDVQAPRAKLDGARLEGDLSLAEKDVTAKDSKFKMPKFKMPSFGVSAPGRSIEASLDVSAPKVEADVSLSSMQGDLKATDLSIQPPSADLEVQAVQVDVELLEGPVPEGAGLKGHLPKVEMPSLKTPKVDLKGPQIDVKGPKLDLKGPKAEVRVPDVEVSLPSVEVDVQAPKAKLDAGRLEGDLSLADKDVTAKDSKFKMPKFKMPSFRVSAPGKSMEASVDVSAPKVEADVSLPSMQGDLKTTDLSIQPPSADLKVQAGQMDVKLPEGQVPEGAGLKEHLPKVEMPSLKMPKVDLKGPQVDIKGPKLDLKVSKAEVTAPDVEVSLPSVEVDVQAPRAKLDSAQLEGDLSLADKDVTAKDSKFKMPKFKMPSFGVSAPGKSIEASVHVSAPKVEADVSLPSMQGDLKTTDLSIQPHSADLTVQARQVDMKLLEGHVPEEAGLKGHLPKVQMPSFKMPKVDLKGPEIDIKGPKLDLKDPKVEVTAPDVEVSLPSVEVDVEAPGAKLDGARLEGDLSLADKDMTAKDSKFKMPKFKMPSFGVSAPGKSMEASVDVTAPKVEADVSLPSMQGDLKATDLSVQPPSADLEVQAGQVDVKLPEGPVPEGASLKGHLPKVQMPSFKMPKVDLKGPQIDVKGPKLDLKGPKAEVTAPDVKMSLSSMEVDVQAPRAKLDGVQLEGDLSLADKDVTAKDSKFKMPKFKMPSFGVSAPGKSMEASVDVSELKAKADVSLPSMQGDLKTTDLSIQSPSADLEVQAGQVDVKLPEGPLPKGAGLKGHLPKVQMPCLKMPKVALKGPQVDVKGPKLDLKGPKADVMTPVVEVSLPSMEVDVEAPGAKLDSVRLEGDLSLADKDMTAKDSKFKMPKFKMPSFGVSAPGKSIEASLDVSALKVEADVSLPSMQGDLKTTHLSIQPPSADLEVQAGQEDVKLPEGPVHEGAGLKGHLPKLQMPSFKVPKVDLKGPQIDVNVPKLDLKGPKVEVTSPNLDVSLPSMEVDIQAPGAKLDSTRLEGDLSLADKDVTAKDSKFKMPKFKMPSFGMLSPGKSIEVSVDVSAPKMEADMSIPSMQGDLKTTDLRIQAPSADLEVQAGQVDLKLPEGHMPEVAGLKGHLPKVEMPSFKMPKVDLKGPQVDVKGPKLDLKGPKAEVMAPDVEVSLPSVETDVQAPGSMLDGARLEGDLSLAHEDVAGKDSKFQGPKLSTSGFEWSSKKVSMSSSEIEGNVTFHEKTSTFPIVESVVHEGDLHDPSRDGNLGLAVGEVGMDSKFKKLHFKVPKVSFSSTKTPKDSLVPGAKSSIGLSTIPLSSSECSSFELQQVSACSEPSMQMPKVGFAGFPSSRLDLTGPHFESSILSPCEDVTLTKYQVTVPRAALAPELALEIPSGSQADIPLPKTECSTDLQPPEGVPTSQAESHSGPLNSMIPVSLGQVSFPKFYKPKFVFSVPQMAVPEGDLHAAVGAPVMSPLSPGERVQCPLPSTQLPSPGTCVSQGPEELVASLQTSVVAPGEAPSEDADHEGKGSPLKMPKIKLPSFRWSPKKETGPKVDPECSVEDSKLSLVLDKDEVAPQSAIHMDLPPERDGEKGRSTKPGFAMPKLALPKMKASKSGVSLPQRDVDPSLSSATAGGSFQDTEKASSDGGRGGLGATASATGSEGVNLHRPQVHIPSLGFAKPDLRSSKAKVEVSQPEADLPLPKHDLSTEGDSRGCGLGDVPVSQPCGEGIAPTPEDPLQPSCRKPDAEVLTVESPEEEAMTKYSQESWFKMPKFRMPSLRRSFRDRGGAGKLEVAQTQAPAATGGEAAAKVKEFLVSGSNVEAAMSLQLPEADAEVTASESKSSTDILRCDLDSTGLKLHLSTAGMTGDELSTSEVRIHPSKGPLPFQMPGMRLPETQVLPGEIDETPLSKPGHDLASMEDKTEKWSSQPEGPLKLKASSTDMPSQISVVNVDQLWEDSVLTVKFPKLMVPRFSFPAPSSEDDVFIPTVREVQCPEANIDTALCKESPGLWGASILKAGAGVPGEQPVDLNLPLEAPPISKVRVHIQGAQVESQEVTIHSIVTPEFVDLSVPRTFSTQIVRESEIPTSEIQTPSYGFSLLKVKIPEPHTQARVYTTMTQHSRTQEGTEEAPIQATPGVDSISGDLQPDTGEPFEMISSSVNVLGQQTLTFEVPSGHQLADSCSDEEPAEILEFPPDDSQEATTPLADEGRAPKDKPESKKSGLLWFWLPNIGFSSSVDETGVDSKNDVQRSAPIQTQPEARPEAELPKKQEKAGWFRFPKLGFSSSPTKKSKSTEDGAELEEQKLQEETITFFDARESFSPEEKEEGELIGPVGTGLDSRVMVTSAARTELILPEQDRKADDESKGSGLGPNEG | **none** |
| **ORF148** [227938 - 226814] (REVERSE SENSE) DKPGCFENPSDPAFIAEEWELSCQTWVQILHLQDRCPGGSQDRVSSSGAASKSWSQTGVTFLPSSSNAGPLSPIHQPWALHPQDKPAPTCLDPPCTTAHKPHSLVHPYPRPPVSPARTQDLSPQPRVPSRPPRPLSPGSPPALPVPSAAGPLPPSPSPQPRVPSHPPLTQPRIASHPPRPLSRRSPPALPVPSAPLPSRPPLTQPRVASRPPGPLSRGSPPALPVPSAAGPLPPSPHSAAGRLPPSRSPQPRVPSRPSLTQPRVASRPPCPLSRRSPPALPVPSAPLPSRAAPVRRRRSCPLAVGARRCALVCVSSAGVPGVGSAPLASPGSLGAGIWVPERGSPARGGRWRVRSAWEARLVPAPPRPRFSRTRG | **gag protein [SIV]**  **Sequence ID: NP_056802.1**  **E value: 3.8** |
| **ORF149** [200761 - 199343] (REVERSE SENSE) GFNFSLWNFPSHPQSGDRQVSLPVHWSHKSSLSGPFPCPLNTHLGAGFSMSSLPSPSIRPHPLTGWLEHLGCLASRAISGPINVPEILQCQLPDHPGSWFPPAATAPCASWLSVPTCSVSSLPVLARGSHLQRQLPARPGSWFPPAATAPCASWLSVPTCRVSSLPCPSRLAVPTCSDSSLGFLALGSHLQCQLPDHPGSRFPPAASAPCPSWLAVPTCSDSSLPVLAHGSHLQRQLPARPGSRFPPAGSAPCPARPGSRFPPAATAPWASWLSVPTCSDSSLRVLALGSHLQRQLPARPGSRFPPAATAPCPSWLMVPTCSDSSLRVLALSSHLQRQLPARPGSRFPPAASAPCPSWLMVPTCSDSSLRVLALGSHLQRQLPARPGSRFPPAASAPCPSWLMVPTCSDSSLRVLALGSHLQRQLPARPGSRFPPAASAPCPSWLMVPTCSDSSLRVLALGSHLQRQLPARPG | **none** |
| **ORF150** [187369 - 186014] (REVERSE SENSE) VSAHAAVLVSGRPPQPGSRLTSSSRQGALCSHGWPATAGTPGSASGYRRSPARARPQHGPALEEPRPCSPHTQLRGGSGHRASSRFRGRVLLARASSQLSFMFWSKSREFSEPGGTFWKLRLASGMRCTSCKREGRHRGPQAEAPARLWLHLPAPARQVDRAHPPSVCARSLLCPCPAMEAAGLGMTAQSGLTQVPRAPQTSSSPVAVWGADPGGHVPARQVGMCSRASAEACKGLWGPGLTLLEGGSGTGASESLGLTSSRAEMVEAGVLGMLLREALWAGSEEPSVSPSPATSWISWARMATSFTSVWLSLQPAGKTAGSAQRKSGQQGPDRPQSPSQLPGSCWPEVGPGGGAPQPGPKHRPSHWLHRHWPRPQREAKLPTATRRDGGGLGRSLKRRVHRNPCWLWGRPHLLSLRPPRASITAAGPKWDGWDSVIERVGATGAQLCRQRN | **none** |
| **ORF151** [184648 - 183233] (REVERSE SENSE) GSSFLMKTFEEALLVLAVSPPGKICIHPPLHPPTHSPSIHPSIHPPTHPSIHPSIHPPTHPSIHTHPPIHPHTHPPIHPPTHPSIHPLIHPSIHPSLHPPTHSSIHPSTHPPTHSSIHPSIHPSTHPLIHPSIHPSTHSSIHPPTHSSIHPSIHPPTTHPSIHPPTHSPIHPSTHSLIHSSIHPSTHPLIHPSIHPPTHSPIHPSIHPPTHPPTHPSIHSSIHLPTHSSIHPPTHSFIHPSIHPPTHSPIHPLTHPFIHPSIHPSIHSSIHPPTHSSIHPSTHSSIHPSTHPPTHPSIHPPTHSSIHPSIHPSTHPPSYSSIHPSIHPPTHLPTHPSIHPSIHASIHPSTHPPSHPSIHPPTHPPTHPAIHPSTHPATYPSTCPPAHPSIHPPTHPPSYPSIHPPTLLSIHPFIHPSIHPSIHPSIHPFVHPSSQPAICRTIYPLKKGAPSLVPALRVLSLKKTHWHPVDLL | **none** |
| **ORF152** [184619 - 183183] (REVERSE SENSE) GGPPCTGGLTSWEDLHPPTPPSTHSLSIHPSIHPPTHSSIHPSIHPPTHSSIHPHPPTHPSTHPPTHPSTHPPIHPPTHPSIHPSIPPSTHPLIHSSIHPPTHSFIHSSIHPSIHPPTHPSIHPSIYPLIHPSTHSLIHSSIHPSTHHSSIHPSTHPLTHPSIHPLTHPFIHPSIHPPTHPSFHPSTHSLTHPSIHPSTHPPTYSPIHPFIHPSTHPLIHPSTNPLIHPSIHPSTHPLTHPPTHSSIHPSIHPSIHPLIHPSTHPLIHPSIHTLIHPSIHPPTHSPIHPSTHSLIHSSIHPSIHPPTLLFIHPSIHPPTHPPTHPSIHPSIHPCIHPSIHPPTLPSIHPSTHPPTYPPSHPPIHPPSNLSIYLPTCPSIHPSTHPPTLLSIHPPTHPPIHPSIHPSIHPSIHPSIHPSICPSIQPASHLPYHLSTQEGSSQPGPCPQGALTQENALAPSGFTLNGLLTWQSPQSLPSAR | **none** |
| **ORF153** [184629 - 183175] (REVERSE SENSE) KPLRRPSLYWRSHLLGRSASTHPSIHPLTLHPSIHPSTHPLIHPSIHPSTHPLIHPSTPTHPSIHTPTHPSIHPPTHPSTHSSIHPSIHPSIHPPTHPFIHPPTHPLIHPFIHPSIHPPTHSSIHPSIHLPTHPSIHPLTHPFIHPSIHPPLIHPSIHPPTHPSIHPPTHSSIHPSIHPPTHSSILPSIHPLTHPSIHPSIHPPTHLLTHPSIHPSIYPPTHPSIHQPTHSSIHPSIHPPTHPSTHSLIHSSIHPSIHPSTHPSIHPPTHPSIHPHTHPSIHPPTHPLTHPSIHPLTHPFIHPSIHPPTHPPIHPSIHPSTHPPTYPPIHPSIHPSMHPSIHPPTHPPIHPSIHPPTHLPTQPSTHPPTQQPIHLPAHLPIHPSIHPPTHPPIHPSTHPPSYPSIHSSIHPSIHPSIHPSIHLSIHPASQPSAVPSIHSRRELPAWSLPSGCSHSRKRTGTQWIYSEWATHLAISAISAVCTMKP | **none** |
| **ORF154** [178943 - 177432] (REVERSE SENSE) RHPLGHAPVIRLISVPSDDWAKGGRRCPGEFRGLQHPKDLPRGNGPRHRGSEKPTSQPSTDTGHPQATGRAGTWLARVAGGRDMQPGCQQDMRMGYGPSPGKGRGGAVGSIPGSLLPSWARGRSQGWAGAGQRPPTSTPGIASTPRSPRVLSLATPGIASTPDSPRGLSLATPGIASTPRSPRVLSLATPGITSTPRSPRGLSLATPGITSTPPFPTRALTRHPRHHLHPPVPHEGSHSPPPASPPPPVPHEGSHSPPPASPPPPVPHEGSHSPPPASPPPPVPHEGSHSPPPASPPPPRSPRGLSLAAGLHLVDHLPGLLHLRVLRLGLDGVCPCVTEAVVLLLFPCLGQLGVRGGGRWGRGAHGAGPMGCQGSGQHRRNLLGAGPGGRGALPQTLGHEVLGQAGAAPHASEGAGRGSGGGRGAAQPFLCLLPLERAGPLWPRAAAPCLDWGWDTGAVPIGRRWATALVGLGVPGRAVREGLGALLSGCVLVEELDDKGLLVM | **none** |
| **ORF155** [179071 - 177200] (REVERSE SENSE) PQVRLGCCPLPAQPLLGVAVCACTWCSAWGAAPSGREANSCSSAIRWATPPSSDSSLSPLMTGRKVAEDALESSGDSNTPKTCQEEMVPGTEGQRSPPPNPPLTLATPRPQGEQGPGWPEWLVAGTCSLAVSRTCGWDTDPALGRGGAGLWAAYQAPSCPAGPEAGARAGPEQDNVRPPPPPASPPPPVPHEYSHSPPPASPPPPIPHEGSHSPPPASPPPPVPHECSHSPPPASPPPPVPHEGSHSPPPASPPPPRSPRGLSLATPGITSTPPFPTRALTRHPRHHLHPRFPTRALTRHPRHHLHPPFPTRALTRHPRHRLHPPFPTRALTRHPRHRLHPPVPHEGSHSPLGCTWSIIFRASSTSVSCASVSMVYVPASLRLSSSSCFRALVSWGSVGAGGGVGGPTGLVPWGVRGAGSTGGTFSGLGRAAGELSRRHWAMKSWARRERRPTLPKGRAGVREAGEGLPSRSSVFSRLSEPALCGPELRPPAWTGAGTLGRSLSAGVGPRRLSALGSPDGLCVKDWERFFRGVSSSKNSMTKACWLCSSSWGSRLICLCSRTCSPLASKGTPAAEAALARGSSESLCVPSSCLWPGGDRGPHLVRPRERKQLSISGSLITPCLS | **none** |
| **ORF156** [166241 - 165240] (REVERSE SENSE) QQPGLWAHPPASSPEFPIGGHRHPQQTGSMHGAQKVRACPGCGLWGSTQECKSQLSHALTEDTSPQTCSTHMHTTHTDVCTYRHAHVCTCTLTHAHTHGTHAHTRHTHTHTHGTHTHTRTAHPRHTHAHPRHTCTPMAHTRGTHTHTHAHPRTPTAHTHTHGTHTRHTHGTPTRTHMAHTRTHARHTHGTHTHTHGTHTAHTRTHTRHTRTHTAHTRTHTTHTRTPWTTAWATTGVTATAGSPAWVHVPASFNACSLGTSSVPGLHWAQGHCGEQRQGPGQRGPWDSTWVAALPGTQLPYPSTCCGPSYRPSCLVKAQASGRPLCLSPLHVPSP | **none** |
| **ORF157** [148724 - 147693] (REVERSE SENSE) IHPSTEPCVHPSTPRSIHLHSPSIHPSKPPSIHPSTPPSIHPSTPPYIHPSTPPSIHPSTPTSIHPSTPPSIHPSTPPSIHPSTPPSIHPPLPPPFNLQSPLHSPLHSPHSPLHSPLHPSIPPSIHPSTPPYIHPSTRPYIHPSPTPYIHPSTPSLHSYLHFPLHSFLHCPLHSSTAPSIPPSTAPSVHPSTAPYIHPSPIPSIHPSTPSLHSSFHGPLHSSLHCPLHPSTAPSIPPSTAPSVHPSTAPSVHPSPIPSIHPSTPSIHPSTAPSIYPSTAPSIPPSTAPSIPPSTAPSVHPSTAPSVHPSTAPYIHPSPIPSIHPSTPSLHSSLHCPLHSSLHCS |  |
| **ORF158** [149121 - 147640] (REVERSE SENSE) ATTVPPHSSLLYPLNSSFSGTLRSSLHSPLHSSLHSLLHSPLHSPLHSPFHSPLHSSFHYPLNSSFHRTLRSSLHSPLHSPPLSLHSPLQTPLHSPLHSPLPSPSTPCSFHPSTPPHSPFHSPLHSSFHYPLNSSFHRTLRSSLHSPLHSPPLSLHSPLQTPLHSSLHSPLHSPLHSPLHSPLHSPLHSPLHSHLHSPLHSPLHSPLHSPLHSPLHSPLHSPSTPPSLQPPVPSPFTSPLPPFTPPLPTPSLHSPLHSSLHSPLHSSLHSPLHSSLPYSLHSSFHSLTPFIPPLPPPFIPPLPPPFLHCPLHSSLHCPLRSSLHCPLHSSLPYPLHSSLHSLTPFILPWPPPFIPPLPPPSLHCPLHSSLHCPLRSSLHCPLRSSLPYPLHSSLHSLHSSLHCPLHLSLHCPLHSSLHCPLHSSLHCPLRSSLHCPLRSSLHCPLHSSLPYPLHSSLHSLTPFIPPLPPPFLPPLLLTAGSGAVLCVPWCRVGQ | **none** |
| **ORF159** [149362 - 147590] (REVERSE SENSE) GVGDTPQLSNDANPPGPGWEQLSASWLRGCVPYVPFGASLLKKTVSACTLLRKHPDTWLQRAESTVPFPDTHQHPHPLHLSHHCPAAFIPPLPPKFIFQWNSAFIPPLPAPFIPPLPAPFTPPLPPPFTLPLPPPFILPLPPKFILPQNPAFIPPLPAPFTSTLPPFTPPNPPPFTPPLSPPFTLHSLLLSPLHSPPFTLPLPPPFILPLPPKFILPQNPAFIPPLPAPFTSTLPPFTPPNPPPFIPPLPPPFTPPLPPTFTPPLPPPFTPPLPPPFTPPLPPPFTPPLPPPFTPPLPPPFTLHSPLPSTSSPLSIHLSTPPIHPSTPHSIPPFPPPFIPPLPPTFIPPLAPTFIPPLLLTFILPLPHSIHTSTSPSIHSSTAPSIPPLPPPFLPPLPPPFIPPLPPTFIPPLSPPFIPPLPHSIHPSMAPSIHPSTAPSIPPLPPPFLPPLPPPFIPPLPPPFIPPLSPPFIPPLPPFIPPLPPPFIPPLPPPFLPPLPPPFLPPLPPPFIPPLPPPFIPPLPPTFIPPLSPPFIPPLPHSIHPSTAPSIPPSTAPDCRLWGSALCPMVQSRTMMRRQKSNRVEFRRVLS | **none** |
| **ORF160** [138125 - 136980] (REVERSE SENSE) GVNERDIKSLPEDSHLQGLGLQQVWPWAGGCAGRKPASRSPSGRMRATVAAPGLLVIWEELRGPSEPGFPHLHGLRTPGRGSRARLGQARSCLGAAGLETAGAEGSFRGTGGAEKRRKEVGTDEKILKCVPGFPYKSRVAESYFEIQSPPTPGGLGVLGSVSFFSPEFRNPPPRAQSPGFQQPGRRSARRVSGIRAMRLCSPGSQPGTQNLHPGPREYRTPVGTGIIPRPGGTETLREACAALGEGRGGSRHSCPRRVGRRSVLKGGQGSFVLNREGQIYSLGPSGVAARERVSAPAAPRRACAENRCPGGPNALSADSGNICASWLRVTEQRKAPARESRKGPVRRAAGQASPGDGGQEGGNGGRDRVFVWLHLLFRMRKP | **none** |
| **ORF161** [132810 - 130978] (REVERSE SENSE) NTRIQQHIKKLIHHNQDSFIPGMQGWFNIRKSINVIHQINRTNDKNHMINSIDAEKTFNKIQQPFMLKTLNKLGIDGMYLKIVTAIYDKPTTNIILNGQKLEAFPLKTGTRQGCPVLPLLFNIVLEVLARAIRQEKEIKGIQLGNEEVKLSLFADDMIVYLENPIISAQNLLKLISNFSKVSGYQIDVQKSQAFLYIINRQTESQIISELPFTTATKRIKYLGIQLTRDVKDLFKENYKPLLNEIKEDTNKWKNIPCSWIGRINIMKMAILPKVIYRFNAIPIKLPMTFFTELEKTTLKFIWNQKRACIAKTILIKKNKAGGITLPDFKLYYKATVTKTAWYWYQNRYIDQWNRTEPSEITPHIYNYLIFDKPEKNKHWGKDSLFNKWCWENWLAICRKLKLDPFLTPYTKINSRWIKDLNVRPKTIKTLEEHLGSTIQDIGMGKDFMTKTPKAMATKAKLDRWDLIKLKSFCMAKETTISVNRQPTEWEKIFAIYPYDKGLISRIYKELKQIYKKKTNNPIKKWAKDINRHFSKEDIYAANRHMKKCSPSLAIREMQIKTTMRYHLTPVRMVIIKKSGHNRCWRGCGEIETLLHCWWECKLVQPLWKTVW | **none** |
| **ORF162** [120870 - 119776] (REVERSE SENSE) GHRPAPPSLFTDTSPGRPPRSPCPTPHRIDHQGLCLLRIPNAFSFLPADLRGGGGPGVFELDPSSLPVTPLLLPPPHSPLKRQSDLPECKPDQPHSLKSFDASHFSQGKTPPLLPTCVRGPCRTESPRSPRRHLPRRPSSFPGSPGPSSEPTRPLTPADPPADPSSPLRPVSPCGRSRGARGLRPLSLRPHPQGPPAQFLGQGWSSSAPGRKVRFPGSTVSKGCIPTRAPPLRQPRPLRQPRPLRQPRPSGSPAPPATPKSRARREPLRSLPPGGARGAGRRAGGECGAGRGERAGRVLLPDAQGAQRRGKGASPAGRQALGLALLAAESLGLATGGLRMTWDRPPLQRQLSQSAPLPRRTRPFA | **none** |
| **ORF163** [102056 - 100992] (REVERSE SENSE) RGVEGKWEGEGSKRLPRAGEQSQARAGLQRSTDQQCPSTPGAPGRLGVLCTALLSWGVWGCCVRPCYPGESGGAVYGLVILGSLGVLCTALLSWGVWGCSVRPCYPGESGGALYGLVILGSLGVLCTALLSWGVWGCSVRPCYPGESGGALYGLVILGSLGVLCTALLSWGVWGCCVRPCYPGESGGALYGLVILGSLGVLCTALLSWGVWGCSVRPCYPGESGGALYGLVILGSLGVLCTALLSWGVWGCSVRPCYPGESGGALYGLVILGSLGVLCTALLSWGVWGCSVRPCYPGESGGALYGLVILGSLGVLCTALLSWGVWGCSVRPCYPGESGGALYGLVVKGSSPLVPF | **none** |
| **ORF164** [102045 - 100855] (REVERSE SENSE) GKVGGGGEQEAAQSRGTEPGQGRIAKVHRPAMPKHTWRPREAGGALYGLVILGSLGVLCTALLSWGVWGCCVRPCYPGESGGALYGLVILGSLGVLCTALLSWGVWGCSVRPCYPGESGGALYGLVILGSLGVLCTALLSWGVWGCSVRPCYPGESGGALYGLVILGSLGVLCTALLSWGVWGCSVRPCYPGESGGALYGLVILGSLGVLCTALLSWGVWGCSVRPCYPGESGGALYGLVILGSLGVLCTALLSWGVWGCSVRPCYPGESGGALYGLVILGSLGVLCTALLSWGVWGCSVRPCYPGESGGALYGLVILGSLGVLCTALLSWGVWGCSVRPCCQRLLTLGSLLTWQWPQREAGRCYICQQQHCQHLVWMLPYSPINGCLPFPFHRREC | **none** |
| **ORF165** [102130 - 100631] (REVERSE SENSE) PSRLLSLDSSLLWCTPPSSTTAGGEGVLRESGRGRGARGCPEQGNRARPGPDCKGPQTSNAQAHLAPQGGWGCSVRPCYPGESGGAVYGLVILGSLGVLCTALLSWGVWGCSVRPCYPGESGGALYGLVILGSLGVLCTALLSWGVWGCSVRPCYPGESGGALYGLVILGSLGVLCTALLSWGVWGCSVRPCYPGESGGAVYGLVILGSLGVLCTALLSWGVWGCSVRPCYPGESGGALYGLVILGSLGVLCTALLSWGVWGCSVRPCYPGESGGALYGLVILGSLGVLCTALLSWGVWGCSVRPCYPGESGGALYGLVILGSLGVLCTALLSWGVWGCSVRPCYPGESGGALYGLVILGSLGVLCTALLSKAPHPWFPSDLAMAPEGGRQVLHLSAATLPTLSVDVTLFTHQRLSAIPISQARMLRLKKGSDMVMSTGQVADGTGTGGPVCHGIPNSWDLQGRPSFCAVCSEVPVGVRAERYRPTFTEMQTFLLLYMEG | **none** |
| **ORF166** [91654 - 90575] (REVERSE SENSE) GRTWKEGPARNGLDEGEGTVGEKLLIPLRSGCLGSSPSCPTPIREGGLFQAETAQSWCWDPGQPVVLRAPVTGWVSSAGMTGHRHTATVAAVVVQAEPLQTQAAIWPGSCPEAPPCSCCWDLHSPGPSSPAPGQQLQKVTGQEAPELWEEAAVGPFPGAAVFVGNIWPQSTSAVPAAAVGLGSLLTILSGVGGGVLEERTLLSLWDAGGEGDGPAHWEIPVLGEGSSCQGEDRTGVWGPQWVAGTSRCQGTRLGRLGGLLGDSQCKISKRSNHFKNSPQCHCLFHERVPCSLPHPQASATCWPCLMALLPSASSLNSFLHPCMAEPRMVDRYQELFLGEQWAGGTVPEGAPGGSWCRFAG | **none** |
| **ORF167** [15031 - 13670] (REVERSE SENSE) KMVQLLWKTVRQLLKKLNINLACRPVVPPLRIYPRATKTRSIHMRLHRRPQQHPHEDDADVHSSIHTRLRRRPQQHPHEDDADVHSSIHTRLRRRPQQHPHETTQTSTAASTRGRRRRPQQHPHETTQTSIAASTRGRRRRPQQHRGRRRRPQQHPHETTQTSTAASTRDYADVHSSIHTRTTQTSTAASTGGRRRRPQQHPHEDDADVHSSIHTRTTQTSTAASTRGRRRRPQQHPHETTQTSTAASTRGRRRRPQQHPHETTQTSTAASTRDDADVHSSIHTRRRRRPQQHPHEDDADVHSSIHTRTTQTSTAASTRGRRRRAQQHPHETTQTSTAASTRDYADVHSSIHTRLRRCPQQHPHEDDADVHSSIHTRLRRRPQQHPHEDDADVHSSIHTRTTQTSTAASTRGLRKCPQQQDSQKPQTGSNPDVHGQKSRQTKCGPCMQWNVLQP | **none** |
| **ORF168** [14987 - 13518] (REVERSE SENSE) KVKHKLSVSPSCSSATNLPQSNENSQHPHETTQTSTAASTRGRRRRPQQHPHETTQTSTAASTRGRRRRPQQHPHETTQTSTAASTRDYADVHSSIHTRTTQTSTAASTRDYADVHSSIHTRTTQTSTAASRTTQTSTAASTRDYADVHSSIHTRLRRRPQQHPHEDDADVHSSIHRRTTQTSTAASTRGRRRRPQQHPHEDDADVHSSIHTRTTQTSTAASTRDDADVHSSIHTRTTQTSTAASTRDDADVHSSIHTRRRRRPQQHPHETTQTSTAASTRGRRRRPQQHPHEDDADVHSSIHTRTTQTCTAASTRDYADVHSSIHTRLRRRSQQHPHETTQMSTAASTRGRRRRPQQHPHETTQTSTAASTRGRRRRPQQHPHEDDADVHSSIHTRTTQMSTAAGFTEAPNWKQPRCPWTEEQTNKMWSLHAMECASAMKWNEVLIQPAVRMARENSRRKPGDMHDIWSVWSAGEWLPGLGRSTGKGSD | **none** |
| **ORF169** [1494 - 64] (REVERSE SENSE) GKLPLGVWGPSPRATLDGSFCQFSFFMRSVGGFTRRWLGTHAEPKPWSTWATMTSSMPPATGGLQVQGSSGGGGGGGPQPIPGSRGGGGGGHPKPGSGGGGGGRNSQEPGRGGGRGGAGGAMAPRPGRGGGGGAVGRALAPTLGRGGGGGGGGGSALEPGSRGGGGGGGGVDACCSRALGCETFRMLSLWAWSTGSQAAAAAGCWPSTLGLGVVVFCGELPLWLWSRLAWTLLWALTRGLGLGRPLTDRSRSTTSSRVHSWAAVGDVQGQGRREPEREGWGLEEEAGKSGHLPWAEDGLGPRAGSPPHGLRPQCLPTRLCLRPCPAPSPSLARSTARFTRLSRASQSSWLERRVGSRCRRPCSTDRSWADTGEQLTWTGECGVQVSSRPLPGGLRTVACLPGCPAPSPPLFPRIPRDESFSDCGTGDMWDCPVHYGMLNRTTGLYLLNANSTPCPAVAAKNVFRCCQMPPGDTTAPR | **none** |
