# Supplementary material for: Genetic Determinants of Antibody Levels in Cerebrospinal Fluid in Multiple Sclerosis: Possible Links to Endogenous Retroviruses
Source: Int J Mol Sci. 2018 Mar 9;19(3):786. doi: 10.3390/ijms19030786 (PMC5877647; doi:10.3390/ijms19030786)
Supplement: Supplementary file 1 [file ijms-19-00786-s001.zip › Supplementary Table S4.docx]

**Supplementary Table S4. Genes, pseudogenes, and large retroviral ORFs flanking rs11621145 on chromosome 14**. Presented are all annotated genes and the identified ORFs in the two megabase pair region (nucleotide accession: NC_000014.9:104706543-106706543) surrounding rs11621145. Genes/ORFs located on the reverse strand are indicated by the orientation “c”). ORFs were numbered according to Supplementary Table S3.

| **Orientation** | **Start** | **Stop** | **Gene symbol** | **Feature** |
| --- | --- | --- | --- | --- |
|  | <1 | 13068 | INF2 |  |
|  | 13218 | 16001 | LOC388022 |  |
|  | 17655 | 40783 | ADSSL1 |  |
| c | 34602 | 35298 | LOC107984670 |  |
| c | 40870 | 60422 | LOC107987209 |  |
|  | 45878 | 53117 | SIVA1 |  |
|  | 61424 | 63586 | LOC102723342 |  |
| c | 62807 | 89201 | AKT1 |  |
|  | 94055 | 98170 | ZBTB42 |  |
|  | 114659 | 117176 | LINC00638 |  |
| c | 124294 | 124628 | RPS26P49 |  |
| c | 129402 | 130365 | RPS2P4 |  |
|  | 158771 | 190228 | CEP170B |  |
|  | 218264 | 231247 | PLD4 |  |
| c | 226814 | 227938 | - | **ORF148** |
|  | 279259 | 293768 | C14orf79 |  |
| c | 303031 | 314546 | CDCA4 |  |
|  | 342847 | 359008 | GPR132 |  |
|  | 387605 | 392954 | LOC102723354 |  |
|  | 434439 | 462282 | JAG2 |  |
| c | 444236 | 444322 | MIR6765 |  |
|  | 460741 | 461754 | - | **ORF30** |
| c | 466395 | 474781 | NUDT14 |  |
| c | 502744 | 609035 | BRF1 |  |
|  | 542000 | 544551 | BDPL |  |
|  | 594269 | 594269 | PACS2 |  |
| c | 662455 | 663462 | - | **ORF132** |
|  | 668079 | 668429 | RPS20P33 |  |
|  | 691957 | 707317 | TEX22 |  |
| c | 711039 | 713197 | LOC100507437 |  |
|  | 713307 | 764187 | MTA1 |  |
|  | 766396 | 773628 | CRIP2 |  |
|  | 774270 | 775268 | - | **ORF46** |
|  | 780378 | 782247 | CRIP1 |  |
|  | 783313 | 792706 | C14orf80 |  |
|  | 819463 | 820668 | - | **ORF47** |
|  | 820074 | 823660 | TMEM121 |  |
| c | 825313 | 827776 | LOC105370697 |  |
| c | 830204 | 830750 | ATP5G1P1 |  |
|  | 838881 | 843292 | ELK2BP |  |
| c | 841037 | 850861 | LOC105370698 |  |
| c | 879895 | 2000001> | IgH |  |
|  | 880395 | 881852 | IGHA2 |  |
|  | 893524 | 895185 | IgE |  |
| c | 914574 | 914638 | MIR8071-1 |  |
|  | 917934 | 919523 | IGHG4 |  |
| c | 933626 | 933690 | MIR8071-2 |  |
|  | 936661 | 938247 | IGHG2 |  |
| c | 948339 | 958083 | LOC105378187 |  |
|  | 953959 | 955257 | - | **ORF68** |
|  | 961707 | 963498 | IGHGP |  |
|  | 963035 | 966265 | ELK2AP |  |
|  | 1000001 | 1000001 | - | rs11621145 |
| c | 1000626 | 1002122 | IGHA1 |  |
| c | 1015252 | 1015985 | IGHEP1 |  |
|  | 1021590 | 1026715 | LOC107987211 |  |
| c | 1034931 | 1036528 | IGHG1 |  |
|  | 1045777 | 1059455 | LOC105378184 |  |
| c | 1059372 | 1064863 | IgG3 |  |
| c | 1077954 | 1079430 | ATP6V1G1P1 |  |
| c | 1131859 | 1131859 | IGHD |  |
| c | 1145424 | 1149675 | IGHM |  |
| c | 1150971 | 1151030 | MIR4539 |  |
| c | 1151582 | 1151633 | MIR4507 |  |
| c | 1151623 | 1151700 | MIR4538 |  |
| c | 1152942 | 1153011 | MIR4537 |  |
| c | 1156654 | 1156718 | IGHJ6 |  |
| c | 1156873 | 1156923 | IGHJ3P |  |
| c | 1157270 | 1157322 | IGHJ5 |  |
| c | 1157671 | 1157720 | IGHJ4 |  |
| c | 1158043 | 1158094 | IGHJ3 |  |
| c | 1158251 | 1158310 | IGHJ2P |  |
| c | 1158655 | 1158709 | IGHJ2 |  |
| c | 1158863 | 1158916 | IGHJ1 |  |
| c | 1159009 | 1159019 | IGHD7-27 |  |
| c | 1159082 | 1159135 | IGHJ1P |  |
| c | 1174492 | 1174511 | IGHD1-26 |  |
| c | 1174997 | 1175014 | IGHD6-25 |  |
| c | 1177361 | 1177380 | IGHD5-24 |  |
| c | 1178328 | 1178346 | IGHD4-23 |  |
| c | 1179489 | 1179519 | IGHD3-22 |  |
| c | 1182009 | 1182036 | IGHD2-21 |  |
| c | 1184649 | 1184665 | IGHD1-20 |  |
| c | 1185157 | 1185177 | IGHD6-19 |  |
| c | 1187000 | 1187019 | IGHD5-18 |  |
| c | 1187966 | 1187981 | IGHD4-17 |  |
| c | 1189092 | 1189128 | IGHD3-16 |  |
| c | 1191415 | 1191445 | IGHD3-15 |  |
| c | 1194096 | 1194112 | IGHD1-14 |  |
| c | 1194600 | 1194620 | IGHD6-13 |  |
| c | 1196107 | 1196129 | IGHD5-12 |  |
| c | 1197074 | 1197089 | IGHD4-11 |  |
| c | 1197955 | 1197985 | IGHD3-10 |  |
| c | 1198139 | 1198169 | GHD3-9 |  |
| c | 1200669 | 1200699 | IGHD2-8 |  |
| c | 1203365 | 1203381 | IGHD1-7 |  |
| c | 1203868 | 1203885 | IGHD6-6 |  |
| c | 1205715 | 1205734 | IGHD5-5 |  |
| c | 1206680 | 1206695 | IGHD4-4 |  |
| c | 1207817 | 1207847 | IGHD3-3 |  |
| c | 1210284 | 1210314 | IGHD2-2 |  |
|  | 1211437 | 1226100 | KIAA0125 |  |
| c | 1212960 | 1212976 | IGHD1-1 |  |
| c | 1233212 | 1233659 | IGHV6-1 |  |
| c | 1238396 | 1238849 | IGHVII-1-1 |  |
|  | 1263189 | 1265727 | ADAM6 |  |
| c | 1271926 | 1272627 | RPS8P1 |  |
| c | 1280040 | 1280477 | IGHV1-2 |  |
| c | 1294992 | 1295293 | IGHVIII-2-1 |  |
| c | 1298553 | 1298990 | IGHV1-3 |  |
|  | 1300117 | 1305420 | LOC107984634 |  |
| c | 1305380 | 1305814 | IGHV4-4 |  |
| c | 1331360 | 1331803 | IGHV2-5 |  |
| c | 1333124 | 1333222 | IGHVIII-5-1 |  |
| c | 1339058 | 1339318 | IGHVIII-5-2 |  |
| c | 1349002 | 1349456 | IGHV3-6 |  |
| c | 1355607 | 1356062 | IGHV3-7 |  |
| c | 1381541 | 1382031 | IGHV3-64D |  |
| c | 1410093 | 1410542 | IGHV3-11 |  |
| c | 1413747 | 1413933 | IGHVIII-11-1 |  |
| c | 1415878 | 1416167 | IGHV1-12 |  |
| c | 1422998 | 1423450 | IGHV3-13 |  |
| c | 1435744 | 1436042 | IGHVIII-13-1 |  |
| c | 1439340 | 1439589 | IGHV1-14 |  |
| c | 1447080 | 1447541 | IGHV3-15 |  |
| c | 1456992 | 1457262 | IGHVII-15-1 |  |
| c | 1458661 | 1459114 | IGHV3-16 |  |
| c | 1464204 | 1464509 | IGHVIII-16-1 |  |
| c | 1467800 | 1468236 | IGHV1-17 |  |
| c | 1472343 | 1473950 | SLC20A1P2 |  |
| c | 1478357 | 1478793 | IGHV1-18 |  |
| c | 1490156 | 1490448 | IGHV3-19 |  |
| c | 1491126 | 1492592 | - | **ORF87** |
| c | 1494859 | 1496148 | - | **ORF86** |
|  | 1495837 | 1501881 | LOC105370700 |  |
| c | 1496575 | 1497684 | - | **ORF85** |
| c | 1504394 | 1504849 | IGHV3-20 |  |
| c | 1506283 | 1506781 | IGHVII-20-1 |  |
| c | 1528520 | 1528973 | IGHV3-21 |  |
| c | 1551220 | 1551681 | IGHV3-22 |  |
| c | 1556818 | 1557086 | IGHVII-22-1 |  |
| c | 1558144 | 1558331 | IGHVIII-22-2 |  |
| c | 1562064 | 1562519 | IGHV3-23 |  |
| c | 1570004 | 1570441 | IGHV1-24 |  |
| c | 1579453 | 1581350 | HOMER2P2 |  |
|  | 1581132 | 1581830 | LINC00226 |  |
| c | 1582484 | 1582937 | GHV3-25 |  |
| c | 1587024 | 1587265 | IGHVIII-25-1 |  |
| c | 1594853 | 1595296 | IGHV2-26 |  |
| c | 1602864 | 1603170 | IGHVIII-26-1 |  |
| c | 1608106 | 1608418 | IGHVII-26-2 |  |
| c | 1611281 | 1611694 | IGHV7-27 |  |
| c | 1617712 | 1618146 | IGHV4-28 |  |
| c | 1622890 | 1623144 | IGHVII-28-1 |  |
| c | 1624564 | 1625021 | IGHV3-29 |  |
| c | 1625694 | 1626539 | GOLGA4P2 |  |
| c | 1628538 | 1628991 | IGHV3-30 |  |
| c | 1636138 | 1636411 | IGHVII-30-1 |  |
| c | 1637843 | 1638291 | IGHV3-30-2 |  |
| c | 1642741 | 1643178 | IGHV4-30-2 |  |
| c | 1647906 | 1648162 | IGHVII-30-21 |  |
| c | 1649603 | 1650060 | IGHV3-32 |  |
| c | 1650742 | 1651255 | GOLGA4P1 |  |
| c | 1653249 | 1653702 | IGHV3-33 |  |
| c | 1660849 | 1661122 | IGHVII-33-1 |  |
| c | 1662556 | 1663004 | IGHV3-33-2 |  |
| c | 1667119 | 1667551 | IGHV4-34 |  |
| c | 1670758 | 1671191 | IGHV7-34-1 |  |
| c | 1682848 | 1683301 | IGHV3-35 |  |
| c | 1686227 | 1686689 | IGHV3-36 |  |
| c | 1690123 | 1690572 | IGHV3-37 |  |
| c | 1703952 | 1704401 | IGHV3-38 |  |
| c | 1711476 | 1711768 | IGHVIII-38- |  |
| c | 1715167 | 1715605 | IGHV4-39 |  |
| c | 1718817 | 1719019 | IGHV7-40 |  |
| c | 1734392 | 1734468 | IGHVII-40-1 |  |
| c | 1736587 | 1737042 | IGHV3-41 |  |
| c | 1752400 | 1754764 | HOMER2P1 |  |
| c | 1754342 | 1755180 | LOC107984633 |  |
| c | 1756712 | 1757146 | IGHV3-42 |  |
| c | 1763721 | 1764178 | IGHV3-43 |  |
| c | 1766249 | 1766458 | IGHVII-43-1 |  |
| c | 1771675 | 1771851 | IGHVIII-44 |  |
| c | 1775897 | 1788973 | LINC00221 |  |
| c | 1782800 | 1783228 | IGHVIV-44-1 |  |
| c | 1787603 | 1787839 | IGHVII-44-2 |  |
| c | 1800454 | 1800891 | IGHV1-45 |  |
| c | 1804573 | 1805010 | IGHV1-46 |  |
| c | 1809294 | 1809582 | IGHVII-46-1 |  |
| c | 1812037 | 1812492 | IGHV3-47 |  |
| c | 1824591 | 1824894 | IGHVIII-47-1 |  |
| c | 1831268 | 1831723 | IGHV3-48 |  |
| c | 1850394 | 1850855 | IGHV3-49 |  |
| c | 1857786 | 1858056 | IGHVII-49-1 |  |
| c | 1859558 | 1860013 | IGHV3-50 |  |
| c | 1872200 | 1872635 | IGHV5-51 |  |
| c | 1876959 | 1877265 | IGHVIII-51-1 |  |
| c | 1878172 | 1878434 | IGHVII-51-2 |  |
| c | 1879834 | 1880284 | IGHV3-52 |  |
| c | 1886134 | 1886584 | IGHV3-53 |  |
| c | 1893113 | 1893382 | IGHVII-53-1 |  |
| c | 1894794 | 1895250 | IGHV3-54 |  |
| c | 1899574 | 1900009 | IGHV4-55 |  |
| c | 1903220 | 1903654 | IGHV7-56 |  |
| c | 1912367 | 1912672 | IGHV3-57 |  |
| c | 1915815 | 1916252 | IGHV1-58 |  |
| c | 1920707 | 1921138 | IGHV4-59 |  |
| c | 1924655 | 1925111 | IGHV3-60 |  |
|  | 1930030 | 1930319 | RNA5SP389 |  |
| c | 1931163 | 1931431 | IGHVII-60-1 |  |
| c | 1932577 | 1933014 | IGHV4-61 |  |
| c | 1936588 | 1937043 | IGHV3-62 |  |
| c | 1943974 | 1944246 | IGHVII-62-1 |  |
| c | 1945671 | 1946138 | IGHV3-63 |  |
| c | 1946924 | 1947840 | GOLGA4P3 |  |
| c | 1951181 | 1951636 | IGHV3-64 |  |
|  | 1951353 | 1959853 | LOC102724977 |  |
| c | 1959496 | 1959990 | IGHV3-6 |  |
| c | 1965230 | 1965502 | IGHVII-65-1 |  |
| c | 1968473 | 1968923 | IGHV3-66 |  |
| c | 1974061 | 1974500 | IGHV1-67 |  |
| c | 1976088 | 1977807 | SLC20A1P1 |  |
| c | 1979851 | 1980000 | IGHVII-67-1 |  |
| c | 1980560 | 1980658 | IGHVIII-67-2 |  |
| c | 1986072 | 1986346 | IGHVIII-67-3 |  |
| c | 1988550 | 1988855 | IGHVIII-67-4 |  |
| c | 1997265 | 1997744 | IGHV1-68 |  |
