# Supplementary material for: Genetic Determinants of Antibody Levels in Cerebrospinal Fluid in Multiple Sclerosis: Possible Links to Endogenous Retroviruses
Source: Int J Mol Sci. 2018 Mar 9;19(3):786. doi: 10.3390/ijms19030786 (PMC5877647; doi:10.3390/ijms19030786)
Supplement: Supplementary file 1 [file ijms-19-00786-s001.zip › Supplementary Table S5.docx]

**Supplementary Table S5. Large ORFs surrounding rs9271640/rs6457617 on human chromosome 6**. The 2,071,651 base pairs flanking the rs9271640/ rs6457617 region were analyzed using getorf for the presence of open reading frames with a minimal length of one kilobase. 27 open reading frames were identified. These ORFs were analyzed using BLASTP against the database of retro-transcribing viruses (taxid 35268).

| **ORF number [position in sequence]**  **sequence** | **Representative**  **BLASTP hits** |
| --- | --- |
| **ORF1** [6646 - 8601] SVLFPPSSGFPEILISILSVCLFRSETPPVPPPPPYLASYPGFPENGAPGPPISRFPLEEPGPRPLPWPPGSDEVAKIQTPPPKKEPPKEETAQLTGPEAGRKPARGVGSGGQGPPPPRRESRTETRWGPRPGSSRRGIPPEEPGAPPRRAGPIKKPPPPTKVEELPPKPLEQGDETPKPPKPDPLKITKGKLGGPKETPPNGNLSPAPRLRRDYSYERVGPTSCRGRGRGEYFARGRGFRGTYGGRGRGARSREFRSYREFRGDDGRGGGTGGPNHPPAPRGRTASETRSEGSEYEEIPKRRRQRGSETGSETHESDLAPSDKEAPTPKEGTLTQVPLAPPPPGAPPSPAPARFTARGGRVFTPRGVPSRRGRGGGRPPPQVCPGWSPPAKSLAPKKPPTGPLPPSKEPLKEKLIPGPLSPVARGGSNGGSNVGMEDGERPRRRRHGRAQQQDKPPRFRRLKQERENAARGSEGKPSLTLPASAPGPEEALTTVTVAPAPRRAAAKSPDLSNQNSDQANEEWETASESSDFTSERRGDKEAPPPVLLTPKAVGTPGGGGGGAVPGISAMSRGDLSQRAKDLSKRSFSSQRPGMERQNRRPGPGGKAGSSGSSSGGGGGGPGGRTGPGRGDKRSWPSPKNRRWVGTNKFIVV | **none** |
| **ORF2** [191086 - 193257] RLIKAQGQAVRITASLRSCCDSPLPFSRVTPVVPRLPRANLCGCRHRRVEFPASGRTELFSRIQCSVSSPQSQSGADREQGTGMAKAAAIGIDLGTTYSCVGVFQHGKVEIIANDQGNRTTPSYVAFTDTERLIGDAAKNQVALNPQNTVFDAKRLIGRKFGDPVVQSDMKHWPFQVINDGDKPKVQVSYKGETKAFYPEEISSMVLTKMKEIAEAYLGYPVTNAVITVPAYFNDSQRQATKDAGVIAGLNVLRIINEPTAAAIAYGLDRTGKGERNVLIFDLGGGTFDVSILTIDDGIFEVKATAGDTHLGGEDFDNRLVNHFVEEFKRKHKKDISQNKRAVRRLRTACERAKRTLSSSTQASLEIDSLFEGIDFYTSITRARFEELCSDLFRSTLEPVEKALRDAKLDKAQIHDLVLVGGSTRIPKVQKLLQDFFNGRDLNKSINPDEAVAYGAAVQAAILMGDKSENVQDLLLLDVAPLSLGLETAGGVMTALIKRNSTIPTKQTQIFTTYSDNQPGVLIQVYEGERAMTKDNNLLGRFELSGIPPAPRGVPQIEVTFDIDANGILNVTATDKSTGKANKITITNDKGRLSKEEIERMVQEAEKYKAEDEVQRERVSAKNALESYAFNMKSAVEDEGLKGKISEADKKKVLDKCQEVISWLDANTLAEKDEFEHKRKELEQVCNPIISGLYQGAGGPGPGGFGAQGPKGGSGSGPTIEEVD | **none** |
| **ORF3** [203370 - 205451] LPRSQGFPERTCAAAGTGVLSFRRSEGLSSCRGSRPPFPAPSLRAEPTEQGTGMAKAAAIGIDLGTTYSCVGVFQHGKVEIIANDQGNRTTPSYVAFTDTERLIGDAAKNQVALNPQNTVFDAKRLIGRKFGDPVVQSDMKHWPFQVINDGDKPKVQVSYKGETKAFYPEEISSMVLTKMKEIAEAYLGYPVTNAVITVPAYFNDSQRQATKDAGVIAGLNVLRIINEPTAAAIAYGLDRTGKGERNVLIFDLGGGTFDVSILTIDDGIFEVKATAGDTHLGGEDFDNRLVNHFVEEFKRKHKKDISQNKRAVRRLRTACERAKRTLSSSTQASLEIDSLFEGIDFYTSITRARFEELCSDLFRSTLEPVEKALRDAKLDKAQIHDLVLVGGSTRIPKVQKLLQDFFNGRDLNKSINPDEAVAYGAAVQAAILMGDKSENVQDLLLLDVAPLSLGLETAGGVMTALIKRNSTIPTKQTQIFTTYSDNQPGVLIQVYEGERAMTKDNNLLGRFELSGIPPAPRGVPQIEVTFDIDANGILNVTATDKSTGKANKITITNDKGRLSKEEIERMVQEAEKYKAEDEVQRERVSAKNALESYAFNMKSAVEDEGLKGKISEADKKKVLDKCQEVISWLDANTLAEKDEFEHKRKELEQVCNPIISGLYQGAGGPGPGGFGAQGPKGGSGSGPTIEEVD | **none** |
| **ORF4** [276760 - 278160] GCRPQWSRRRTAPLPGAGSCSVALRPRGRAAGSAGLPPQRPLWRGWATLVGKETGQRQRSLALRSQGSRTLAPILAQPPGIRSPGLQISSSVSPTLGRCCPSERREAWFSGAGQRRPHPPAQQPARMGRARAPTVEGRRKRGARGLGLWTPRWPRLQALLTPPPLPAASFLRPPLTRLHALFTSSPPPPARTVHALPPRSELRGTPPQPHDPECTLLSPGPVPRAVFLIPYFPHPPPGPAATPTHGQGLGSAQARGCTGRARARGRLRVGVRGEGRGRLVPCVPGPARAAAAASARTRGGGRGEGIKGAGSPLPDPPFFTSTPTRLKGRTALPPLWPGLFVRQSGNTFHTPLFLAPPPLYLQAPRPVCACVPSAARRPAHSGPPPNPGCPWRYSLRVPAPRPRRIPQRARAPVLSAAGAATCSQGWCVSGPAVPSAALGASVAPLRAPSAPWLTELLVAITHACLLC | **none** |
| **ORF5** [428419 - 429456] RSRSGPGGWSRRGSASQGRILSLSAPPGAAPQGAPGPPCWVLWGREFLPLQLRRGDREGEAASLGDVLGSCEEGERSCGHEWGSWGQLGEAHLWSWVVLLSLQSTHMTSSEVSAGELGTATNRAHRALLHPGRSVSPQGSGERQKSTMAQPRAEGLPGPVHPSPEKGRPSHRPHSGAPIVHSPVTPMTEMGPTRWPPWKPYRFILYLWSGSRPEMVTPSSCPGTRTTLGCPSPFLYWTRKWSNWPSGTIQDRLRESGVASVTVSSPRRGLLGGSGVVVGTAWVVSASSSGAGQTRVGRVRSLGSQFSIERMCHKTQSAQEQDDAAHSASSTALHPLLSCGLSYPSP | **none** |
| **ORF6** [462785 - 463810] GWTRDRCPVAPAHTTCGGDEGVGLGGLPRPHPVDLHVVGGAGPEVGQADGARGARHSQLTAGALCRRLRPPAQHHALELAAEPVERHEKRAIGGTAQHLWLGVAGRSHPVDAFRREAVDSLVGVPFPGAGIRAGGGAGVAGRGCGGAGWGGGQQTPLQVFMDVARHRRVSLVGPQWVAVSPQVALQVLHRAAETAQLGRQVAPRPPWGLGQAWPGRGLLLPFLCWPRGPRGPWGPRGWGWPGPGLGGRGWGAHLRVTVVAWCCQGQKRGEQVSGSSLPALLPAAAPPSIPPTRARGLFPVPQPHLERERREIGQCPAAPPFPSKPTTVGALERREKPVGGAL | **none** |
| **ORF7** [471879 - 473033] SPGCTRSGHPRSARARDSRQDRRACRSPGLCTPANTCSGHSHSGPGHDSPQDSCAHRSPRRCSPHCKHTRRSRSARDLYSRVGRRASRNRFQCTPASTRSGRPRSGPFGCSRQGSCARRSRPRCSPCRKCIRRPRSARGPGSPSDMSACCSPRLCTRASTRSGRPRSARPRHSPRGSSAYHSPRQCSRGHTRSARPCSVPAGCSPEGSSSPHSPRQCSRGYTRSAHSHSAPSDCNREGSSSCHSRHQCSLGHTRSAHPHSVPSGCNLEGRSAGCSRGLRSLPGTHTRPARSAHGLGSPRDRMASHSWGQCSRESTRNDHGHSDPDHCSHLGRTPRLRVGKREGSQHLWGLSRWAHPSLAHRNRCSGSDQTHHGDCRGSAHLSDLE | **none** |
| **ORF8** [1652953 - 1654131] SPPFALTDPLGVRRTMQLRLFRRLLLAALLLVIVWTLFGPSGLGEELLSLSLASLLPAPASPGPPLALPRLLIPNQEACSGPGAPPFLLILVCTAPENLNQRNAIRASWGGLREARGLRVQTLFLLGEPNAQHPVWGSQGSDLASESAAQGDILQAAFQDSYRNLTLKTLSGLNWAEKHCPMARYVLKTDDDVYVNVPELVSELVLRGGRWGQWERSTEPQREAEQEGGQVLHSEEVPLLYLGRVHWRVNPSRTPGGRHRVSEEQWPHTWGPFPPYASGTGYVLSASAVQLILKVASRAPLLPLEDVFVGVSARRGGLAPTQCVKLAGATHYPLDRCCYGKFLLTSHRLDPWKMQEAWKLVGGSDGERTAPFCSWFQGVLGILRCRAIAWLQS | **none** |
| **ORF9** [1817856 - 1819607] VVPVLHLLLIILMMALLFPLPVSSSMTPYPSHHSRLSFHLSTSLSFSVCARPSFHLSPAPSTFSPSWLEASTGEGLSPPPPSSPLLSVPSPTPLAFALLLSSPPPWTSSPPYVPSPAPIPSLAAPISPPLGLTPFPEGPCPFPLLTCPLPSSLPALFRAATASSQPFPLGPTFHPKACARPQQGSIARSPDLTFCLCAPFPSDSSMDMARLPSPTKEKPPPPPPGGGKDLFYVSRPPLARSSPAYCTSSSDITEPEQKMLSVNKSVSMLDLQGDGPGGRLNSSSVSNLAAVGDLLHSSQASLTAALGLRPAPAGRLSQGSGSSITAAGMRLSQMGVTTDGVPAQQLRIPLSFQNPLFHMAADGPGPPGGHGGGGGHGPPSSHHHHHHHHHHRGGEPPGDTFAPFHGYSKSEDLSSGVPKPPAASILHSHSYSDEFGPSGTDFTRRQLSLQDNLQHMLSPPQITIGPQRPAPSGPGGGSGGGSGGGGGGQPPPLQRGKSQQLTVSAAQKPRPSSGNLLQSPEPSYGPARPRQQSLSKEGSIGGSGGSGGGGGGGLKPSITKQVGEGRRKAGWVTTGREEGDGGWG | **none** |
| **ORF10** [1830586 - 1832097] PPSFSSAAFIPRGVYPQALLLFPILVTFEEAMETPTPLPPVPASPTCNPAPRTIQIEFPQHSSSLLESLNRHRLEGKFCDVSLLVQGRELRAHKAVLAAASPYFHDKLLLGDAPRLTLPSVIEADAFEGLLQLIYSGRLRLPLDALPAHLLVASGLQMWQVVDQCSEILRELETSGGGISARGGNSYHALLSTTSSTGGWCIRSSPFQTPVQSSASTESPASTESPVGGEGSELGEVLQIQVEEEEEEEEDDDDEDQGSATLSQTPQPQRVSGVFPRPHGPHPLPMTATPRKLPEGESAPLELPAPPALPPKIFYIKQEPFEPKEEISGSGTQPGGAKEETKVFSGGDTEGNGELGFLLPSGPGPTSGGGGPSWKPVDLHGNEILSGGGGPGGAGQAVHGPVKLGGTPPADGKRFGCLCGKRFAVKPKRDRHIMLTFSLRPFGCGICNKRFKLKHHLTEHMKTHAGALHACPHCGRRFRVHACFLRHRDLCKGQGWATAHWTYK | **none** |
| **ORF11** [1804274 - 1802916] (REVERSE SENSE) QEVSPSQGYACSGSEVTWTSKAADCPTSPWLAQQPPRQETGRGKIRQDTLAQGWLHMCARGTGPPCAHGTDTQETRAWARTHTRTHGLPLPPGAPRSHTHRPPPRAQDPVRTRPVPDPPVLRCPPPVTITPPTRKMQREHAEGAAAAAAAATAASPPPSPRPRTRAPPPHARGAPPSTRAPSPPGPALTSHRPPGGPGVSPVGEGPRPCAGEGRGAEGEGAVRPGSRRPARWGRRRGPGRPGRPRGDGRGNGSLRRRSRRCRRCPGPAAVARRPLLPWPPLSRALSPGDSARETGEGGKGTPKTNQATHHPLLSRTPPHTQNRGENEGSRLHPAEEEPRIPMPLKGPATSPGLESPQPQQTPWHCQVPKVPTTPMSTAHPPCPLHIPCLPEDPKRSPPSTFTKPSPQIHMPLEPLPAPTEPQYSNLHMPPQLQTHTVSPPKTHGRPPPETPRP | **none** |
| **ORF12** [1796679 - 1795546] (REVERSE SENSE) SEDSIKTPEVTFYWSRAFKRVRVWGRILTCPLLPSFHLTCTATPPSTHNLALPLPWSPLPHRSLSLLSSLHLDPPSPHPPLPPSLSIWPLLSLHPPLFFRPLPYSESPTKLSPHIFLASLFTPMFSFLLPSPSPKTLSLYFLDVFLITQLPPPNYFCSFFPNPILLPSHPFLALSFPFIIFPSAPFQGPFPGPSIPYLPLSLWPPIPPSRLLPPLPSRSSPLPPFQPLPSLGPDSPPPPAPPPVAPHMALPLPPPRTSEGGIGHRGDAPPMDGGSRPAHQAWGEGAGGRGRKKRERGGERGGEGGGGGGGGGGERRREEQREAERRREEEEEENKSQRQQRQPRERGGGGASESEESRRRRRRERLCSPHPYSGRPRL | **none** |
| **ORF13** [1786987 - 1785725] (REVERSE SENSE) TTSLSAPGGGRVPVPVSTPQDGKSEKGLVSAQPGPEAKAARLSRPRPAPTPGSLPPTSASCRLLTGDSWGRREPGRGRRRRPRRVPRAERARPGVGGRAGGGRGVPRPSPSVPAALGRMTQQPKQRQRQEEEAAGGREGGEGRTGWGGGWWEGRTGGKEEPEGGAGTTSPHLPHLQQAWRAPKKRLALRGLGSEESDPVGEGIPSAPSSDLRFALGARGNPPSRQPASGLVPRPMPHPLAAPPPMVTPSPAPRPQFTAPPSIHPSRCPHHALLPRAPRPPRDESRPTHRPFAHACPLLRAAPGRTEPLLHRAAAVGGWREGVGGRARRFPWTSRPVLPGLAQTKGARTQGGDATESQPMGSERAEPQPFERKGRERAGREERANSLGARASPASGLWGPVPICRHPPKERSSLGLGWPAEG | **none** |
| **ORF14** [1692599 - 1690593] (REVERSE SENSE) DVIIILFSFSSQRPPRPPSSFLCSCGWGGIPSLHNMEPSPLSPSGAALPLPLSLAPPPLPLPAAAVVHVSFPEVTSALLESLNQQRLQGQLCDVSIRVQGREFRAHRAVLAASSPYFHDQVLLKGMTSISLPSVMDPGAFETVLASAYTGRLSMAAADIVNFLTVGSVLQMWHIVDKCTELLREGRASATTTITTAAATSVTVPGAGVPSGSGGTVAPATMGSARSHASSRASENQSPSSSNYFSPRESTDFSSSSQEAFAASAVGSGERRGGGPVFPAPVVGSGGATSGKLLLEADELCDDGGDGRGAVVPGAGLRRPTYTPPSIMPQKHWVYVKRGGNCPAPTPLVPQDPDLEEEEEEEDLVLTCEDDEDEELGGSSRVPVGGGPEATLSISDVRTLSEPPDKGEEQVNFCESSNDFGPYEGGGPVAGLDDSGGPTPSSYAPSHPPRPLLPLDMQGNQILVFPSSSSSSSSQAPGQPPGNQAEHGAVTVGGTSVGSLGVPGSVGGVPGGTGSGDGNKIFLCHCGKAFSHKSMRDRHVNMHLNLRPFDCPVCNKKFKMKHHLTEHMKTHTGLKPYECGVCAKKFMWRDSFMRHRGHCERRHRLGGVGAVPGPGTPTGPSLPSKRESPGVGGGSGDEASAATPPSSRRVWSPPRVHKVEMGFGGGGGAN | **none** |
| **ORF15** [936583 - 935582] (REVERSE SENSE) HSFNVTFVRNITTQFTACVFACKSCQLYHCINHSTLQTHNISTLIILGCIPGLWIPVNLSKPWAATPALHYVKLFLTQLTHHGHRALRMIIFAIVSLVTLITSVVMSSAAFHSSIQTAHYMENWMPMADQAWLLQNKINTELQTEVAMLKSTVVWLEEQVQSLQLQQQLLCHLNHPHICVTKWLEYNQSEYPWDLVKVHLQGAFTSNITFDIGELQNKIFDLNKQTQEFQPSLEDWTEFQQGLESLNPWTYLKHYVNISFVVLEMMLFCLCLLFIVCKTGWTANGKMRAAQPGLTFFQLIHKQKEGNIESQRLENCHQFSIPQRLYDQTANCLS | **env protein [HERVK]**  **Sequence ID: CAB56604.1**  **E value: 1e-38** |
| **ORF16** [844979 - 843396] (REVERSE SENSE) QTESQIMSELPFTIASKRIKYLRIQLTRDVKDLFKENYKLLLNEIKEDTNKWKNIPCSWVGRINIMKMAILPKVIYRFNAILIKLPMTFFTELEKTTLKFIWNQKRALIAKSILSQKNKAGGIMLPDFKLYYKATVTKTAWYWYQNRDIDQGNRTEPSEIMPHIYNYLISDKPYKNKKWGKDSLFNKWCWENWLAICRKLKLDPFLTPYTKINSRWSKDLNVRPKTIKTLEENLGNTIQDIGMGKDFMSKTPKAMATKAKMDKWDLIKLKSFCTAKETTIRVNRQPTEWEKMFPIYSSDKGLISRNYKKLKQIYKKKTNNRSNMWVKDMNRHFSKEDIYAAKRHMKKCSSTLAIREMQIKTTMRYHLTPVRMEIIKKSGNNRCWRGCGEIGTLLHCWWGCKLVQPLWKSVWRFLNDLELEIPFDPAIPLLDIYPKDYKSCCYKDTCTCMFIVALFTIAKTWNQPKCPTMIGWIKKMWHIYTMEYYAAIKKDKFVSFAGTWMKLETIILNKLLQGQKTKHRMFSLIGGN | **none** |
| **ORF17** [669704 - 668562] (REVERSE SENSE) DKKGTSIKGKKIEIKRKYLISKSRPFVVCSVILTGYMDEELAKKSCSKIQILKCGGTARSQNSREENKEALKNDIIFTNSVESLKSAHIKEPEREGKGTDLEKDKIGMEVKVDSDAGIPKRQETQLKISEMSIPQGQGAQIKKSVSDVPRGQESQVKKSESGVPKGQEAQVTKSGLVVLKGQEAQVEKSEMGVPRRQESQVKKSQSGVSKGQEAQVKKRESVVLKGQEAQVEKSELKVPKGQEGQVEKTEADVPKEQEVQEKKSEAGVLKGPESQVKNTEVSVPETLESQVKKSESGVLKGQEAQEKKESFEDKGNNDKEKERDAEKDPNKKEKGDKNTKGDKGKDKVKGKRESEINGEKSKGSKRAKANTGRKYNKKVEE | **none** |
| **ORF18** [526872 - 525799] (REVERSE SENSE) AHINTGLHVYAYAHTGLARIGQPKGSYSKGEGIRSAGESWVHGDEKDRKEADQSCDLKTRLEGRRRGADGLDTAPLLPLKLEMVSHPSLSMCLSHYLSPSLTGCVSPYPLLNSSLCHLSSYTLLHSPPPGLPDSVPHTSPPPYNAPQPPAEPPAPPPQAAPSSHHHHHHHYHQSGTATLPRLGAGGLASSAATAQRGPSSSATLPRPPHHAPPGPAAGAPPPGCATLPRMPPDPYLQETRFEGPLPPPPPAAAAPPPPAPAQTAQAPGFVVPTHAGTVGTLPLGGYVAPGYPLQLQPCTAYVPVYPVGTVSAGQTGTWERGGRDTGLEIGDTLGAGRIEEQGQGTGSVPGTSPRKKGA | **none** |
| **ORF19** [505454 - 504312] (REVERSE SENSE) HCLVFFSGADLNLVHPFLTKTVHSPWKGLSIHMETPPVNTIGEKDTSQPQQEWEKNLRENLDSVIQIRQQPRDPPTETLELEVSPDPASQILEHTQGAEKLVAELEGDSHKSHGSTSQMPEALQASDLWYCPDGSFVKKIVIRGHGLDKPKLGSCCRVLALGFPFGSGPPEGWTELTMGVGPWREETWGELIEKCLESMCQGEEAELQLPGHSGPPVRLTLASFTQGRDSWELETSEKEALAREERARGTELFRAGNPEGAARCYGRALRLLLTLPPPGPPERTVLHANLAACQLLLGQPQLAAQSCDRVLEREPGHLKALYRRGVAQAALGNLEKATADLKKVLAIDPKNRAAQEELGKVVIQGKNQDAGLAQGLRKMFG | **none** |
| **ORF20** [473130 - 471004] (REVERSE SENSE) DEASLARGLHIGALHRLSLSPRRETETCLSPPTPGQTDVRTLCSLHGVFDLSRCTCSCEPGWGGPTCSDPTDAEIPPSSPPSASGSCPDDCNDQGRCVRGRCVCFPGYTGPSCGWPSCPGDCQGRGRCVQGVCVCRAGFSGPDCSQRSCPRGCSQRGRCEGGRCVCDPGYTGDDCGMRSCPRGCSQRGRCENGRCVCNPGYTGEDCGVRSCPRGCSQRGRCKDGRCVCDPGYTGEDCGTRSCPWDCGEGGRCVDGRCVCWPGYTGEDCSTRTCPRDCRGRGRCEDGECICDTGYSGDDCGVRSCPGDCNQRGRCEDGRCVCWPGYTGTDCGSRACPRDCRGRGRCENGVCVCNAGYSGEDCGVRSCPGDCRGRGRCESGRCMCWPGYTGRDCGTRACPGDCRGRGRCVDGRCVCNPGFTGEDCGSRRCPGDCRGHGLCEDGVCVCDAGYSGEDCSTRSCPGGCRGRGQCLDGRCVCEDGYSGEDCGVRQCPNDCSQHGVCQDGVCICWEGYVSEDCSIRTCPSNCHGRGRCEEGRCLCDPGYTGPTCATRMCPADCRGRGRCVQGVCLCHVGYGGEDCGQEEPPASACPGGCGPRELCRAGQCVCVEGFRGPDCAIQTCPGDCRGRGECHDGSCVCKDGYAGEDCGEGEQAAFPSVLWDCDSVNRSHGEDLSLRKSGGRACHSKEPVPTRGSRTTGVGLSSGQDWPSDL | **none** |
| **ORF21** [463774 - 462542] (REVERSE SENSE) GTNSGGLGGEWGGCGTLTDFPPFSFQVGLGHREEASGSGGRDRGRGCRGKECREGAATDLFSPFLPLAAPGHYSYPEVRPPAPPPKSRPRPAPAPRPPRPPWPSRPAEEREEESPPRPSLSQPPRRPWGNLTAELSRFRGTVQDLERHLRAHGYPLRANQTYTSVARHIHEYLQRRLLAAAPAGSPAPPPRHPRPTASPDPGTRKRDSNQGIYGLSPEGVDRVAASRHPKPEVLGSSADGALLVSLDGLRGQFERVVLRWRPQPPAEGPGGELTVPGTTRTVSLPDLRPGTTYHVEVHGVRAGQTSKSYAFITTTGSVGWGHGTPIPCPASPAVGACIHEFLPLPPPTAPFYLQALPGRGSRGPPGQSTSPLSLEDVPTPGSWHPSCGCLRSRQRLLWGFPGHFQDCYFRQ | **none** |
| **ORF22** [445051 - 443906] (REVERSE SENSE) ASHFISFPLIQKVSLCPFRVSCPSPSLQQPLSAFDHPGDSILTLHVMESQYAVSCVSFLAISTMLLKFTHVQQKLVFRLLTACPHSLSSLTPPESLGAATHQPSVSTCLCEPAGREETETETTAPTPPAPEPHLGELTVEEATSHTLHLSWMVTEGEFDSFEIQYTDRDGQLQMVRIGGDRNDITLSGLESDHRYLVTLYGFSDGKHVGPVHVEALTGENSAHYASFQMAGRVQRTAESRGYPAHLSPLFPCLCPVPEEEKPSEPPTATPEPPIKPRLGELTVTDATPDSLSLSWTVPEGQFDHFLVQYRNGDGQPKAVRVPGHEEGVTISGLEPDHKYKMNLYGFHGGQRMGPVSVVGVTGEWMMGAPGWEPWEGHPLALW | **none** |
| **ORF23** [387540 - 386371] (REVERSE SENSE) RNGGREGGGRDVLWVTKLVCFCLQSLLPHPLRAGLAPAGQTSEESRPRLSQLSVTDVTTSSLRLNWEAPPGAFDSFLLRFGVPSPSTLEPHPRPLLQRELMVPGTRHSAVLRDLRSGTLYSLTLYGLRGPHKADSIQGTARTLSPGKDPHTLCPKVGVFVLHGGDLVPQPAVGVGELVVGLEESAERLPFLGTRGKGVVSLCWSRGEGGTRREEPPCPCLRHCSLTPLQFWRAPVTSNSVKSGRPQPRSTGCPHHPGRTASKSPTSWRTEVVPLPCAHRLAFPLPPALCPPSSRPGVPWVTLDPQRFQGSLRVCRWTAGPGPRNSRWESRKRHGWELRENEEGEREVVEAPIADGRPVESGEARSRESRGDPSEPLPFPQFLTVPHSCVH | **none** |
| **ORF24** [277132 - 275507] (REVERSE SENSE) RAAGLGGVDAAAPPPRTTPPSSQRDSTSPGLGKLRKKSEDREIGCRGAGQEWGRGSWSLGFGEPETFVSDRFPSQPGLPTPATMASGVEVLRFQLPGHEAATLRNMNQLRAEERFCDVTIVADSLKFRGHKVILAACSPFLRDQFLLNPSSELQVSLMHSARIVADLLLSCYTGALEFAVRDIVNYLTAASYLQMEHVVEKCRNALSQFIEPKIGLKEDGVSEASLVSSISATKSLLPPARTPKPAPKPPPPPPLPPPLLRPVKLEFPLDEDLELKAEEEDEDEDEDVSDICIVKVESALEVAHRLKPPGGLGGGLGIGGSVGGHLGELAQSSVPPSTVAPPQGVVKACYSLSEDAEGEGLLLIPGGRASVGATSGLVEAAAVAMAARGAGGSLGAGGSRGPLPGGFSGGNPLKNIKCTKCPEVFQGVEKLVFHMRAQHFIFMCPRCGKQFNHSSNLNRHMNVHRGVKSHSCGICGKCFTQKSTLHDHLNLHSGARPYRCSYCDVRFAHKPAIRRHLKEQHGKTTAENVLEASVAEINVLIR | **none** |
| **ORF25** [205136 - 203289] (REVERSE SENSE) HPLALHLVLRFVLLRLLHHALDLLLAQAALVVGDGDLVGLAGALVRGRDVQDAVGIDVEGHLDLGHAPGGRRDAAQLEAPQQIVVFRHGPLALVHLDQHPGLVVGVGGEDLRLLGGDGGVALDQGSHHASGRLQPQRQGSHVQQQQVLHVLGLVPHQDGRLHRRPVGHSLVGVDALVQVAPVEEVLQQLLHLGDAGGAPDQDQVVNLGLVQLGVAQSLLHGLQGASEQVGAQLLEPRPGDGRVEVDALKQGVDLQAGLGAGRQGPLGPLAGGAQPPHGSLVLADVLLVFSLELLHEVVHQPVVKVLPTQVGVPGRGLHLEDAVVDRQDGHVEGAPAQVKDEHVALPLARSVQAVGDGGGRGLVDDPQHVEPRDHTRILGGLALRVVEVGRHGDHRVGHRVAQVRLGDLLHLGQHHGRDLLGVECLGLPLVAHLHLGLVSVVDHLERPVLHVRLHHRVAELAANQPLRVKHGVLRVQRHLVLGRIPDEPLGVREGHVAGGGAVALVVGDDLHLAVLEHPHAGVGGAQVDADRRGFGHAGALLCGLRSETGGWKRRTGSATRAQSFGTPETQHAGACSRTGSLWKALGPRESLSKDEADPRSSSSGWPFSGPRAPRLL | **none** |
| **ORF26** [192942 - 191158] (REVERSE SENSE) HPLALHLVLRFVLLRLLHHALDLLLAQAALVVGDGDLVGLAGALVRGRDVQDAVGIDVEGHLDLGHAPGGRRDAAQLEAPQQIVVFRHGPLALVHLDQHPGLVVGVGGEDLRLLGGDGGVALDQGSHHASGRLQPQRQGSHVQQQQVLHVLGLVPHQDGRLHRRPVGHSLVGVDALVQVAPVEEVLQQLLHLGDAGGAPDQDQVVNLGLVQLGVAQSLLHGLQGASEQVGAQLLEPRPGDGRVEVDALKQGVDLQAGLGAGRQGPLGPLAGGAQPPHGSLVLADVLLVFSLELLHEVVHQPVVKVLPTQVGVPGRGLHLEDAVVDRQDGHVEGAPAQVKDEHVALPLARSVQAVGDGGGRGLVDDPQHVEPRDHTRILGGLALRVVEVGRHGDHRVGHRVAQVRLGDLLHLGQHHGRDLLGVECLGLPLVAHLHLGLVSVVDHLERPVLHVRLHHRVAELAANQPLRVKHGVLRVQRHLVLGRIPDEPLGVREGHVAGGGAVALVVGDDLHLAVLEHPHAGVGGAQVDADRRGFGHAGSLLSVGSALRLGAGNGTLDPREELGPSGRRKLDAPVPAAAQVRSGKPWDNGSHSRKR | **none** |
| **ORF27** [187604 - 185628] (REVERSE SENSE) KALSQNSILFFLHRPQRTMATAKGIAIGIDLGTTYSCVGVFQHGKVEIIANDQGNRTTPSYVAFTDTERLIGDAAKNQVAMNPQNTVFDAKRLIGRKFNDPVVQADMKLWPFQVINEGGKPKVLVSYKGENKAFYPEEISSMVLTKLKETAEAFLGHPVTNAVITVPAYFNDSQRQATKDAGVIAGLNVLRIINEPTAAAIAYGLDKGGQGERHVLIFDLGGGTFDVSILTIDDGIFEVKATAGDTHLGGEDFDNRLVSHFVEEFKRKHKKDISQNKRAVRRLRTACERAKRTLSSSTQANLEIDSLYEGIDFYTSITRARFEELCADLFRGTLEPVEKALRDAKMDKAKIHDIVLVGGSTRIPKVQRLLQDYFNGRDLNKSINPDEAVAYGAAVQAAILMGDKSEKVQDLLLLDVAPLSLGLETAGGVMTALIKRNSTIPTKQTQIFTTYSDNQPGVLIQVYEGERAMTKDNNLLGRFDLTGIPPAPRGVPQIEVTFDIDANGILNVTATDKSTGKVNKITITNDKGRLSKEEIERMVLDAEKYKAEDEVQREKIAAKNALESYAFNMKSVVSDEGLKGKISESDKNKILDKCNELLSWLEVNQLAEKDEFDHKRKELEQMCNPIITKLYQGGCTGPACGTGYVPGRPATGPTIEEVD | **none** |
