# Supplementary material for: Genetic Determinants of Antibody Levels in Cerebrospinal Fluid in Multiple Sclerosis: Possible Links to Endogenous Retroviruses
Source: Int J Mol Sci. 2018 Mar 9;19(3):786. doi: 10.3390/ijms19030786 (PMC5877647; doi:10.3390/ijms19030786)
Supplement: Supplementary file 1 [file ijms-19-00786-s001.zip › Supplementary Table S6.docx]

**Supplementary Table S6. Genes, pseudogenes, and large retroviral ORFs flanking rs9271640/rs6457617 on chromosome 6**. Presented are all annotated genes and the identified ORFs in the 2,071,651 base pair region (nucleotide accession: NC_000006.12:31624423-33696074) surrounding the SNP rs9271640/ rs6457617 region. Genes/ORFs located on the reverse strand are indicated by the orientation “c”). ORF15 was numbered according to Supplementary Table S5.

| **Orientation** | **Start** | **Stop** | **Gene symbol** | **Feature** |
| --- | --- | --- | --- | --- |
|  |  |  |  |  |
|  | <1 | 13358 | PRRC2A | Feature |
|  | 9365 | 9436 | MIR6832 |  |
| c | 14606 | 36478 | BAG6 |  |
|  | 27988 | 33788 | APOM |  |
| c | 33876 | 36350 | C6orf47 |  |
| c | 36807 | 41861 | GPANK1 |  |
|  | 41458 | 45648 | CSNK2B |  |
|  | 46529 | 48028 | LY6G5B |  |
|  | 52262 | 55951 | LY6G5C |  |
|  | 58473 | 62698 | LOC105375019 |  |
| c | 62527 | 78938 | ABHD16A |  |
|  | 65289 | 66894 | LOC105375018 |  |
| c | 76607 | 76669 | MIR4646 |  |
|  | 82485 | 86173 | LY6G6F |  |
| c | 87554 | 89643 | LY6G6E |  |
|  | 90934 | 93382 | LY6G6D |  |
| c | 94226 | 97312 | LY6G6C |  |
|  | 96135 | 102288 | C6orf25 |  |
| c | 102615 | 105843 | DDAH2 |  |
| c | 106159 | 112896 | CLIC1 |  |
| c | 113010 | 115513 | LOC105375020 |  |
|  | 115526 | 140428 | MSH5-SAPCD1 |  |
|  | 115526 | 138256 | MSH5 |  |
| c | 132529 | 132631 | RNU6-850P |  |
|  | 138574 | 140428 | SAPCD1 |  |
| c | 139713 | 152909 | VWA7 |  |
| c | 139713 | 141166 | SAPCD1-AS1 |  |
| c | 153096 | 171513 | VARS |  |
| c | 172970 | 182562 | LSM2 |  |
| c | 185197 | 190636 | HSPA1L |  |
|  | 191092 | 193520 | HSPA1A |  |
|  | 203313 | 205832 | HSPA1B |  |
|  | 210493 | 215344 | C6orf48 |  |
|  | 210841 | 210904 | SNORD48 |  |
|  | 212654 | 212717 | SNORD52 |  |
| c | 234630 | 238510 | NEU1 |  |
| c | 238770 | 254624 | SLC44A4 |  |
| c | 255337 | 273285 | EHMT2 |  |
|  | 256434 | 259782 | LOC107986588 |  |
|  | 273363 | 321252 | C2 |  |
| c | 274772 | 277909 | ZBTB12 |  |
| c | 310052 | 317302 | C2-AS1 |  |
|  | 321522 | 327662 | CFB |  |
| c | 327665 | 334665 | NELFE |  |
| c | 332417 | 332518 | MIR1236 |  |
|  | 334382 | 345430 | SKIV2L |  |
| c | 345389 | 347868 | DXO |  |
|  | 346753 | 357024 | STK19 |  |
|  | 357635 | 378258 | C4A |  |
|  | 381214 | 384487 | CYP21A1P |  |
| c | 383998 | 388601 | TNXA |  |
|  | 388968 | 389762 | STK19B |  |
|  | 390373 | 410996 | C4B |  |
|  | 413894 | 417248 | CYP21A2 |  |
| c | 416733 | 484952 | TNXB |  |
| c | 454086 | 454206 | RNA5SP206 |  |
|  | 490846 | 503818 | ATF6B |  |
| c | 504285 | 505868 | FKBPL |  |
| c | 523941 | 527521 | PRRT1 |  |
| c | 528380 | 529943 | LOC100507547 |  |
|  | 529030 | 539259 | PPT2 |  |
|  | 529577 | 543863 | PPT2-EGFL8 |  |
|  | 540183 | 543863 | EGFL8 |  |
| c | 543784 | 553689 | AGPAT1 |  |
| c | 545608 | 545694 | MIR6721 |  |
|  | 553963 | 556371 | RNF5 |  |
|  | 555394 | 555454 | MIR6833 |  |
| c | 556546 | 560825 | AGER |  |
| c | 560311 | 565764 | PBX2 |  |
| c | 566344 | 571101 | GPSM3 |  |
| c | 570421 | 599645 | NOTCH4 |  |
|  | 630751 | 783308 | LOC101929163 |  |
| c | 666101 | 747490 | C6orf10 |  |
|  | 700776 | 702099 | HNRNPA1P2 |  |
|  | 728455 | 728561 | RNU6-603P |  |
|  | 766088 | 769269 | HCG23 |  |
| c | 768917 | 784457 | BTNL2 |  |
|  | 815420 | 820624 | HLA-DRA |  |
| c | 835398 | 835667 | HLA-DRB9 |  |
|  | 892921 | 905894 | HLA-DRB5 |  |
| c | 925518 | 925668 | RNU1-61P |  |
| c | 928291 | 935580 | HLA-DRB6 |  |
| c | 935582 | 936583 | - | **ORF15** |
| c | 951855 | 952001 | LOC107521952 |  |
| c | 954347 | 965414 | HLA-DRB1 |  |
|  | 1000001 | 1000001 | - | rs9271640 |
| c | 1012766 | 1018251 | LOC107986589 |  |
|  | 1012981 | 1030424 | HLA-DQA1 |  |
| c | 1035042 | 1042267 | HLA-DQB1 |  |
|  | 1071651 | 1071651 | - | rs6457617 |
| c | 1081722 | 1082381 | MTCO3P1 |  |
|  | 1093745 | 1094405 | LOC102725019 |  |
|  | 1116964 | 1122465 | HLA-DQA2 |  |
| c | 1125490 | 1125557 | MIR3135B |  |
| c | 1131672 | 1139131 | HLA-DQB2 |  |
| c | 1188341 | 1192626 | HLA-DOB |  |
| c | 1197411 | 1214401 | TAP2 |  |
| c | 1216295 | 1220513 | PSMB8 |  |
|  | 1219664 | 1222078 | PSMB8-AS1 |  |
| c | 1220787 | 1229549 | TAP1 |  |
|  | 1229739 | 1235429 | PSMB9 |  |
|  | 1252053 | 1255652 | PPP1R2P1 |  |
|  | 1269754 | 1279336 | LOC100294145 |  |
|  | 1271980 | 1272067 | HLA-Z |  |
| c | 1310207 | 1316648 | HLA-DMB |  |
| c | 1324192 | 1328700 | HLA-DMA |  |
|  | 1344238 | 1357083 | BRD2 |  |
| c | 1379760 | 1385190 | HLA-DOA |  |
| c | 1440147 | 1456356 | HLA-DPA1 |  |
|  | 1451504 | 1465274 | HLA-DPB1 |  |
|  | 1454877 | 1455589 | RPL32P1 |  |
| c | 1467060 | 1468892 | HLA-DPA2 |  |
| c | 1479271 | 1482722 | COL11A2P1 |  |
|  | 1488094 | 1504691 | HLA-DPB2 |  |
| c | 1506585 | 1519133 | LOC105375021 |  |
| c | 1506775 | 1506921 | HLA-DPA3 |  |
|  | 1519091 | 1524378 | HCG24 |  |
| c | 1538270 | 1568587 | COL11A2 |  |
| c | 1569163 | 1576575 | RXRB |  |
|  | 1575179 | 1575270 | RNY4P10 |  |
|  | 1576404 | 1580015 | SLC39A7 |  |
|  | 1580215 | 1582409 | HSD17B8 |  |
|  | 1583413 | 1583522 | MIR219A1 |  |
|  | 1584087 | 1588300 | RING1 |  |
|  | 1591152 | 1591761 | ZNF70P1 |  |
|  | 1621653 | 1622664 | LOC105375022 |  |
|  | 1625114 | 1630468 | HCG25 |  |
| c | 1625850 | 1647543 | VPS52 |  |
|  | 1647653 | 1652082 | RPS18 |  |
|  | 1652718 | 1654403 | B3GALT4 |  |
| c | 1654428 | 1665105 | WDR46 |  |
| c | 1662805 | 1662867 | MIR6873 |  |
|  | 1665175 | 1666512 | PFDN6 |  |
|  | 1665823 | 1665903 | MIR6834 |  |
|  | 1667232 | 1674966 | RGL2 |  |
| c | 1675272 | 1689965 | TAPBP |  |
| c | 1689983 | 1693520 | ZBTB22 |  |
| c | 1694136 | 1698594 | DAXX |  |
|  | 1714470 | 1715273 | MYL8P |  |
| c | 1740302 | 1741940 | LYPLA2P1 |  |
| c | 1764934 | 1765057 | RPL35AP4 |  |
|  | 1767102 | 1785804 | KIFC1 |  |
| c | 1775593 | 1776222 | RPL12P1 |  |
|  | 1786329 | 1792031 | PHF1 |  |
| c | 1792120 | 1793866 | CUTA |  |
|  | 1795648 | 1829267 | SYNGAP1 |  |
|  | 1813909 | 1814015 | MIR5004 |  |
|  | 1830157 | 1833122 | ZBTB9 |  |
| c | 1892506 | 1911565 | LOC107986537 |  |
|  | 1916276 | 1916578 | RN7SL26P |  |
| c | 1948124 | 1955871 | BAK1 |  |
|  | 1959277 | 1964604 | GGNBP1 |  |
| c | 1961684 | 1968916 | LINC00336 |  |
|  | 1996911 | >2071652 | ITPR3 |  |
| c | 2006822 | 2009323 | LOC101929188 |  |
|  | 2009454 | 2011138 | LOC105375023 |  |
